# Optimal dimensioning of grid-connected PV/wind hybrid renewable energy systems with battery and supercapacitor storage a statistical validation of meta-heuristic algorithm performance

**DOI:** 10.1038/s41598-025-28234-9

**Published:** 2025-12-29

**Authors:** Mohamed Mahmoud Samy, Aykut Fatih Güven

**Affiliations:** 1 Department of Electrical Engineering, Beni-Suef University, Beni-Suef, Egypt; 2 Department of Electrical and Electronics Engineering, Yalova University, Yalova, Turkey

**Keywords:** Energy optimization, Meta-Heuristic algorithms, Supercapacitors, Statistical analysis, Renewable energy design

## Abstract

The increasing environmental and economic drawbacks of fossil fuels have accelerated the global transition to renewable energy sources. In this context, the optimal design of hybrid renewable energy systems (HRES) that combine solar, wind, and energy storage technologies is critical for achieving sustainable and cost-effective power generation. This study addresses the problem of optimally sizing a grid-connected HRES composed of photovoltaic (PV) panels, wind turbine (WTs), batteries (BTs), and supercapacitors (SCs). A mathematical model is developed to minimize the annual cost of the system (ACS) while ensuring high renewable energy utilization and system efficiency. To solve this optimization problem, five advanced meta-heuristic algorithms—Hunger Games Search (HGS), Spider Wasp Optimizer (SWO), Kepler Optimization Algorithm (KOA), Fire Hawk Optimizer (FHO), and Coronavirus Disease Optimization Algorithm (COVIDOA)—were applied and statistically validated. The model was tested on real meteorological and load data from a university campus in Turkey. Results show that HGS achieved the most favorable performance, with an ACS of $603,538.44, a cost of energy (COE) of $0.23801/kWh, and a renewable energy fraction (REF) of 80.04%. This configuration offers significant economic advantages compared to purchasing electricity directly from the grid at $0.35/kWh. The proposed system proves commercially viable for large consumers and demonstrates the practical effectiveness of meta-heuristic methods in energy system design. MATLAB was used for simulation, while R programming was employed for statistical validation of the algorithmic performance. The study establishes a reproducible and validated framework that can guide future research and implementation in the field of hybrid energy optimization.

**Supplementary Information:**

The online version contains supplementary material available at 10.1038/s41598-025-28234-9.

## Introduction

**Fig. 1:**
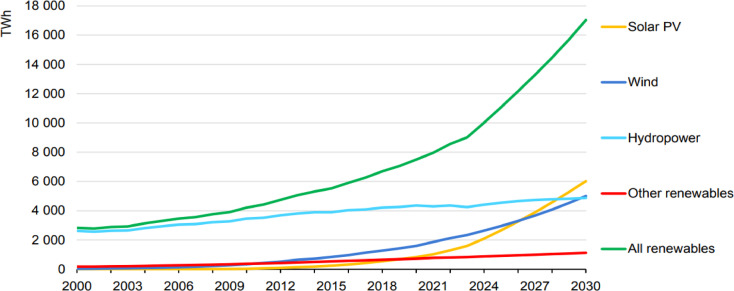
Global electricity generation by technology from 2000 to 2030 (*Source* IEA, Renewables 2024).

The rapid increase in global energy demand and the need to reduce carbon emissions due to climate change have accelerated the integration of renewable energy sources (RESs) into power grids^1^. Among these sources, solar and wind energy have gained widespread adoption due to their economic viability and environmental sustainability^2^. However, the inherent variability and intermittency of these resources necessitate advanced storage solutions to maintain grid stability and reliability^3^. According to the latest International Energy Agency (IEA) report, global renewable electricity generation reached approximately 9,000 TWh by the end of 2024, primarily driven by solar PV and wind energy. This trend is expected to accelerate, with renewable sources projected to dominate new electricity generation by 2030 (Fig. 1)^4^.

The increasing penetration of RESs has led to advancements in hybrid renewable energy systems (HRES), facilitating their widespread adoption. These systems integrate distributed generators (DGs), energy storage systems (ESSs), and flexible loads, allowing operation both in grid-connected and off-grid modes with advanced control mechanisms^5^. As a key component of future smart grids, HRES provide a more efficient and environmentally friendly alternative to conventional power plants, reducing power losses and grid congestion while enhancing power quality and reliability^6^.

To address the challenges posed by RES intermittency, energy storage systems (ESSs) are utilized to store excess energy during periods of surplus production and release it when demand arises. The most widely used form of ESS is battery energy storage systems (BESS)^7^. However, while batteries are effective in meeting steady loads, they often struggle to handle the rapid fluctuations characteristic of RES output^8^. This limitation has led to the emergence of hybrid energy storage systems (HESS), which integrate multiple storage technologies, such as supercapacitors (SCs) and batteries (BTs), to enhance system performance and stability^9^.

Hybrid systems achieve enhanced performance and efficiency through the complementary roles of batteries and supercapacitors. Batteries, particularly lithium-ion variants, ensure long-term energy storage and stable power delivery, making them essential for applications in renewable energy systems, electric vehicles, and backup power solutions^10^. They facilitate energy recuperation and provide reliable energy supply, particularly in electric vehicles, where stability is crucial. Supercapacitors, on the other hand, are distinguished by their high-power density, rapid charge–discharge cycles, and long operational lifespan^11^. They primarily support transient operations, compensating for short-term power fluctuations in hybrid systems. Their superior efficiency has led to increasing adoption in industries such as transportation and aerospace, where rapid energy delivery and system stability are critical.

By working in synergy, batteries and supercapacitors significantly enhance hybrid system efficiency. Batteries enable the integration of RESs by providing a steady power source, while supercapacitors improve overall system responsiveness by enabling fast energy exchanges, increasing power density, and extending the lifespan of the battery. This hybrid approach leads to a more balanced and optimized energy storage solution, making the system more adaptable to varying load conditions^12^.

Supercapacitor energy storage systems (SESS) play a crucial role in mitigating rapid power variations in batteries, thereby enhancing battery longevity. Operational parameters such as the state of charge (SOC) and depth of discharge (DOD) are critical in ensuring system efficiency. Discharging the ESS below its recommended DOD threshold leads to performance degradation and increased costs^13^. Therefore, effective power management strategies are required to prevent excessive battery discharge. Robust, rule-based optimization schemes are essential for achieving optimal system sizing and coordinated power flow in hybrid energy systems^14^. These advanced methodologies ensure that system components are configured efficiently to meet dynamic load demands.

The structure of HRES is inherently complex due to the diverse nature of its components, which include energy generation, storage, and distribution units, along with automation and control mechanisms. The optimization of these systems involves numerous nonlinear constraints, multi-objective functions, and a mix of discrete and continuous variables. Selecting appropriate optimization methods is crucial for ensuring system efficiency. Various approaches, including precise mathematical methods and meta-heuristic algorithms, are employed in HRES optimization. Additionally, widely used commercial software tools such as HOMER, HOMER Pro, and i-HOGA facilitate system modeling and analysis. The accuracy of these optimization strategies is critical for achieving convergence and ensuring the reliability of hybrid energy systems.

This research investigates the intricate dynamics of integrating batteries and supercapacitors within HRES, exploring their implications for system efficiency, reliability, and stability in response to the intermittency of renewable energy sources. This study employs sophisticated mathematical modeling techniques to analyze the interactions between solar, wind, battery, and supercapacitor components. Additionally, a detailed sensitivity analysis is conducted to evaluate the impact of load variations and supercapacitor charging efficiency on overall system performance. By addressing the complexities of power management strategies and utilizing advanced optimization algorithms, this research aims to maximize the operational potential of hybrid energy storage systems. The study further examines the effects of load fluctuations on system sizing and cost through comprehensive sensitivity analyses, providing deeper insight into the performance of the proposed hybrid system under different operating conditions.

Meta-heuristic optimization methods are widely used in HRES research, allowing efficient exploration of solution spaces close to the global optimum^15^. Consequently, precise system sizing and optimization are essential to meet expected load demands while maintaining efficiency and reliability^16^. Numerous studies have explored various optimization techniques for HRES, considering different configurations that include grid-connected and off-grid solar, wind, diesel generators, batteries, supercapacitors, and hydrogen storage systems. Table 1 provides a detailed overview of various HRES configurations, and the optimization algorithms applied in recent studies.

**Table 1:** Review of the various components and optimization algorithms used in current research on HRES.

System type	Energy resources	Energy storage	Optimization algorithms	Country	References
On Grid	Photovoltaic (PV)/Wind turbine (WT)	Battery (BT)	Moth-flame optimization algorithm (MFOA)	New Zealand	^17^
Off Grid	PV/WT	BT, Supercapacitor (SC)	Simulated annealing particle swarm optimization (SAPSO)	China	^18^
On Grid	PV/WT/	BT, SC	Particle swarm optimization (PSO)	Saudi Arabia	^19^
Off Grid	WT	BT, SC	Non-dominated sorting genetic algorithm II	China	^20^
Off Grid	PV/WT	BT	artificial hummingbird algorithm	New Zealand	^21^
Off Grid	PV/WT	BT/SC/Hydrogen(H_2_)	PSO, Genetic algorithm (GA), Artificial bee colony (ABC), Ant colony Optimizer (ACO)	Kenya	^22^
On Grid	PV/WT/Bioenergy	BT	multi-objective PSO, multiobjective grasshopper optimization algorithm	Japan	^23^
On Grid	PV/WT	BT	Ant lion optimizer, PSO, cuckoo search CS)	Malaysia	^24^
Off Grid	PV/WT	BT/(H_2_)	equilibrium optimizer (EQ), bat optimization (BAT), and black-hole-based optimization (BHB) algorithms	Egypt	^25^
Off Grid	PV/WT/Diesel generator (DG)	BT	GA	Nigeria	^26^
Off Grid	PV/WT	BT/SC	MFOA, sine–cosine algorithm (SCA) , multi-verse optimizer, water evaporation optimization	New Zealand	^27^
Off Grid	PV/WT	BT	MFOA, GA, PSO, and hybrid GA-PSO	Iran	^28^
Off Grid	PV/WT	BT	Harmony search algorithm (HSA)	China	^29^
Off Grid	PV/WT/DG/Fuelcell(FC)/Electrolyzer(Elz)	H_2_ tank	Multi-objective crow search algorithm	Iran	^30^
On Grid	PW/WT	BT	Based on position-mutation gray wolf optimization (PM-GWO)	China	^31^
Off grid	PV/WT/Biomass Gasifier (BG)/FC/Elz	BT/H_2_ tank	Levy flight-salp swarm algorithms (LF-SSA), SSA, GA, and HOMER	Nigeria	^32^
Off grid	PV/WT/DG	BT	HSA, Jaya algorithms, ACO, and HOMER	Turkey	^33^
Off grid	PW/WT/BG/FC/Elz	H_2_ tank	HFGA, FA, GA, SCA, and CS algorithms	Turkey	^34^
Off grid	PV/WT/DG	BT	GA, firefly algorithm (FA), PSO, and a novel hybrid of the firefly and PSO algorithms (HFAPSO)	Turkey	^35^
Off grid	PW/WT//BG/FC/Elz	H_2_ tank	Atom search optimization (ASO), ACO L´evy Flight, Flower Pollination (FPA), BAT, Ant Lion Optimization (ALO), Jaya, Simulated Annealing (SA), Whale Optimization Algorithm (WOA), Gradient Based Optimizer (GBO), and Henry Gas Solubility Optimization (HGSO) algorithm	Turkey	^36^

The research provides a comprehensive evaluation of advanced optimization algorithms developed for HESS integration within HRES. The proposed methodology includes a comparative analysis of several meta-heuristic algorithms that have not been previously applied to HRES configurations, offering methodological advantages over conventional approaches. Evaluation criteria focus on system stability, computational efficiency, and scalability. The optimization process involves rigorous testing of five algorithms: Hunger Games Search (HGS), Spider Wasp Optimizer (SWO), Kepler Optimization Algorithm (KOA), Fire Hawk Optimizer (FHO), and Coronavirus Disease Optimization Algorithm (COVIDOA), with performance metrics validated through statistical analysis.

The HGS algorithm generated the most economically and technically efficient system configuration. The resulting design achieved an annual system cost of $603,538.44, a cost of energy of $0.23801/kWh, and a renewable energy fraction of 80.04%. The configuration demonstrated superior performance compared to the other optimization methods and showed strong potential for commercial deployment under grid-connected conditions.

To clearly define the purpose of this study, the main objectives are summarized as follows:

- To model and optimize a grid-connected HRES integrating photovoltaic panels, wind turbines, batteries, and supercapacitors.
- To apply and comparatively evaluate five advanced meta-heuristic algorithms (HGS, SWO, KOA, FHO, and COVIDOA) that have not previously been utilized for HRES optimization.
- To assess each algorithm’s performance based on economic and technical metrics such as annual system cost of system (ACS), cost of energy (COE), and renewable energy fraction (REF).
- To statistically validate the optimization results and demonstrate the computational efficiency of the proposed methods under real-world conditions.
- To offer a transferable modeling framework and provide insights for future applications in commercial energy systems.

The analysis of optimization algorithms contributes valuable knowledge regarding algorithmic performance under real-world constraints in hybrid energy systems. The findings support the development of more reliable, cost-effective, and environmentally sustainable energy system designs. The validated modeling structure offers a transferable framework for future research and implementation.

The structure of the paper is organized as follows: Sect. 2 presents the modeling of the hybrid energy generation system and its renewable components. Section “Objective function” details the adopted optimization approach, system representation, load characteristics, and control strategies. Section “Methodology” discusses the simulation results for the PV/WT/BT/SC system. Section "Simulation results and discussion" concludes the study and outlines future research directions.

## Mathematical representation of the hybrid renewable energy system

**Fig. 2:**
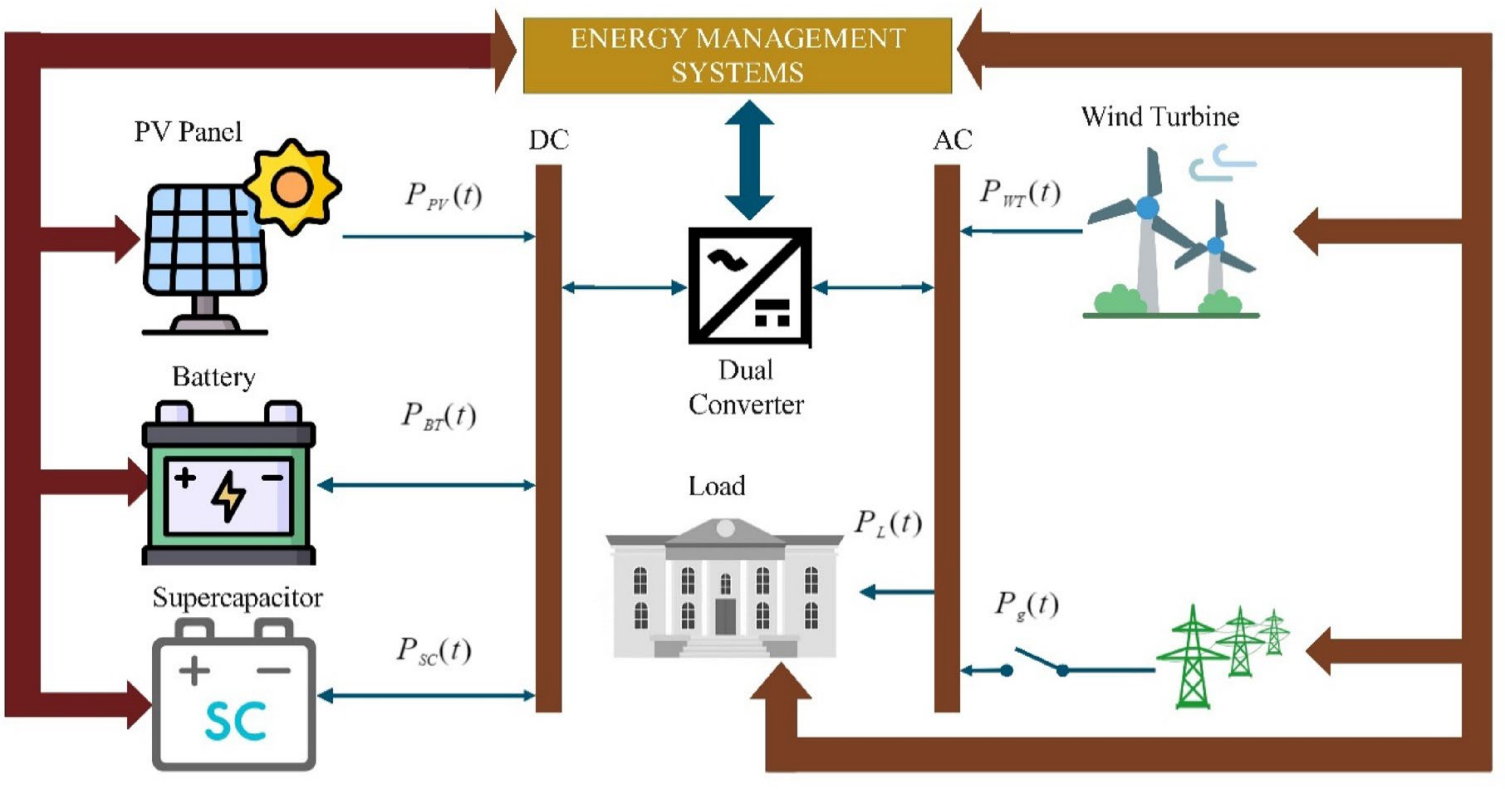
Grid-connected PV/WT/BT/SC model proposed by researchers for this research area.

Energy management systems are becoming increasingly sophisticated and integrated, driven by the dual imperatives of sustainability and improved energy efficiency. In this study, the proposed HRES is conceptualized as a microgrid that incorporates diverse energy generation and storage components, including PVs, WTs, BTs, and SCs, as depicted in Fig. 2. This figure provides a schematic overview of the interactions among these components and the associated loads. At the core of the system is a bidirectional converter, responsible for managing the energy exchange between the direct current (DC) and alternating current (AC) elements. This configuration mitigates the inherent variability of renewable energy sources, thereby enhancing energy security and ensuring a stable supply^37,38^. The following sections will present the mathematical formulations of each component and outline the modeling strategies used to optimize the energy management system.

### Meteorological and electrical load data

**Fig. 3:**
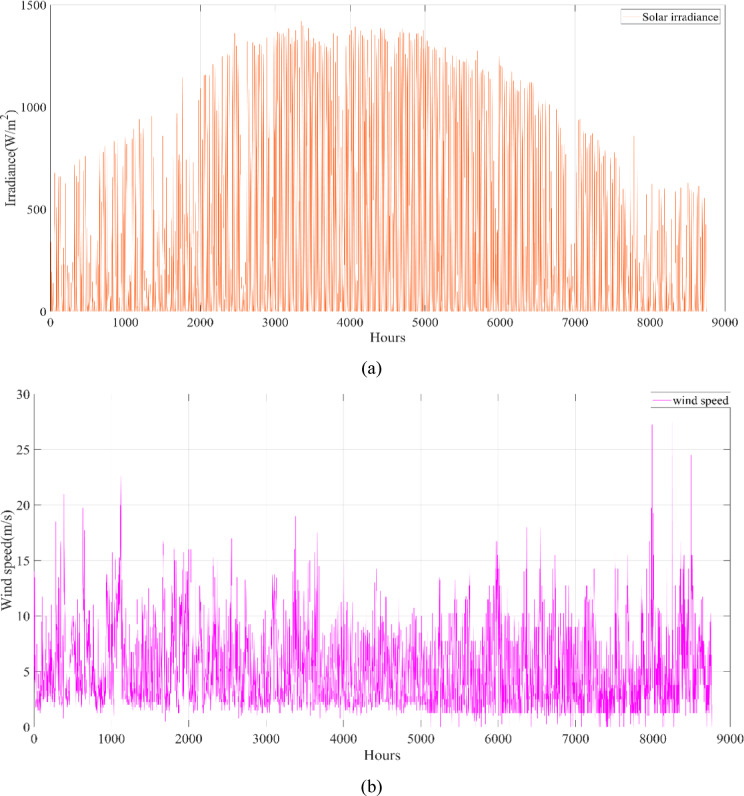

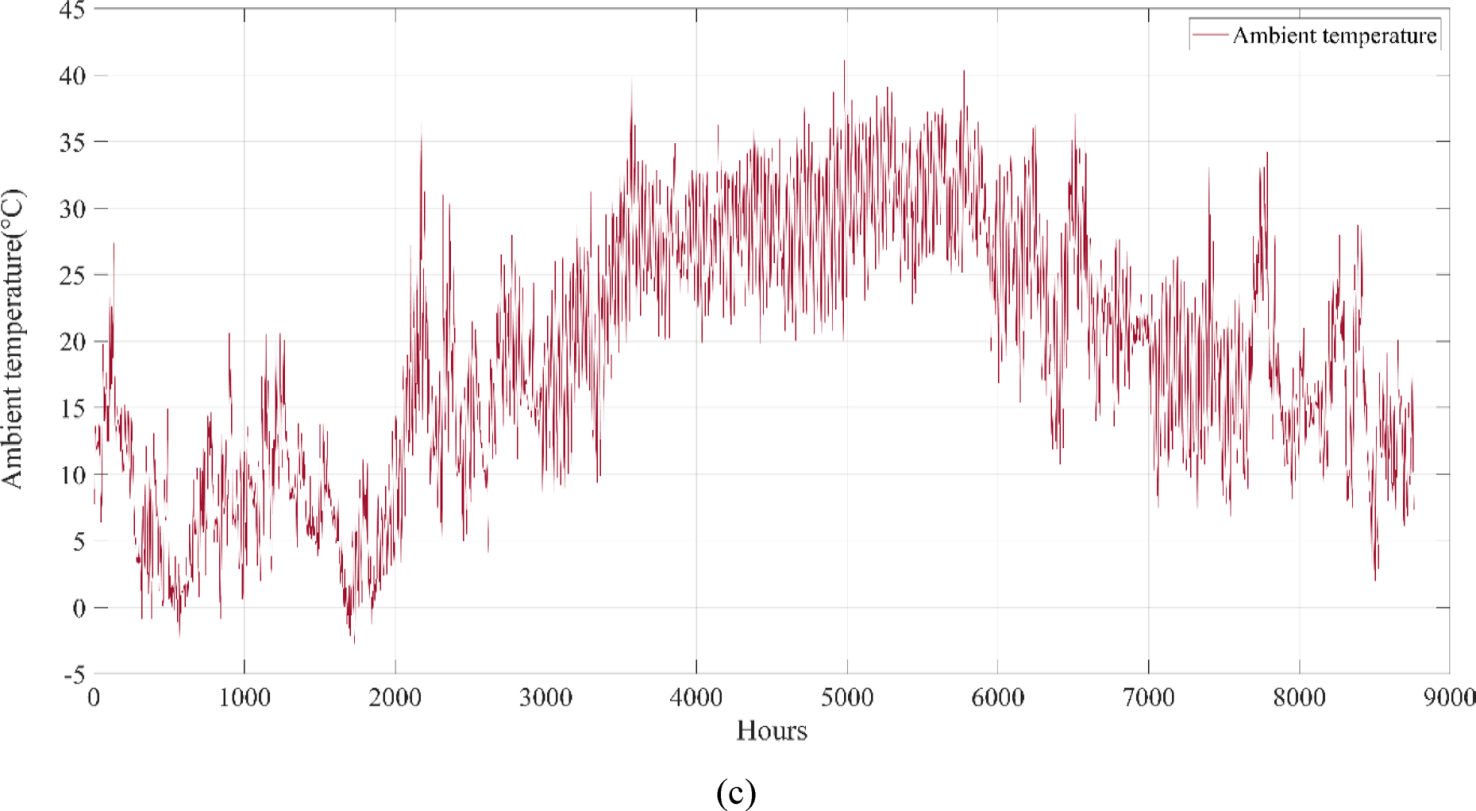
Weather patterns of the study area. (a) solar radiation $(\mathrm{W}/{\mathrm{m}}^{2})$, (b) wind speed (m/s), and (c) ambient air temperature ($^\circ{\rm C}$).

**Fig. 4:**
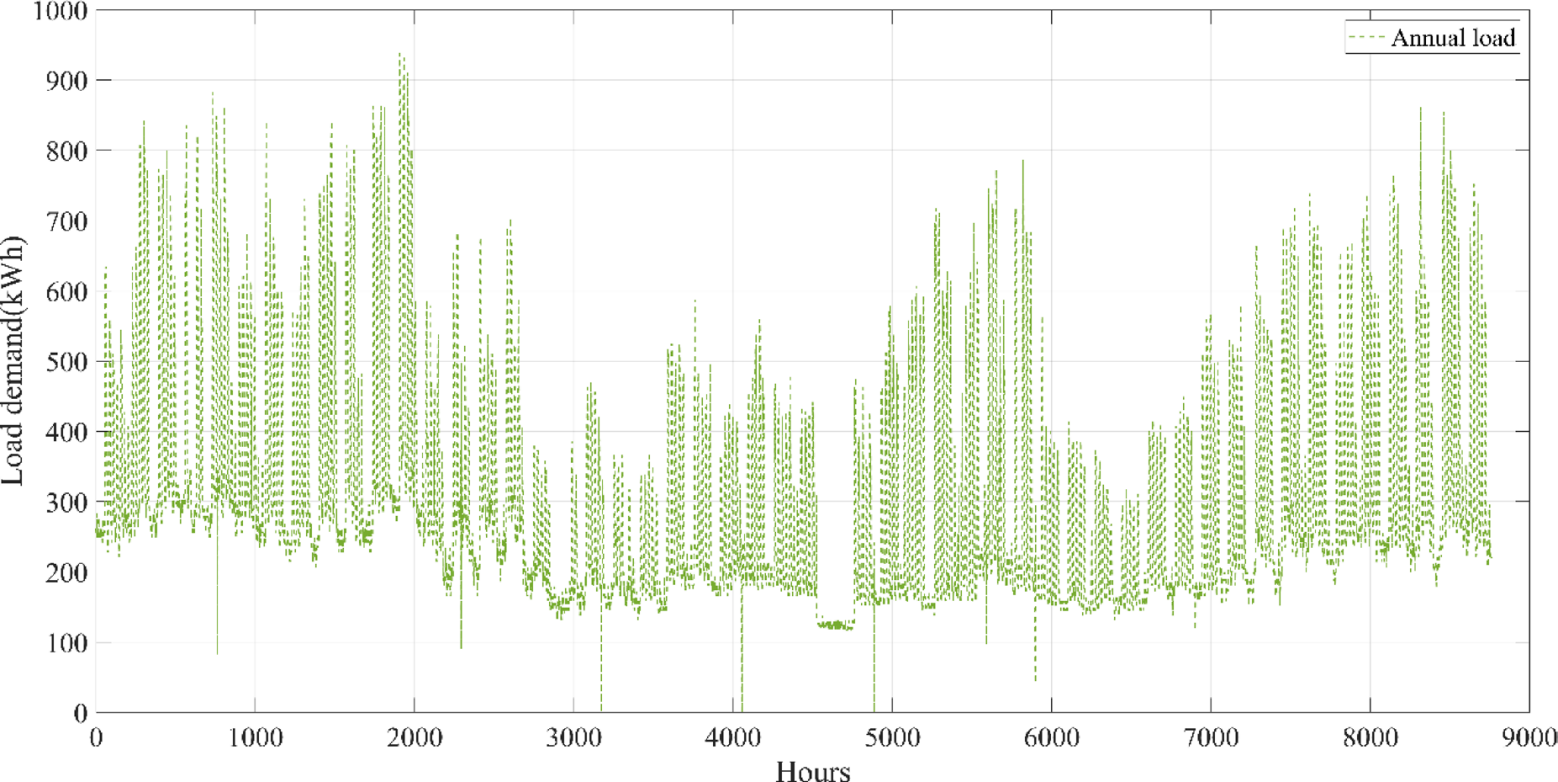
Annual load demand profile for the campus.

A university campus in Turkey at a latitude of 40°39.2°N and a longitude of 29°13.2°E was chosen for the HRES proposed in this study. The Turkish State Meteorological Service provided the meteorological information used in this study. Using hourly data on solar radiation, wind speed, ambient air temperature, and load based on annual data, a profile of the study area was created. These data were obtained from the Turkish State Meteorological Service for the period 01/01/2023 – 31/12/2023 on an hourly basis. Figure 3 shows the wind speed at 10 m above ground level, ambient air temperature, and solar radiation profiles per hour over a year (8760 h). Figure 4 shows the load demand profile for the selected case study. The average energy consumption per day on the campus was 6947.06 kWh. Throughout the year, the maximum and minimum typical hourly loads (per hour) were 938.50 kW and 289.46 kW, respectively. However, as seen in Fig. 4, instantaneous hourly load values may fluctuate below this typical minimum due to short-term variations in electricity demand.

### PV component modeling

To effectively simulate the performance characteristics of a PV component, it is critical to consider its maximum power output tendencies. The capacity of the PV module to generate optimal power is subject to several influencing elements^39^. These include the intensity of solar irradiance received by the panel, the established orientation of the solar panel, the characteristics of the PV modules, and the local air temperature at specific times. In this study, Eq. (1), which is a refined model incorporating solar irradiance and ambient temperature, was used to determine the annual power output from the PV generator^40^.

1 $${P}_{PV-out}={\sum }_{t=1}^{8760}\left({P}_{PVr}{N}_{pv}{PV}_{df}\right)\times \left(\frac{G\left(t\right)}{{G}_{base}}\right)\times \left(1+{K}_{T}\left(\left({T}_{amb}\left(t\right)+G\left(t\right)\times 1000\times \left(\frac{NOCT-20}{800}\right)\right)-{T}_{base}\right)\right)$$

In this formulation, ${P}_{PV-out}$ denotes the photovoltaic output power (kW), while ${P}_{PVr}$​ represents the rated power of the photovoltaic system (kW). ${N}_{pv}$ refers to the number of installed photovoltaic panels, and ${PV}_{df}$​ is the derating factor, which accounts for real-world inefficiencies such as wiring losses, accumulation of dust or snow, and shading, all of which reduce the system’s output under actual operating conditions^41^. The solar irradiance on the tilted plane (kW/m^2^) is represented by $G$, with ${G}_{base}$​ signifying the standard irradiance level of 1 kW/m^2^ at 25 °C^42^. The power output’s sensitivity to temperature is expressed by the temperature coefficient, ${K}_{T}$​, defined as ${K}_{T}$ = − 3.7 × 10^–3^(1/°C). ${T}_{amb}$​ corresponds to the ambient temperature (°C), ${T}_{base}$​ denotes the reference temperature of 25 °C, and $NOCT$ refers to the nominal operating cell temperature (°C) under standard testing conditions^28^.

### WT component modeling

Wind is a highly valuable and promising source for HRES when accurately modeled and managed. The energy yield from the wind is influenced by various factors, including the hub height of the turbine, the characteristics of the local terrain, and turbine speed^43^. These factors collectively affect the energy output of the WT at a specific location. The turbine’s elevation above the standard ground level plays a crucial role in power generation^44^. An equation for adjusting the turbine’s height can be applied during the installation process. To calculate the energy output from the WT, Eq. (2) is used^45^.

2 $$P_{WT} = \left\{ {\begin{array}{*{20}l} {\eta_{w} \times { }P_{WTr} \times N_{WT} \times \mathop \sum \limits_{t = 1}^{8760} \left( {\frac{{V\left( t \right)^{3} - V_{ci}^{3} }}{{V_{r}^{3} - V_{ci}^{3} }}} \right),} \hfill & {V \le V_{r} } \hfill \\ {\eta_{w} \times P_{WTr} \times N_{WT} , } \hfill & {V_{r} \le V \le V_{co} } \hfill \\ {0,} \hfill & {V_{co} \le V or V \le V_{ci} } \hfill \\ \end{array} } \right.$$

Equation (2) considers various factors to estimate the WT output power (${P}_{WT}$), where the WT efficiency (${\eta }_{w}$), number of WTs (${N}_{WT}$), estimated power of the WT (${P}_{WTr}$), evaluated wind speed (V(t)), cut in wind speed (${V}_{ci}$), terminal speed of wind (${V}_{co}$), and estimated wind speed (${V}_{r}$) were all considered. In this study, the wind turbine efficiency (${\eta }_{w}$) was set to 0.35 and incorporated into the optimization model accordingly. This ensures a realistic estimation of wind power generation within the proposed framework^46^.

Furthermore, the wind turbine (WT) hub height (${H}_{WT}$), base altitude (${H}_{r}$), wind speed at the reference height (${V}_{t}$), and friction parameter (α) were integral parameters considered in this study, as outlined in Eq. (3). Specifically, for surfaces characterized by smoothness and a vast open area, the $\alpha$ value was set to 1/7. This consideration is critical for accurately modeling the wind profile and energy potential, as described in the Eq. (3)^36^.

3 $${V}_{t}= {V}_{r}\times {\left(\frac{{H}_{WT}}{{H}_{r}}\right)}^{\alpha }$$

### Battery component modeling in microgrids

ESS within a microgrid is critical for maintaining grid stability and efficiency. The SOC is a key operational parameter for the ESS, requiring management within a defined operational range to ensure the grid’s overall integrity and optimal performance^47^.

#### Operational range of the SOC

To ensure the durability and operational health of the ESS, it is essential to maintain the SOC within a designated range. This specific range is delineated in Eq. (4).

4 $${SOC}_{min}\le SOC\le {SOC}_{max}$$

The ${SOC}_{min}$​ and ${SOC}_{max}$​ parameters establish the minimum and maximum SOC limits, setting the safe operating range for the energy storage system. These boundaries are essential to avoid overcharging and excessive discharging, both of which can negatively impact the battery’s operational efficiency and overall lifespan. Maintaining the SOC within these limits is crucial for ensuring the long-term durability and optimal functionality of the battery^48^.

#### SOC dynamics

The SOC at any given time is dependent on charging and discharging activities within the system. The SOC at a future time $j+\Delta j$ is determined by Eq. (5), which accounts for the energy that is either stored or expended during the time interval $\Delta j$.

5 $${SOC}_{j+\Delta j}={SOC}_{j}-{P}_{ES-j}\times \frac{\Delta j}{{C}_{ES}}$$

The variable ${P}_{ES-j}$​ represents the rate of energy transfer into (during charging) or out of (during discharging) the energy storage system in the interval from time $j$ to $j+\Delta j$, while ${C}_{ES}$​ indicates the total energy capacity of the energy storage system, defining its maximum energy storage potential^19^.

#### Charging and discharge models

To enhance the performance and reliability of microgrids, dynamic management of the energy balance in energy storage systems (ESS) is crucial. This management is articulated through two fundamental equations that reflect charging and discharging events. As outlined in Eq. (6), during the charging process, the battery’s energy balance is updated as follows^49^:

6 $$Eb\left(t+\Delta t\right)=Eb\left(t\right)+\Delta t{P}_{b}^{charging}{\eta }_{charging}$$

In this equation, $Eb\left(t\right)$ represents the energy balance at the current time $t$, and $Eb\left(t+\Delta t\right)$ is the energy balance at the subsequent time step. The term ${P}_{b}^{charging}$​ denotes the power used to charge the system. This charging power is multiplied by the time interval $\Delta t$, which signifies a small step forward in time, and by the charging efficiency ${\eta }_{charging}$. The charging efficiency factor accounts for any losses or inefficiencies in the charging process. By multiplying the charging power by the time interval and efficiency, the equation calculates the increase in energy within the system due to charging over this period. This addition is then added to the current energy balance, yielding the energy balance at the next step. This model is essential for tracking the change in energy levels of the storage system during charging and for understanding how efficiently the system converts incoming power into stored energy.

In contrast, as specified in Eq. (7), the energy balance during the discharge process is adjusted in the following manner:

7 $$Eb\left(t+\Delta t\right)=Eb\left(t\right)-\Delta t\frac{{P}_{b}^{discharging}}{{\eta }_{discharging}}$$

In Eq. 8, $Eb\left(t\right)$ signifies the energy balance of a storage system at a particular time $t$, reflecting the net energy state at that moment. The term ${P}_{b}^{discharging}$​ is used to denote the power involved in the discharging process of the system. The efficiency of this discharge process is represented by ${\eta }_{discharging}$​. The equation works by calculating the energy balance at the forthcoming time step, $t+\Delta t$, which is achieved by deducting the energy used in the discharge process, adjusted for efficiency, from the current energy balance at time $t$. This approach to modeling is crucial for understanding the evolution of the energy balance within a storage system over time, particularly in terms of how discharge actions affect this balance^50^. This allows for a comprehensive understanding of the dynamic changes in energy within the system due to discharging activities. Both equations are integral to dynamically updating the battery’s state, ensuring that the ESS operates within its optimal parameters. This dynamic updating facilitates the effective balancing of charging and discharging cycles, thereby optimizing the efficiency and lifespan of the battery system within the microgrid^51^.

8 $$0{\le P}_{b}^{charging}{\le P}_{b}^{charging\_max}$$

9 $${0\le P}_{b}^{discharging}{\le P}_{b}^{discharging\_max}$$

The stored energy bounds are

10 $${E}_{b}^{min}\le Eb\left(t\right)\le {E}_{b}^{max}$$

Battery energy storage systems (BESS) operate within certain operational parameters to ensure safety and efficiency, as defined by Eqs. (8), (9), and (10). Specifically, the charging power, noted as ${P}_{b}^{charging}$​, is limited to a predefined maximum value ${P}_{b}^{charging\_max}$ to prevent overcharging, while the discharging power, ${P}_{b}^{discharging}$​, is similarly capped at ${P}_{b}^{discharging\_max}$​​ to avoid excessive depletion of the battery’s stored energy. Equation (10) sets the perimeters for the energy stored within the BESS, stipulating that it must remain above a minimum threshold ${E}_{b}^{min}$​ and below a maximum threshold ${E}_{b}^{max}$​This guarantees that the stored energy is maintained within a safe and functional range, thereby ensuring the system’s reliability and longevity. These parameters are crucial for the ongoing management of the BESS, allowing for efficient energy transfer while also preventing stress that can come from operating outside the designed capacity of the system.

### Supercapacitor component modeling

This section presents the modeling of the SC component, which is essential for advanced energy storage systems. Equation (11) describes the SC’s dynamic behavior, encompassing key aspects of its energy storage and release processes^19^.

11 $${E}_{SC}\left(t+\Delta t\right)= {E}_{SC}\left(t\right)+\eta \Delta t{P}_{SC}-\xi {E}_{SC}(t)$$

The SC’s energy storage dynamics are modeled using Eq. (11) and are pivotal in capturing the complex interplay of energy flow within the system. In this model, ${E}_{SC}\left(t\right)$ represents the energy stored in the SC at a given time $t$, essentially reflecting its state-of-charge. The efficiency factor $\eta$ plays a dual role, governing both the charging and discharging processes, and it indicates the SC’s ability to efficiently convert electrical energy to stored energy and vice versa. The time increment, denoted as $\Delta t$, is a critical factor in this model, highlighting the importance of temporal resolution in understanding energy storage dynamics. ${P}_{SC}$​, defining the power supplied to or drawn from the SC, illustrates the bidirectional nature of energy flow, where positive and negative values signify charging and discharging, respectively. Lastly, $\xi$, the self-discharge rate, is crucial for accounting for the energy losses when the SC is neither charging nor discharging, which is a key factor in long-term energy storage scenarios. Overall, this model, represented by Eq. (11), is essential for a comprehensive understanding of the operational dynamics of SCs in energy storage systems, enabling a detailed analysis of their charging and discharging behaviors, efficiency, and energy retention characteristics under various operational conditions.

subjected to the following constraints

12 $${E}_{SC}^{min}\le {E}_{SC}\left(t\right)\le {E}_{SC}^{max}$$

13 $$0\le {P}_{SC}\left(t\right)\le {P}_{SC}^{max}$$

Equations (12) and (13) define the safety and operational limits for SC. Equation (12) ensures that the energy level ${E}_{SC}\left(t\right)\le {E}_{SC}^{max}$ remains between a minimum ${E}_{SC}^{min}$​ and a maximum ${E}_{SC}^{max}$​, while Eq. (13) maintains the power ${P}_{SC}\left(t\right)$ within a maximum limit ${P}_{SC}^{max}$​. These constraints guarantee that the SC operates safely within its capacity.

### Inverter

In HRES, the role of converters and inverters is integral to maintaining the energy equilibrium among devices operating on AC and DC. Converters facilitate the transformation of AC into DC, whereas inverters serve the reverse function, turning DC back into AC^52^. Several design methodologies for these power conversion units have been documented in scholarly works. In this study, the converter’s design is predicated on the peak load approach, a technique detailed in the cited source^53^.

14 $$P_{{inv}} \left( t \right) = \frac{{P_{{peak}} }}{{\eta _{{inv}} }}$$

In Eq. (14), ${P}_{peak}$​ refers to the highest load demand, and ${\eta }_{inv}$​ represents the efficiency of the converter/inverter.

15 $${P}_{AC}={\eta }_{inv}{P}_{DC}$$

As detailed by Bao et al.^54^, Eq. (15) models the output power of an inverter. This formulation highlights the direct correlation between the inverter’s efficiency and its ability to convert DC into AC. It succinctly captures how the efficiency factor impacts the conversion process, resulting in the AC power output of the converter. The equation demonstrates the transformation of the DC input power into AC output power, emphasizing the significance of the converter’s efficiency in this process.

### Grid modeling

A grid constitutes an interconnected network of energy resources responsible for generating, accumulating, and storing energy. The energy sources from the grid is supplemented by the combined output of PV, WT, and BT components. These components are critical, especially in scenarios where their collective energy production does not meet the required electrical load demands. In cases of surplus energy generation, Eq. (16) is employed to calculate the revenue generated from selling this excess energy back to the grid^55^.

16 $${\mathrm{R}}_{\mathrm{grid}}={\sum }_{\mathrm{t}=1}^{8760}{\mathrm{r}}_{\mathrm{feed}\_\mathrm{in}}.{E}_{grid\_s}\left(t\right)$$

17 $${\mathrm{C}}_{\mathrm{grid}}={\mathrm{C}}_{\mathrm{p}}{\sum }_{\mathrm{t}=1}^{8760}{E}_{grid\_p}\left(t\right)$$

In this formulation, $${r}_{feed\_in}$$​ refers to the guaranteed feed-in tariff rate, which is specified as $0.01/kWh in this study, reflecting the actual value. The estimated cost of grid electricity procurement was calculated using Eq. (17). Here, $${C}_{p}$$​ denotes the estimated cost of purchasing 1 kW of electricity for the central campus, identified as the study site. This value, sourced from the university’s Department of Construction Works, has been established at $0.35/kWh, representing the current market rate.

18 $${\mathrm{P}}_{\mathrm{grid}}={\mathrm{P}}_{\mathrm{L}}\left(t\right)-{\sum }_{\mathrm{t}=1}^{8760}{\mathrm{P}}_{\mathrm{WT}}\left(t\right),{\mathrm{P}}_{PV}\left(t\right),{\mathrm{P}}_{B}\left(t\right),{\mathrm{P}}_{\mathrm{SC}}\left(t\right)$$

In Eq. (18), ${\mathrm{P}}_{\mathrm{grid}}$​ denotes the power supplied to or from the utility grid, reflecting the net flow of electricity. ${\mathrm{P}}_{\mathrm{L}}$​ represents the load demand, indicating the total power requirement. ${\mathrm{P}}_{B}$​ and ${\mathrm{P}}_{\mathrm{SC}}$​ signify the power supplied by the BESS and SC, respectively. The value of ${\mathrm{P}}_{\mathrm{grid}}$ varies based on the energy balance; it becomes negative during times of surplus generation (when energy production exceeds demand) and positive in situations of inadequate supply, highlighting the grid’s role in balancing energy needs.

### Economic parameters

In conducting financial assessments of integrated energy systems, it is essential to evaluate how key factors affect the project’s economic feasibility^56^. In this study, key financial indicators were evaluated, with a particular focus on the real interest rate, which was set at 2.07%, significantly impacting the financial analysis. The expected operational lifetime of these integrated energy systems is projected to be 20 years. Accurate cost data for the HRES components were obtained directly from the manufacturers for use in simulations. The economic and technical specifications of the microgrid components are provided in Appendix A.

## Objective function

The primary goal of this study is to establish an optimal configuration for the HRES at a university campus, aiming to effectively balance supply and demand while meeting load requirements at minimal costs^57^. To achieve this study evaluates four key decision variables: WT power, PV power, BT size, and SC size. The optimization of HRES sizing in this study employs the ACS as the main objective function for assessing the microgrid system^58^. ACS is used in economic analyses to identify the solution that offers the lowest annual cost while meeting all other requirements^59^. In this optimization problem, the primary decision variables are the capacities of the PV, WT, SC, and BT. These variables are critical in determining the configuration of the HRES^60^. The objective of the optimization is to minimize the ACS, while simultaneously considering system performance metrics such as the cost of energy (COE) and REF, which serve as outputs of the optimization rather than decision variables. The objective function is formulated to optimize these metrics based on the decision variables (PV, WT, SC, and BT), with the additional constraint that the REF must exceed 60% to ensure that the system meets environmental and sustainability targets. Additionally, NPC is included in the objective function, represented by Eq. (19), which accounts for the total lifecycle cost of the system, further emphasizing the importance of cost-efficiency in the design. Equation (20) integrates these elements into a multi-objective optimization framework. Therefore, the optimization seeks to balance the capacities of PV, WT, SC, and BT in such a way that the overall system ACS is minimized, while ensuring high renewable energy usage (REF > 60%) and competitive energy costs (COE). This approach ensures a comprehensive evaluation of system performance, reflecting both economic and environmental objectives.

19 $$Objective{ }Function = Min\left( {ACS,COE,NPC} \right)\;\;\;\left\{ {R_{WT} ,{ }R_{PV} ,{\mathrm{R}}_{{{\mathrm{SC}}}} ,{ }R_{BT} } \right\}$$

20 $$ACS=Min (Sola{r}_{cost}+Win{d}_{cost}+Inverte{r}_{cost}+Batter{y}_{cost}+{Supercapacitor}_{cost}+{Grid}_{cost})$$

### Cost analysis

This study conducts a detailed economic analysis of the cost factors associated with the various components of the proposed hybrid energy system, which integrates wind, solar, supercapacitors, batteries, and grid elements in a cohesive manner. The financial model used is comprehensive, encompassing key economic variables necessary for a complete assessment. These factors include the initial capital investment for system deployment, ongoing operational and maintenance costs, the expense of replacing components periodically over the system’s lifespan, salvage values at the end of its operational life, and the impact of real interest rates on the overall financial model^61^.

This in-depth approach to cost evaluation is critical for a precise understanding of the economic aspects of hybrid energy systems. It provides a detailed view of the financial requirements from the system’s inception to its decommissioning, offering a robust framework for estimating the total annual costs. Such detailed economic analysis is crucial for making well-informed decisions about the investment and operation of HRES, ensuring their long-term financial sustainability^62^. The capital cost for the battery component of the HRES is determined using the following calculation:

21 $$Batter{y}_{costC}={N}_{bat}\times \left[BA{T}_{C}\times \left(\frac{ir\times \left(1+ir\right)PR{J}_{LF}}{\left(1+ir\right)PR{J}_{LF}-1}\right)\right]$$

The cost associated with replacing the battery system is determined using the following Eq. (22)

22 $$Batter{y}_{cos{t}_{r}}={N}_{bat}\times BA{T}_{{C}_{r}}\times \left[\left(\frac{1}{{\left(1+ir\right)}^{Bat{e}_{LF}}}+\frac{1}{{\left(1+ir\right)}^{2\times Bat{e}_{LF}}}+\frac{1}{{\left(1+ir\right)}^{3\times Bat{e}_{LF}}}\right)\times \frac{ir\times \left(1+ir\right)PR{J}_{LF}}{\left(1+ir\right)PR{J}_{LF}-1}\right]$$

The salvage cost of the battery system is determined using the following calculation:

23 $$Bat{e}_{sa{l}_{f}r}=\left|4\times Bat{e}_{LF}-PR{J}_{LF}\right|$$

24 $$Batter{y}_{sa{l}_{cost}}={N}_{bat}\times BA{T}_{{C}_{r}}\times \left[\left(\frac{Bat{e}_{sa{l}_{f}r}}{Bat{e}_{LF}}\right)\times \frac{1}{{\left(1+ir\right)}^{PR{J}_{LF}}}\times \frac{ir\times \left(1+ir\right)PR{J}_{LF}}{\left(1+ir\right)PR{J}_{LF}-1}\right]$$

The overall cost of the battery system is computed using the following Eq. (25):

25 $$Batter{y}_{cost}=Batter{y}_{costC}+Batter{y}_{cos{t}_{r}}+\left({N}_{bat}\times Ba{t}_{OM}\right)-Batter{y}_{sa{l}_{cost}}$$

In a thorough evaluation of battery costs for a renewable energy system, Eqs. (21) through (25) address multiple critical factors. Equation (21) focuses on calculating the initial capital cost of the battery system, $Batter{y}_{costC}$, which takes into account the number of batteries (${N}_{bat}$), the unit cost of each battery ($BA{T}_{C}$), the interest rate ($ir$), and the project’s expected operational lifetime ($PR{J}_{LF}$). This equation essentially models the upfront financial investment, adjusted for interest and amortized over the system’s lifespan. Equation (22) shifts attention to the replacement cost of the batteries, denoted as $Batter{y}_{cos{t}_{r}}$. This formula incorporates the replacement unit cost per battery ($BA{T}_{{C}_{r}}$) and the battery lifespan ($Bat{e}_{LF}$), calculating how frequently the batteries need to be replaced during the course of the project.

In Eq. (23), the salvage value at the end of the project is determined by $Bat{e}_{sa{l}_{f}r}$, which estimates the remaining value of the batteries based on their lifespan and the overall project duration. This salvage value helps quantify the recoverable portion of the initial investment. Equation (24) introduces the salvage cost $Batter{y}_{sa{l}_{cost}}$, which is derived using the previously calculated salvage value and other system parameters. This cost reflects the monetary value that can be recuperated from the batteries when the project concludes. Finally, Eq. (25) aggregates all the relevant costs, including the capital expenditure, replacement costs, operation and maintenance expenses (${N}_{bat}\times Ba{t}_{OM}$), and salvage cost, to determine the total battery cost ($Batter{y}_{cost}$) for the system. This comprehensive approach ensures an accurate representation of the financial requirements throughout the entire lifecycle of the battery system within the renewable energy project.

The approach used to calculate the capital cost of a supercapacitor in an energy system is as follows:

26 $$S{C}_{costC}={N}_{SC}\times \left[S{C}_{C}\times \left(\frac{ir\times \left(1+ir\right)PR{J}_{LF}}{\left(1+ir\right)PR{J}_{LF}-1}\right)\right]$$

The cost associated with replacing the supercapacitor is determined using the following calculation:

27 $$S{C}_{cos{t}_{r}}=\frac{1}{{\left(1+ir\right)}^{S{C}_{LF}}}\times {N}_{SC}\times \left[S{C}_{{C}_{r}}\times \left(\frac{ir\times \left(1+ir\right)PR{J}_{LF}}{\left(1+ir\right)PR{J}_{LF}-1}\right)\right]$$

The maintenance cost of the supercapacitor is determined by the following Eq. (28):

28 $$S{C}_{O{M}_{c}ost}={N}_{SC}\times S{C}_{OM}$$

The overall cost of the supercapacitor is determined using the formula presented in Eq. (29).

29 $$S{C}_{tota{l}_{c}ost}=S{C}_{costC}+S{C}_{O{M}_{c}ost}+S{C}_{cos{t}_{r}}$$

Equations (26) to (29) evaluate the financial aspects of supercapacitors within an energy system, using previously defined parameters. Equation (26) estimates the initial capital investment ($S{C}_{costC}$), which depends on the number of supercapacitors (${N}_{SC}$) and the unit cost per supercapacitor ($S{C}_{C}$). Equation (27) calculates the replacement cost ($S{C}_{cos{t}_{r}}$​) by considering the replacement cost per unit and the expected lifespan of the supercapacitors ($S{C}_{LF}$). Equation (28) addresses ongoing maintenance expenses ($S{C}_{O{M}_{c}ost}$) by multiplying the number of supercapacitors by the per-unit maintenance cost ($S{C}_{OM}$). Finally, Eq. (29) combines the capital, replacement, and maintenance costs to determine the total supercapacitor cost ($S{C}_{tota{l}_{c}ost}$).

The method for calculating the capital cost of the wind energy component is outlined as follows:

30 $$Win{d}_{cos{t}_{c}}={N}_{w}\times \left[{C}_{wr}\times \left(\frac{ir\times \left(1+ir\right)PR{J}_{LF}}{\left(1+ir\right)PR{J}_{LF}-1}\right)\right]$$

The replacement cost for the wind energy component is determined using the formula presented in Eq. (31).

31 $$Win{d}_{cos{t}_{r}}={N}_{w}\times \frac{1}{{\left(1+ir\right)}^{Win{d}_{LF}}}\times \left[{C}_{wr}\times \left(\frac{ir\times \left(1+ir\right)PR{J}_{LF}}{\left(1+ir\right)PR{J}_{LF}-1}\right)\right]$$

The salvage cost for the wind energy component is calculated using the formulas presented in Eqs. (32) and (33).

32 $$Win{d}_{sa{l}_{f}r}=\left|Win{d}_{LF}-PR{J}_{LF}\right|$$

33 $$Win{d}_{sa{l}_{cost}}={N}_{w}\times \left[{C}_{wr}\times \frac{win{d}_{sa{l}_{f}r}}{Win{d}_{LF}}\times \frac{1}{{\left(1+ir\right)}^{PR{J}_{LF}}}\times \frac{ir\times \left(1+ir\right)PR{J}_{LF}}{\left(1+ir\right)PR{J}_{LF}-1}\right]$$

The yearly cost of the wind energy component is determined according to the formula outlined in Eq. (34).

34 $$\left[Win{d}_{cost}={N}_{w}\times {W}_{OM}+Win{d}_{cos{t}_{c}}+Win{d}_{cos{t}_{r}}-Win{d}_{sa{l}_{cost}}\right]$$

The cost evaluation of wind energy components, as outlined in Eqs. (30) to (34), involves several calculation steps. Equation (30) calculates the capital investment ($Win{d}_{cos{t}_{c}}$) by taking into account the number of wind turbines (${N}_{w}$) and the cost per unit (${C}_{wr}$). Equation (31) addresses the replacement costs ($Win{d}_{cos{t}_{r}}$) by considering the turbine count, the replacement cost per turbine, and the expected turbine lifespan ($Win{d}_{LF}$). Equation (32) determines the salvage value ($Win{d}_{sa{l}_{f}r}$) by using the difference between the turbine’s operational lifespan and the project’s lifespan ($PR{J}_{LF}$). Moving on, Eq. (33) estimates the salvage cost ($Win{d}_{sa{l}_{cost}}$), which depends on the turbine count, unit cost, and salvage factor. Finally, Eq. (34) calculates the total annualized cost of the wind turbine system ($Win{d}_{cost}$) by combining the capital, replacement, salvage, and operation and maintenance expenses. The method used to calculate the operational and maintenance costs for the solar energy component is as follows:

35 $$Sola{r}_{O{M}_{c}ost}={N}_{sol}\times {S}_{OM}$$

The capital cost for the solar energy component is determined using the following approach:

36 $$Sola{r}_{{C}_{c}ost}={N}_{sol}\times Cs\times \left(\frac{ir\times \left(1+ir\right)PR{J}_{LF}}{\left(1+ir\right)PR{J}_{LF}-1}\right)$$

The annualized cost for the solar energy component is determined using the following calculation:

37 $$Sola{r}_{cost}=Sola{r}_{O{M}_{c}ost}+Sola{r}_{{C}_{c}ost}$$

The converter’s capacity in the system is directly linked to the number of solar panels, with the converter size ($IN{V}_{size}$) matching the total count of installed solar panels (${N}_{sol}$). In the financial analysis of the solar energy component, Eq. (35) calculates the operation and maintenance expenses ($Sola{r}_{O{M}_{c}ost}$) by multiplying the total number of solar panels (${N}_{sol}$) by the per-unit operation and maintenance cost (${S}_{OM}$). Equation (36) determines the capital cost $Sola{r}_{{C}_{c}ost}$, considering the number of panels, the cost per panel ($Cs$), the interest rate ($ir$), and the projected lifespan of the project ($PR{J}_{LF}$), offering a comprehensive view of the initial investment. Lastly, Eq. (37) calculates the annualized cost of the solar energy system ($Sola{r}_{cost}$) by summing the operation and maintenance expenses and the capital cost^63^. The capital cost of the inverter is determined using the following calculation:

38 $$Inverte{r}_{C}=IN{V}_{size}\times \left(IN{V}_{C}\times \frac{ir\times \left(1+ir\right)PR{J}_{LF}}{\left(1+ir\right)PR{J}_{LF}-1}\right)$$

The replacement cost of the inverter is determined using the formula provided in Eq. (39).

39 $$Inverte{r}_{Cr}=IN{V}_{size}\times \left[IN{V}_{Cr}\times \left(\frac{1}{{\left(1+ir\right)}^{IN{V}_{LF}}}\right)\times \left(\frac{ir\times \left(1+ir\right)PR{J}_{LF}}{\left(1+ir\right)PR{J}_{LF}-1}\right)\right]$$

The operation and maintenance costs of the inverter are calculated using the formula presented in Eq. (40).

40 $$Inverte{r}_{OM}=IN{V}_{size}\times IN{V}_{OM}$$

The salvage fraction of the inverter is determined using the formula provided in Eq. (41).

41 $$IN{V}_{sa{l}_{f}r}=\left|IN{V}_{LF}-PR{J}_{LF}\right|$$

The salvage cost of the inverter is determined using the formula presented in Eq. (42).

42 $$Inverte{r}_{sa{l}_{C}}=IN{V}_{size}\times \left(IN{V}_{C}\times \frac{IN{V}_{sa{l}_{f}r}}{IN{V}_{LF}}\times \frac{1}{{\left(1+ir\right)}^{PR{J}_{LF}}}\times \frac{ir\times \left(1+ir\right)PR{J}_{LF}}{\left(1+ir\right)PR{J}_{LF}-1}\right)$$

The total cost of the inverter is determined using the formula provided in Eq. (43).

43 $$Inverte{r}_{Tcost}=Inverte{r}_{C}+Inverte{r}_{Cr}+Inverte{r}_{OM}-Inverte{r}_{sa{l}_{C}}$$

In the financial evaluation of the energy system, the inverter costs are analyzed through Eqs. (38) to (43). Equation (38) computes the initial capital cost ($Inverte{r}_{C}$) based on the inverter size ($IN{V}_{size}$) and cost per unit ($IN{V}_{C}$). Equation (39) addresses the replacement cost ($IN{V}_{Cr}$), factoring in the inverter size, unit replacement cost, and lifespan ($IN{V}_{LF}$). For operational expenses, Eq. (40) calculates the operation and maintenance cost ($Inverte{r}_{OM}$) by multiplying the inverter size by the per-unit maintenance cost. The salvage fraction of the inverter, calculated in Eq. (41), is used in Eq. (42) to determine the inverter’s salvage cost ($Inverte{r}_{sa{l}_{C}}$). Finally, the total inverter cost ($Inverte{r}_{Tcost}$) is computed in Eq. (43) by aggregating the capital cost, replacement cost, and operation and maintenance costs, while subtracting the salvage cost. This approach provides a comprehensive financial overview of the inverter component over the lifecycle of the project^64^. The cost of grid-purchased energy is determined using the formula provided in Eq. (44).

44 $$Gri{d}_{cost}=\sum \left({E}_{gri{d}_{p}}\right)\times 0.35-\sum \left({E}_{gri{d}_{s}}\right)\times 0.01$$

Equation (44), labeled as $Gri{d}_{cost}$, provides the framework for calculating the net cost of energy transactions involving the grid in an energy system. This equation accounts for two primary aspects of grid interaction: the energy purchased from the grid and the energy sold back to the grid. The total grid cost is computed by multiplying the amount of energy imported from the grid (${E}_{gri{d}_{p}}$) by a unit cost of 0.35, and subtracting the value of energy exported to the grid (${E}_{gri{d}_{s}}$), where each unit is credited at a rate of 0.01. This formulation captures both the financial outlay for purchasing energy and the revenue generated from exporting surplus energy. Additionally, it highlights the asymmetry in pricing, where the cost of buying energy from the grid is generally higher than the compensation for selling energy back to the grid.

### Renewable energy fraction (REF)

In this study, the emphasis is on optimizing the REF within a grid-connected HRES. The REF value serves as a crucial metric, reflecting the degree to which the system uses RESs^65^. Pertinently, for systems connected to the grid, Eq. (45) has been employed to quantify the percentage of energy drawn from the grid compared to the total load demand^66^:

45 $$REF\left(\mathrm{\%}\right)=\left(1-\frac{{\sum }_{t=1}^{8760}{E}_{grid\_p}}{{\sum }_{t=1}^{8760}Total Load}\right)100$$

This equation quantifies the contribution of RESs to the total energy demand in percentage terms. During the optimization process, if the REF value falls below a certain threshold ${\varepsilon }_{REF}$, penalties are imposed on the annual cost to eliminate constraints (Annual cost = 10^9^)^67^, as indicated in Eq. (46):

46 $$REF\left(\mathrm{\%}\right)\le {\varepsilon }_{REF}$$

Equation (31) ensures that the REF value exceeds a specific limit during the optimization process, thus supporting the sustainable and efficient operation of the system. This approach aims to find the most cost-effective and efficient solution while increasing the share of renewable sources in HRES’s energy production. Minimizing the second part of this equation is essential to maximize REF. This function has an upper limit and cannot exceed 100%. This limitation signifies the need to keep the function below a certain value during the optimization process.

### Design variables

In this study, the design variables, also referred to as decision variables, encompassed the WT power, PV power, BT power, and SC power. The upper and lower limits of these variables are delineated in Eq. (47).

47 $$\text{Decision Variables}=\left\{\begin{array}{c}1 kW\le {\mathrm{R}}_{\mathrm{WT}}\le 10000 kW\\ 1 kW\le {\mathrm{R}}_{\mathrm{PV}}\le 10000 kW\\ 1 kW\le {\mathrm{R}}_{\mathrm{SC}}\le 800 kW\\ 1 kW\le {\mathrm{R}}_{\mathrm{BT}}\le 1000 kW\end{array}\right.$$

In this context, ${R}_{WT}$ denotes the power of WT, ${R}_{PV}$ represents the power of PV panels, ${R}_{BT}$ indicates the power of BT, and ${\mathrm{R}}_{\mathrm{SC}}$ refers to the power of SC. Establishing the upper and lower bounds of these decision variables is a process that is intricately dependent on the specific problem at hand. To facilitate optimal convergence of the solutions within the algorithms, the bounds of these variables are determined through a systematic trial-and-error method. This approach leads to the selection of values that yield the most advantageous outcomes.

## Methodology

Optimization entails the strategic design or configuration of a system with the goal of securing optimal outcomes. The central objective in optimization typically involves either maximizing or minimizing a specific goal, which frequently centers on aspects such as cost, performance, or efficiency. Within the scope of the HRES sizing optimization problem, various constraints can hinder the maximization or minimization of these objectives^68^. These constraints may stem from diverse factors, including design parameters, material choices, physical space limitations, production capabilities, budget, and temporal considerations. In optimization processes, these constraints serve to impose defined limitations on the system or procedure under enhancement. This study incorporates distinct constraints, including a minimum renewable energy fraction (REF) (at least 60%) and a maximum annual cost of system (Annual cost = 10^9^). These stipulations guide the optimization algorithm in modifying the design parameters to achieve a solution compliant with these constraints. The power generation and energy consumption profiles of the HRES are pivotal in determining the system’s power output. The design of an HRES is inherently tied to these profiles because the power derived from the HRES depends on meteorological inputs. Consequently, the dimensioning of the hybrid components is influenced by meteorological parameters, as shown in Eq. (1). This section expounds on the steps involved in the employed optimization algorithm, detailing the sizing methodology for the proposed HRES, strategies for energy management, and formulation of the objective function.

The simulations were conducted using MATLAB R2022a environment, implementing custom-coded meta-heuristic optimization algorithms. The simulation was based on a real-world dataset consisting of 8760 hourly values of solar radiation, wind speed, ambient temperature, and electrical load, collected for the region of Yalova, Türkiye. The optimization framework considers four decision variables representing the rated power of photovoltaic panels, wind turbines, battery storage, and supercapacitors. The objective function focuses on minimizing the annualized cost of the system. Each optimization run was executed with a population size of 30 agents and a maximum of 100 iterations. Algorithm-specific solver mechanics were retained in each implementation to preserve algorithm integrity and performance characteristics. This setup allows for a fair and reproducible comparison across all selected algorithms.

### HRES sizing and optimization

**Fig. 5:**
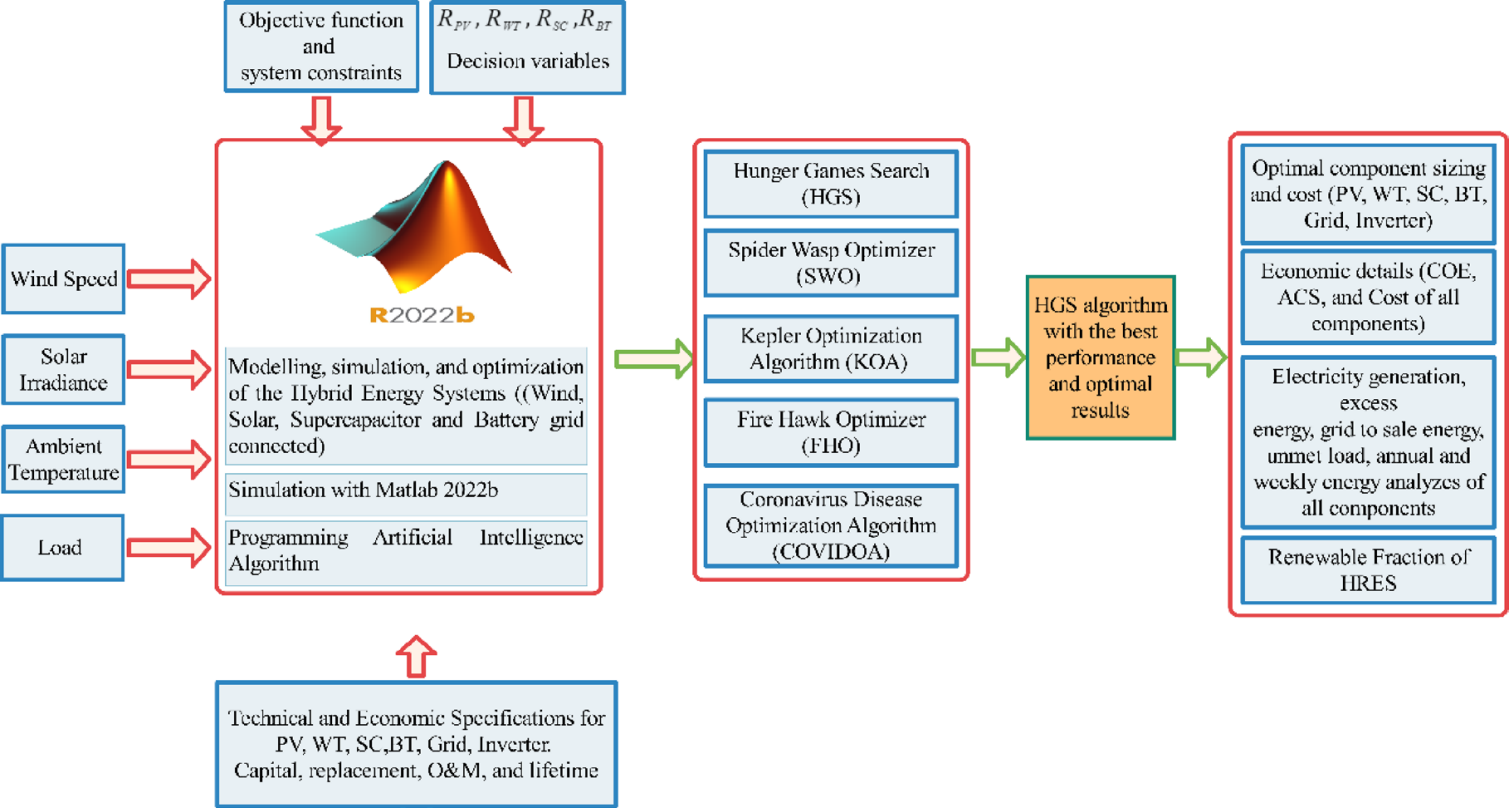
System sizing methodology.

The sizing and optimization process of the HRES in this study was conducted using various methods based on the selected parameters^69^. Energy optimization models serve as tools for developing energy strategies and summarizing the potential future structure of a system under specific conditions. This approach facilitates gaining insights into technological advancements, structural changes, and policy recommendations^70^. MATLAB software was used to identify the most suitable configurations for the chosen location and to expedite the problem-solving process. To initiate computations, MATLAB requires inputs such as hourly climate data (ambient air temperature, solar irradiation, and wind speed), the load profile for the selected scenario, and the technical and economic characteristics of the HRES components^71^. The inputs and outputs of the system and the algorithms used in the sizing process are depicted in Fig. 5.

The accompanying visual presents a comprehensive narrative of the HRES sizing and optimization process. The models are based on the R2022b software for simulation and optimization, which encompasses a broad spectrum of artificial intelligence optimization algorithms. These algorithms include KOA, HGS, COVIDOA, FHO, and SWO, among which the HGS algorithm demonstrates the best performance and optimal results. The data obtained from the optimization process include ideal component sizing and costs (PV, WT, SC, BT, Grid, Inverter), economic details (COE, ACS, and costs of all components), electricity generation, surplus energy, unmet load, annual and weekly energy analyses, and the REF of the HRES. The technical and economic characteristics of these components encompass critical factors such as capital and replacement costs, operation and maintenance (O&M) costs, and lifespan, serving as fundamental elements in assessing the system’s overall performance.

Figure 5 illustrates the critical aspects of the HRES sizing and optimization process, providing guidance on managing this process and the preferred objective functions. This section offers insights into the selection of algorithms used and their applications in optimizing HRES.

### Energy management system

Managing power in systems reliant on RESs poses significant challenges because of their inherent unpredictability^72^. This makes it particularly complex to ensure a consistent energy supply that can adapt to fluctuating load demands over different periods. The simulation study addresses this issue by considering various scenarios for applying optimization strategies:

#### Case 1: Achieving balance between generation, load, and BT charging

This scenario entails renewable energy sources successfully generating enough power to meet load demands. Surplus energy is strategically directed to charge the BT. The primary goal is to maintain a balance between energy production, load consumption, and efficient BT charging.

#### Case 2: Allocating excess energy to SCs

As with the first scenario, renewable sources produce more energy than required. However, excess energy is stored in SCs instead of BTs. This case focuses on optimizing energy distribution to exploit the rapid charge and discharge capabilities of SCs.

#### Case 3: Addressing renewable generation shortfalls with BT discharge

When renewable sources are unable to meet the load demands, this scenario involves using the stored energy in BTs to cover the deficit. The optimization here is centered on efficiently managing the BT discharge to bridge the gap between supply and demand.

#### Case 4: Supercapacitor intervention for energy shortfalls

In situations where both renewable generation and BT storage prove insufficient, SCs are employed to provide the necessary support. The optimization strategy in this case is to effectively utilize the supercapacitor’s quick response capabilities to overcome energy shortages.

#### Case 5: Resorting to grid power in severe energy deficiencies

This scenario unfolds under extreme conditions where renewable sources, BTs, and SCs are inadequate. Energy is then procured from the grid. Optimization efforts are directed toward minimizing grid energy dependence while ensuring system stability in these critical situations.

**Fig. 6:**
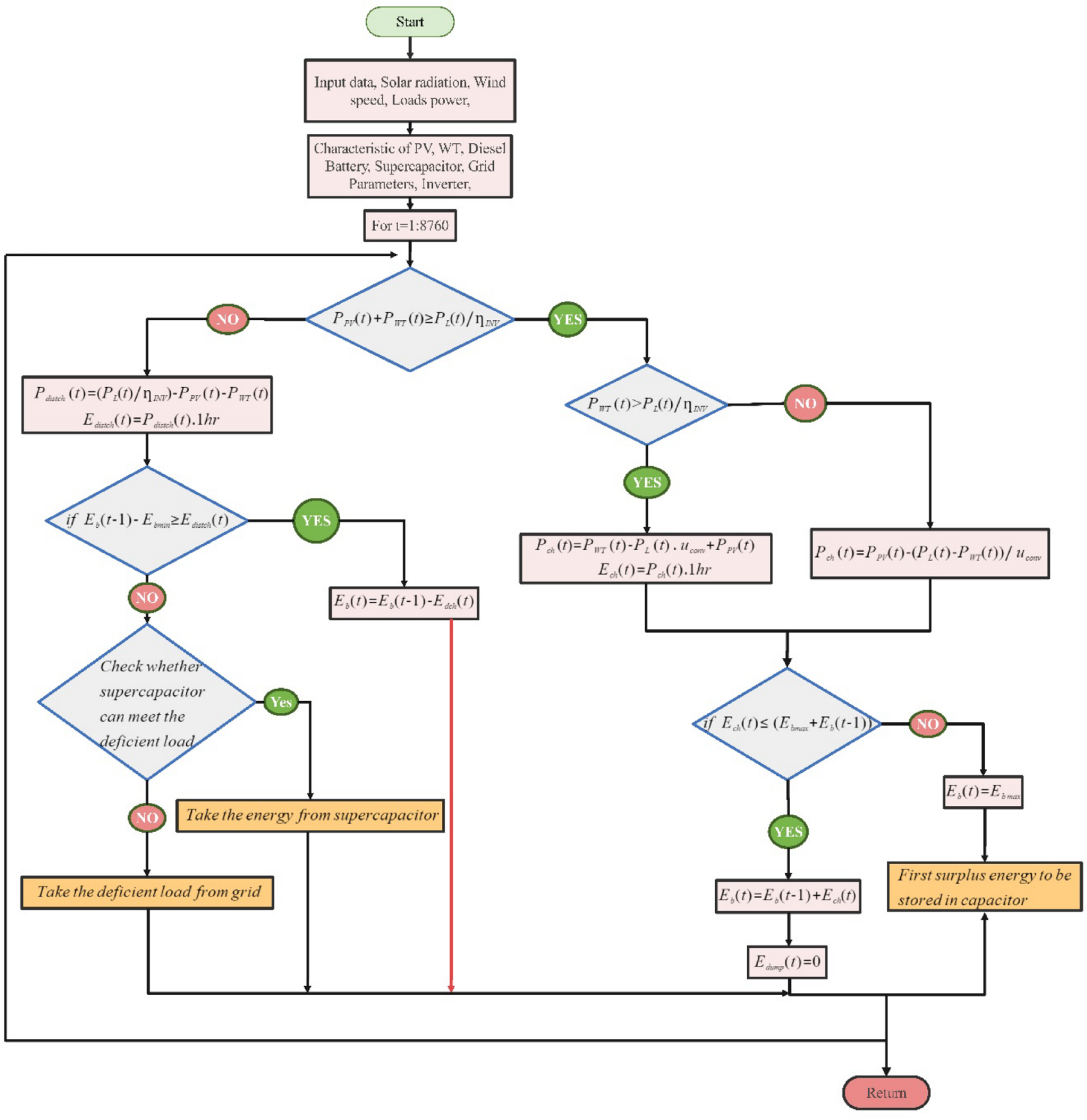
Power management of the on-grid PV/WT/BT/SC hybrid energy system.

These scenarios collectively form a comprehensive framework for understanding and developing effective power management strategies in HRES, offering insights into optimizing energy usage across varying conditions and ensuring a stable power supply. Figure 6 presents a schematic representation of the flow algorithm for the selected HRES model. It succinctly portrays the intricate decision-making process, highlighting the interactions among various components based on real-time energy dynamics.

### Hunger games search (HGS) algorithm

This section presents the specifics of the HGS algorithm, including its mathematical framework.

#### Search strategies, behavioral choices, and hunger-driven dynamics in animals

Animals base their actions and decisions on computational rules in response to their environment, which are crucial for their cognitive development and survival strategies. Hunger, as a primary motivator, significantly influences their behaviors and choices, driving them to prioritize food search in times of need. This necessitates a balance between various behaviors, including exploration and defense, demonstrating adaptability in feeding approaches^73^. Behavioral choices in animals are driven by a blend of factors influenced by immediate needs and environmental stimuli^74^. Hunger is a dominant motivator, overriding other drives and shaping behaviors toward achieving stability^75^. Social behavior aids in survival, with communication playing a key role in resource location and predator avoidance. The evolutionary process favors those who are better at finding food, impacting survival rates and potentially leading to evolutionary outcomes. Hunger, defined as prolonged absence of food, intensifies the urgency of finding nourishment before life-threatening consequences occur^76^. In this context, animals engage in strategic behaviors to locate food, and their actions are driven by logical decision-making processes^77^.

#### Mathematical model and foraging strategy of the HGS method

This section elaborates on the HGS method and its mathematical model, which is derived from hunger-based activities and behavioral choices, demonstrating simplicity yet effective performance. In the natural world, social animals typically collaborate in food foraging; however, not all individuals participate in this cooperative behavior^78^. The HGS algorithm’s individual cooperative communication and foraging behaviors are encapsulated in the game-theoretic rules presented in Eq. (48).

48 $$\overrightarrow {{X\left( {t + 1} \right)}} = \left\{ {\begin{array}{*{20}c} {Game_{1} :\overrightarrow {X\left( t \right)} .\left( {1 + rand\left( 1 \right)} \right),} & {r_{1} < l} \\ {Game_{2} :\overrightarrow {{W_{1} }} .\overrightarrow {{X_{b} }} + \vec{R}.\overrightarrow {{W_{2} }} .\left| {\overrightarrow {{X_{b} }} - \overrightarrow {X\left( t \right)} } \right|,} & {r_{1} > l, r_{2} > E} \\ {Game_{3} :\overrightarrow {{W_{1} }} .\overrightarrow {{X_{b} }} - \vec{R}.\overrightarrow {{W_{2} }} .\left| {\overrightarrow {{X_{b} }} - \overrightarrow {X\left( t \right)} } \right|,} & {r_{1} > l, r_{2} < E} \\ \end{array} } \right.$$

In this section, the mathematical intricacies of the HGS algorithm was discussed. The vector $\overrightarrow{R}$ operates within the range [ − a, a]; the elements ${r}_{1}$ and ${r}_{2}$ are randomly selected numbers between 0 and 1; $rand\left(1\right)$ denotes a number generated from a normal distribution; and $t$ symbolizes the ongoing iteration. The vectors $\overrightarrow{{W}_{1}}$ and $\overrightarrow{{W}_{2}}$ represent hunger-driven weights, a concept developed based on hunger-induced signaling behaviors^79^. The notation $\overrightarrow{{X}_{b}}$, is used for the optimal individual’s position in the current iteration, whereas $\overrightarrow{X(t)}$ signifies the positions of all individuals. The l value, a parameter to be elaborated upon in a parameter-setting experiment, is crafted to refine the algorithm. The term $\overrightarrow{X(t)}.\left(1+rand\left(1\right)\right)$ depicts an agent’s random, hunger-driven search for food from its existing position. The difference $\left|\overrightarrow{{X}_{b}}-\overrightarrow{X(t)}\right|$ models the range of activity for an individual at a given moment and, when multiplied by $\overrightarrow{{W}_{2}}$, assesses hunger’s influence on this range. Vector $\overrightarrow{R}$ is incorporated as a regulatory tool to confine the activity range, gradually reducing toward zero, reflecting the cessation of the search as hunger diminishes. The interaction of $\overrightarrow{{W}_{1}}.\overrightarrow{{X}_{b}}$ manipulates the range of activity, simulating a scenario in which an individual is informed by peers upon reaching the source of food and then resumes foraging at the current position after feeding. Here, $\overrightarrow{{W}_{1}}$ captures the error in accurately perceiving the real position. Equation (49) details the formula $E$, which ensures controlled variation across all positions.

49 $$E=sech\left(\left|F\left(i\right)-BF\right|\right)$$

In this model, each participant is identified as $i$, which varies from 1 to $n$. The function $F(i)$ is used to evaluate the fitness level of each individual. It determines the effectiveness or adaptability of each participant within the algorithm. The optimum fitness value achieved in the current cycle is indicated by $BF$. In addition, the model incorporates the hyperbolic function Sech, which is mathematically defined by the equation $\mathrm{sech}\left(x\right)=2/{(e}^{x}+{e}^{-x})$, highlighting an essential component of the model’s calculation methodology.

The vector $\overrightarrow{R}$ is formulated by Eq. (50):

50 $$\overrightarrow{R}=2\times \mathrm{shrink}\times \mathrm{rand}-\mathrm{shrink}$$

In this context, the shrink factor is computed using Eq. (51):

51 $$\mathrm{shrink}=2\times \left(1-\frac{t}{T}\right)$$

where ‘rand’ symbolizes a random number within the [0, 1] interval, and T signifies the total number of iterations.

**Fig. 7:**
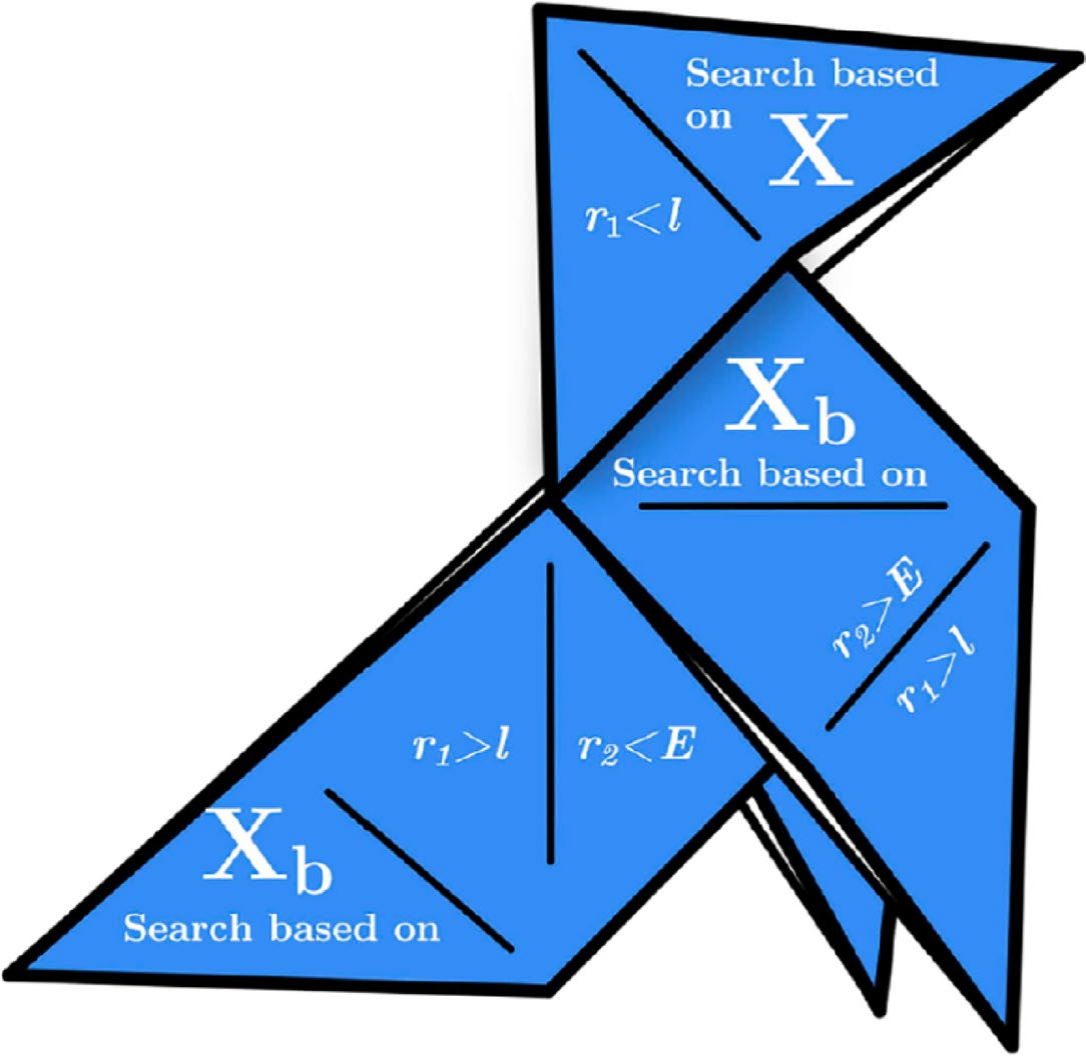
Illustration of the HGS algorithm’s operational logic during the optimization process.

Figure 7 depicts the search process and logic of the HGS in voids, as governed by the rule in Eq. (48). From the graph, it is evident that the directions of the search can be categorized into two distinct types based on the nature of the source points:

**Independent Search based on **$\overrightarrow{{\boldsymbol{X}}}$**:** The initial game rule replicates an autonomous approach, characterized by a lack of collaborative effort and sole focus on an eager quest for food.

Collaborative Search based on $\overrightarrow{{{\boldsymbol{X}}}_{{\boldsymbol{b}}}}$→: The second rule of the game closely integrates the variables $\overrightarrow{R}$, $\overrightarrow{{W}_{1}}$, and $\overrightarrow{{W}_{2}}$. Adjusting these parameters allows the evolution of an individual’s position within the feature space, emulating the collaborative efforts of multiple entities in their search for nourishment.

Equation (34) establish guidelines that enable individuals to investigate areas both close to and distant from the optimal solution. This ensures comprehensive search within the solution space limits to a certain degree (diversification), a principle that is equally applicable in high-dimensional search spaces.

#### Influence of Hunger

This section mathematically models how hunger or starvation impacts the search behavior of individuals.

The expression for $\overrightarrow{{W}_{1}}$ in Eq. (52) is outlined as follows:

52 $$\overrightarrow{{W}_{1}(i)}=\left\{\begin{array}{c}hungry\left(i\right).\frac{N}{SHungry}\times {r}_{4}, {r}_{3}<l\\ 1{r}_{3}>l\end{array}\right.$$

The expression for $\overrightarrow{{W}_{2}}$ in Eq. (53) is outlined as follows:

53 $$\overrightarrow{{W}_{2}(i)}=\left(1-exp\left(-\left|hungry\left(i\right)-SHungry\right|\right)\right)\times {r}_{5}\times 2$$

In this context, ‘hungry’ denotes each individual’s level of hunger; $N$ stands for the total number of individuals; $SHungry$ is the collective hunger sensation of all individuals, calculated as sum($hungry$); ${r}_{3}$, ${r}_{4}$, and ${r}_{5}$ represent random values within the [0, 1] range. For the precise computation of $hungry(i),$ refer to the formulation presented in Eq. (54).

54 $$hungry\left(i\right)=\left\{\begin{array}{c}0, AllFitness\left(i\right)==BF\\ hungry\left(i\right)+H, AllFitness(i)!=BF\end{array}\right.$$

In this model, $AllFitness(i)$ maintains the fitness level of each individual during the current iteration. At each iteration stage, the fittest individual’s hunger level is assigned a value of 0. For the rest, a revised hunger value $(H)$ is computed, building upon their initial hunger levels. This results in varying $H$ values across individuals. The formula for $H$ is presented in Eq. (55).

55 $$TH=\frac{F\left(i\right)-BF}{WF-BF}\times {r}_{6}\times 2\times \left(UB-LB\right)$$

56 $$H=\left\{\begin{array}{c}LH\times \left(1+r\right), TH<LH\\ TH, TH\ge LH\end{array}\right.$$

In this framework, ${r}_{6}$ is identified as a random value within the [0, 1] range. $F(i)$ denotes the fitness level of each individual, with $BF$ representing the highest fitness achieved in the current iteration. Conversely, $WF$ indicates the lowest fitness level obtained in the same iteration. The feature space is defined by $UB$ and $LB$, which represent its upper and lower limits, respectively. The hunger sensation, labeled as $H$, is restricted to a minimum limit, $LH$^80^. This constraint is essential for enhancing the algorithm’s performance. By managing hunger’s upper and lower thresholds and discussing $LH$’s value during parameter setting experiments, the algorithm’s efficiency is optimized. Hunger influences the activity range both positively and negatively, thus $\overrightarrow{{W}_{1}}$ and $\overrightarrow{{W}_{2}}$ are modeled to simulate this effect. Equation (55) employs the difference between $UB$ and $LB$ to signify the maximum hunger experienced by an individual in varying environments. The term $F(i)-BF$ quantifies the amount of food required to satisfy hunger. In each iteration, the hunger metric is subject to change.

**Fig. 8:**
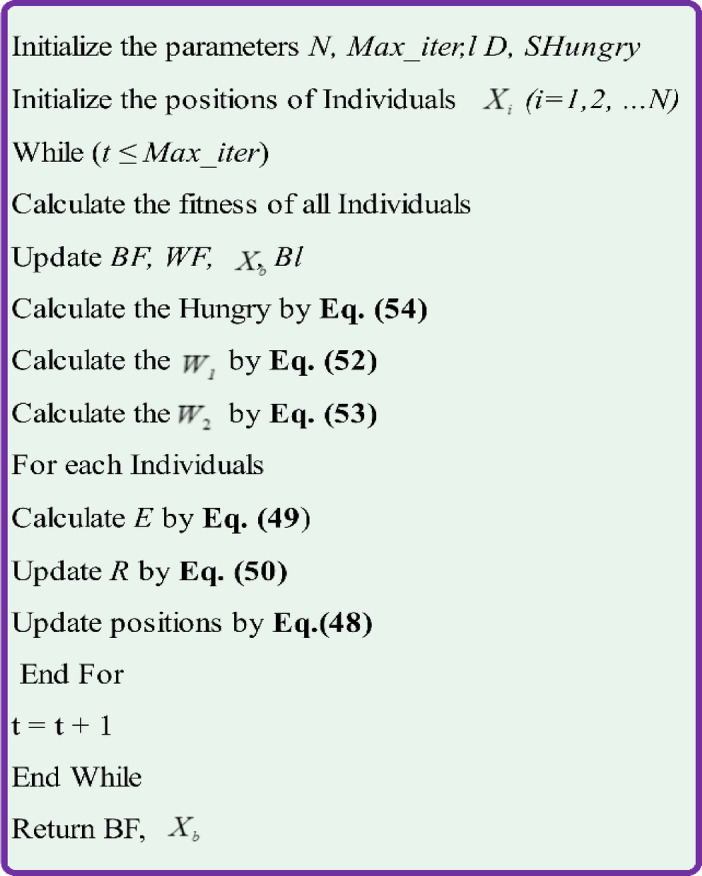
Pseudecode of the HGS algorithm.

**Fig. 9:**
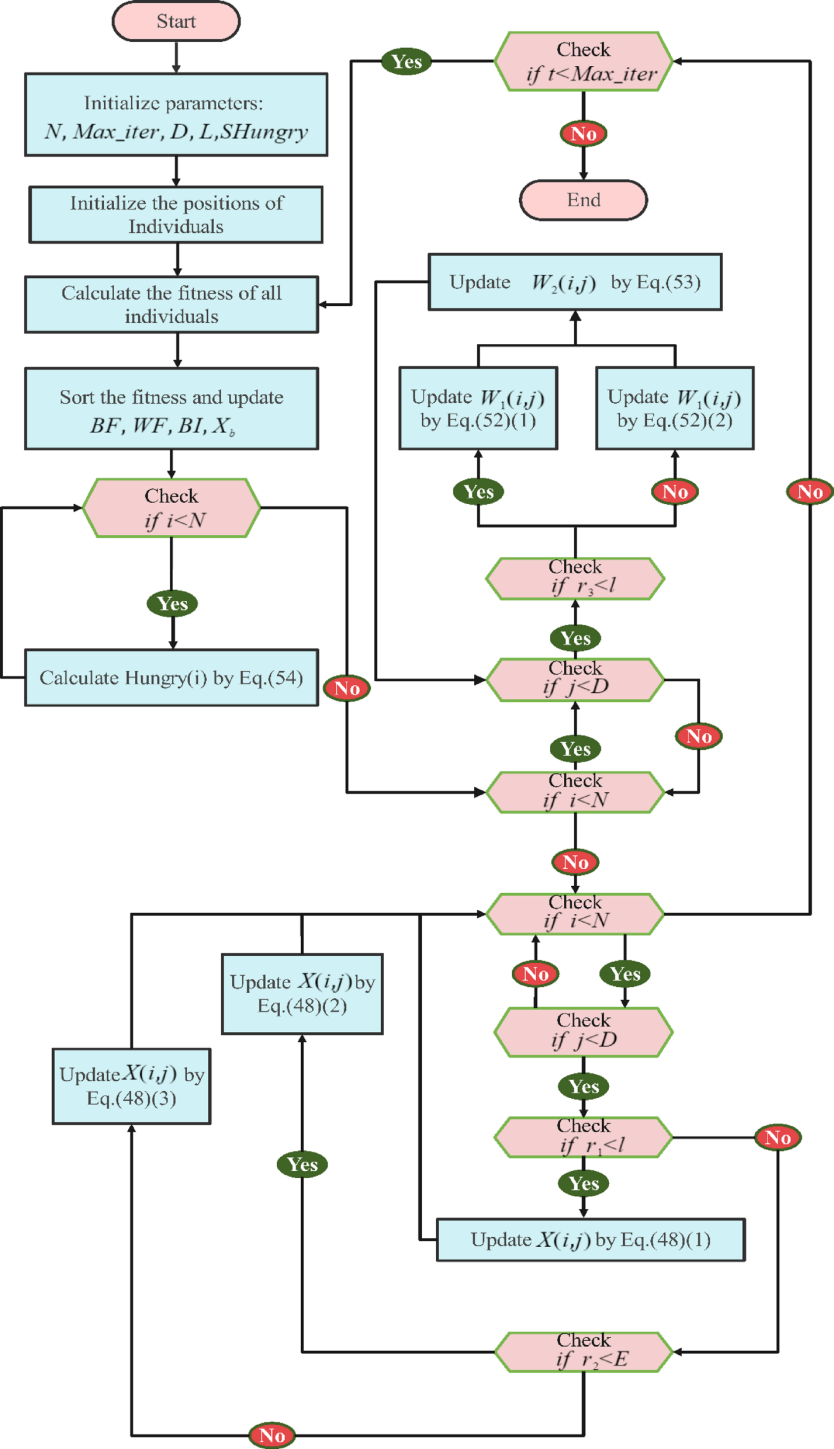
Flowchart of the HGS algorithm.

The expression $WF-DF$ calculates the total foraging capacity of an individual in the current iteration, with $F(i)-DF/WF-DF$ denoting the hunger ratio. This factor ${r}_{6}\times 2$ is used to gauge environmental influences, either positive or negative, on hunger. The HGS algorithm, although proficient in emulating the basic behaviors of social animals, offers potential for further advancement. This includes adaptations to mirror the behaviors of living organisms more closely, incorporating their unique traits, and integrating additional mechanisms. The primary goal is to streamline the algorithm for maximum scalability. Figure 8 details the pseudocode for the proposed Hunger Games Search, and the process flow is shown in Fig. 9.

The HGS algorithm, as a gradient-free and population-based approach, enhances its capability to tackle complex problems by blending stochastic elements. This method stands out in dealing with multiple challenges and local optima through its adaptive mechanisms. Considering the hunger rate and its impact on activity, HGS becomes flexible to adapt to fitness-focused changes. By using individuals’ past performance data to guide behavioral modifications, the HGS evolves initial positions and search strategies through parameters l and E, thereby expanding the algorithm’s solution space. Hunger weights, $\overrightarrow{{W}_{1}}$ and $\overrightarrow{{W}_{2}}$​​, increase fluctuations during the search, preventing the algorithm from falling into local optima traps. The $\overrightarrow{R}$ parameter, by reducing the search step to a certain extent, facilitates a comprehensive examination and in-depth exploration of the targeted solution space. Focusing on the best $\left({X}_{b}\right)$ and standard $(X)$ solutions, HGS enables a more extensive exploration of the feature space and the uncovering of hidden areas. Despite its simple structure, HGS offers superior performance and effective results compared with existing methods. In this study, when compared with the KOA, COVIDOA, FHO, and SWO algorithms used in HRES dimension optimization, the HGS algorithm yielded the most optimal result. Table 2 lists the parameters of the optimization algorithms used.

**Table 2:** Comparative optimal sizing using HGS, KOA, COVIDOA, FHO, and SWO techniques.

Optimization results	KOA	HGS	COVIDOA	FHO	SWO
WT power (kW)	659.8704	663.6215	680.4933	652.3685	847.3762
PV power(kW)	578.3146	588.9159	612.6553	571.9247	386.1530
SC size(kW)	668.3352	672.7371	800.0000	785.7572	344.3198
BT size(kWh)	1000.0000	1000.0000	1000.0000	980.5446	676.8045
Total WT energy (kWh)	2,063,607.0009	2,075,337.9306	2,128,100.8340	2,040,146.3788	2,649,992.2320
Total PV energy (kWh)	1,134,372.6242	1,155,167.2293	1,201,732.3152	1,121,838.6699	757,444.7889
Total grid energy generation (kWh)	511,914.0337	505,999.2794	472,907.7973	504,780.9998	590,937.1745
Grid sale energy(kWh)	941,518.1511	965,982.3916	1,028,482.4744	924,397.1763	1,154,449.5256
SC output Energy(kWh)	115,588.1169	116,750.7221	135,457.5933	131,940.2845	78,906.1155
Load demand(kWh)	2,535,675.9619	2,535,675.9619	2,535,675.9619	2,535,675.9619	2,535,675.9619
SC charge energy (kWh)	127,857.7152	129,149.3810	149,962.3820	146,007.4311	87,358.8491
SC discharge energy (kWh )	115,588.1169	116,750.7221	135,457.5933	131,940.2845	78,906.1155
BT charge energy (kWh)	265,664.9530	266,247.2677	268,291.6771	261,025.5259	209,755.8119
BT discharge energy (kWh)	255,011.8755	255,589.0930	257,556.2603	250,670.7518	202,069.4610
COE $(\mathrm{\$}/\mathrm{kWh})$	0.23803	0.23801	0.23834	0.238605	0.24970
REF (%)	79.8115	80.0447	81.3498	80.0928	76.6950
WT fraction (%)	44.3947	44.4586	45.5252	44.5627	50.831
PV fraction (%)	24.4039	24.7464	25.7079	24.5042	14.5289
**ACS **$(\boldsymbol{\$}$**)**	**603,588.8287**	**603,538.4357**	**604,372.8664**	**605,026.5648**	**633,183.6344**
WT cost ($\$$)	191,460.2845	192,548.6735	197,443.9857	189,283.6213	245,864.7729
PV cost ($\$$)	48,499.6921	49,388.7580	51,379.6314	47,963.8074	32,384.2786
BT cost ($\$$)	22,070.5663	22,070.5663	22,070.5663	21,641.1758	14,937.4592
SC total cost ($\$$)	48,327.7325	48,646.0361	57,848.4886	56,818.5891	24,897.9792
Inverter Tcost ($\$$)	46,688.7176	47,544.5858	49,461.1202	46,172.8428	31,175.0523
Grid cost ($\$$)	246,541.8353	243,339.8157	226,169.0739	243,146.5281	283,924.0919

During the optimization process using algorithms such as HGS, KOA, COVIDOA, FHO, and SWO, the standard practice involves configuring the population size to 30 and limiting the number of iterations to 100.

## Simulation results and discussion

This study was conducted using data from Yalova University’s Central Campus in Turkey. It proposed an efficient approach for sizing a HRES consisting of PV/WT/BT/SC components, leveraging the energy exchange between the microgrid and the demand load on the university’s central campus. Simulations were conducted in MATLAB over a period of 8760 h in 2022, using hourly sampling, and various metaheuristic algorithms such as HGS, KOA, COVIDOA, FHO, and SWO were employed. The optimization process considered numerous constraints, including RE, REF, SOC, and optimal system sizing. Sensitivity analysis was performed in the simulation to determine the impact of various values on system performance. The results of each algorithm used in the optimization process are presented in Table 2. The reported values are derived from high-precision numerical outputs of the MATLAB optimization model. However, in practical implementations, these values would be rounded to the nearest commercially available system sizes to ensure feasibility while maintaining minimal deviation from the optimized design.

The energy costs identified in this study are comparable to those reported in previous studies. A different study conducted by Güven et al. for a similar location, which was based on the techno-economic analysis of standalone FC, BG, WT, and PV systems, determined the ideal system configuration using the HFGA algorithm, with an ACS value of $2,921,702.3^34^. The metaheuristic algorithms KOA, COVIDOA, FHO, and SWO were programmed and used for the microgrid system design problem to validate the effectiveness and reliability of the proposed HGS in determining the best sizing results for grid-connected HRES. The obtained results were compared with those from the proposed HGS, and the convergence characteristics of the algorithms were evaluated. Across iterations, there was a decrease in the ACS values, indicating that the optimization algorithm was progressing toward the optimum system size by reducing the components of the objective function. Therefore, any reduction in the objective function was significant because it provided more insight into the optimal size. A comprehensive analysis of the convergence processes of the algorithms for the optimal design of the hybrid microgrid system revealed that HGS completed the optimization process more rapidly, producing better outcomes while reducing computation time and resource consumption. As presented in Table 2, HGS offered the best solution compared to other algorithms, with superior results of 588.9159 kW for PV, 663.6215 kW for WT, 672.7371 for SC, 1000.0000 kWh for BT, and an ACS value of $603,538.4357. The REF rate was determined to be 80.0447%. Additionally, 505,999.2794 kWh of energy was purchased from the grid throughout the year, and a total of 965,982.3916 kWh of energy was sold. These findings suggest that both HGS and other optimization-based algorithms can be successfully employed to determine the best solutions for challenging microgrid design problems. However, among these five algorithms, HGS provided faster and more optimal results.

All price details include the total cost, ongoing maintenance and operation expenses, project lifespan, and initial quantities and costs of each component within the hybrid power system. The designs for the HGS, KOA, COVIDOA, FHO, and SWO methods, presented in Table 2, were developed individually. These designs were then evaluated to ensure that they provide highly accurate solutions within the shortest possible timeframe.

**Fig. 10:**
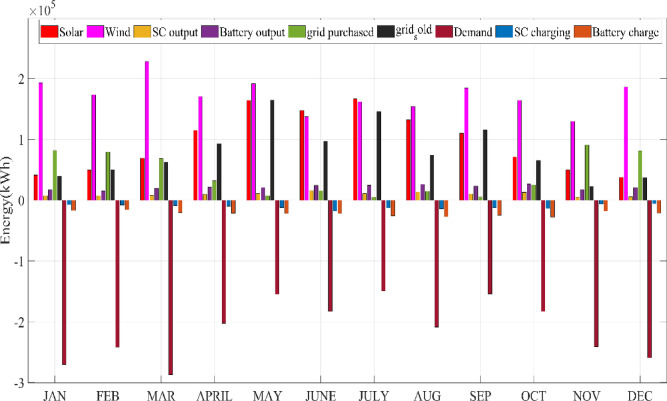
Monthly energy production, storage, and grid interaction over a year (HGS algorithm).

Figure 10 illustrates the monthly energy balance throughout the year, detailing contributions from various sources and storage methods, and interactions with the energy grid. This figure includes data on solar and wind energy generation, SC and BT outputs, energy transactions with the grid, and overall energy demand. Solar energy generation is noticeably lower in January and February, which is expected because of the shorter days of winter. Wind energy, on the other hand, shows less seasonal variation, maintaining relatively stable output throughout the year with a modest increase during the spring months, potentially attributable to prevailing seasonal winds. The SC output remains relatively stable year-round, indicating a consistent discharge profile. Similarly, the BT output demonstrates consistency, reinforcing the reliability of these storage systems in the energy mix. Based on the analysis of the graphs (Figs. 4, 10), the increase in energy demand is primarily observed in the months of March, January, December, and November. In contrast, during the summer months of June, July, and August, energy demand is relatively lower compared to other periods, likely due to the reduction in consumption during the vacation season. The interaction with the grid reveals a dependency on external energy sources during the winter and early spring months, highlighted by the increased energy purchases during this period. Conversely, energy sold back to the grid in certain months, particularly in late spring and summer, suggests periods when generation exceeded local demand, resulting in a surplus. Charging activities for both SCs and BTs align with the availability of solar energy, indicating the strategic use of storage systems to capture and effectively utilize renewable energy. In summary, the energy system’s reliance on the grid during low production periods is balanced by its ability to provide surplus energy back to the grid when renewable generation is abundant. The data advocates for an optimized approach to energy storage and an increase in renewable energy capacity to enhance grid independence and better address the peaks in demand observed during the summer months. This study presents the results of such optimization efforts, demonstrating realized enhancements in system performance.

**Fig. 11:**
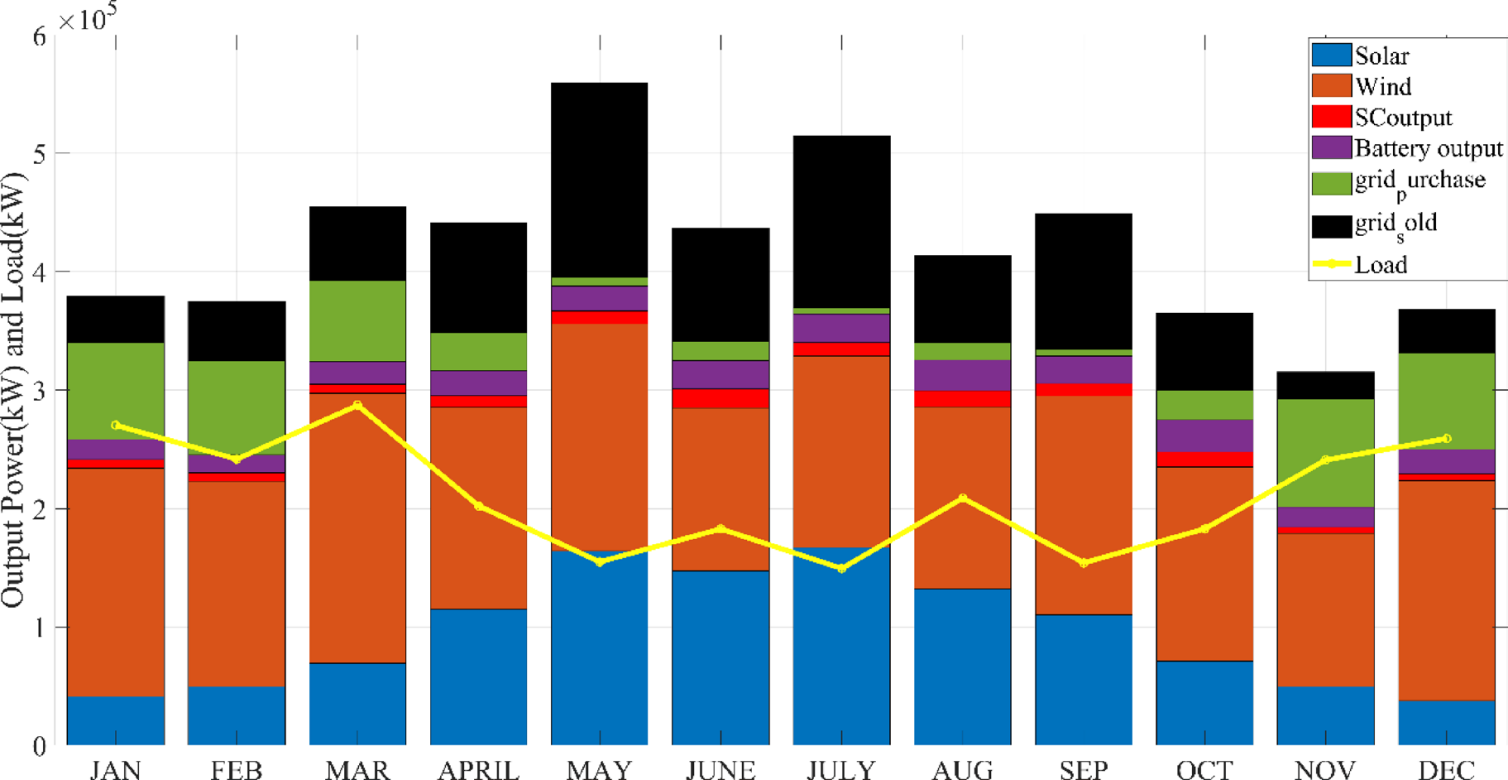
Average monthly energy output and load profile (HGS algorithm).

In Fig. 11, the juxtaposition of the stacked bars and the overlaying line graph illustrates the monthly alignment of energy outputs from solar, wind, and storage systems with the dynamic profile of energy demand. Notably, the contributions of solar and wind energy reveal a complementary interplay, which, along with strategic energy storage and grid interactions, forms the backbone of meeting fluctuating load requirements throughout the year.

**Fig. 12:**
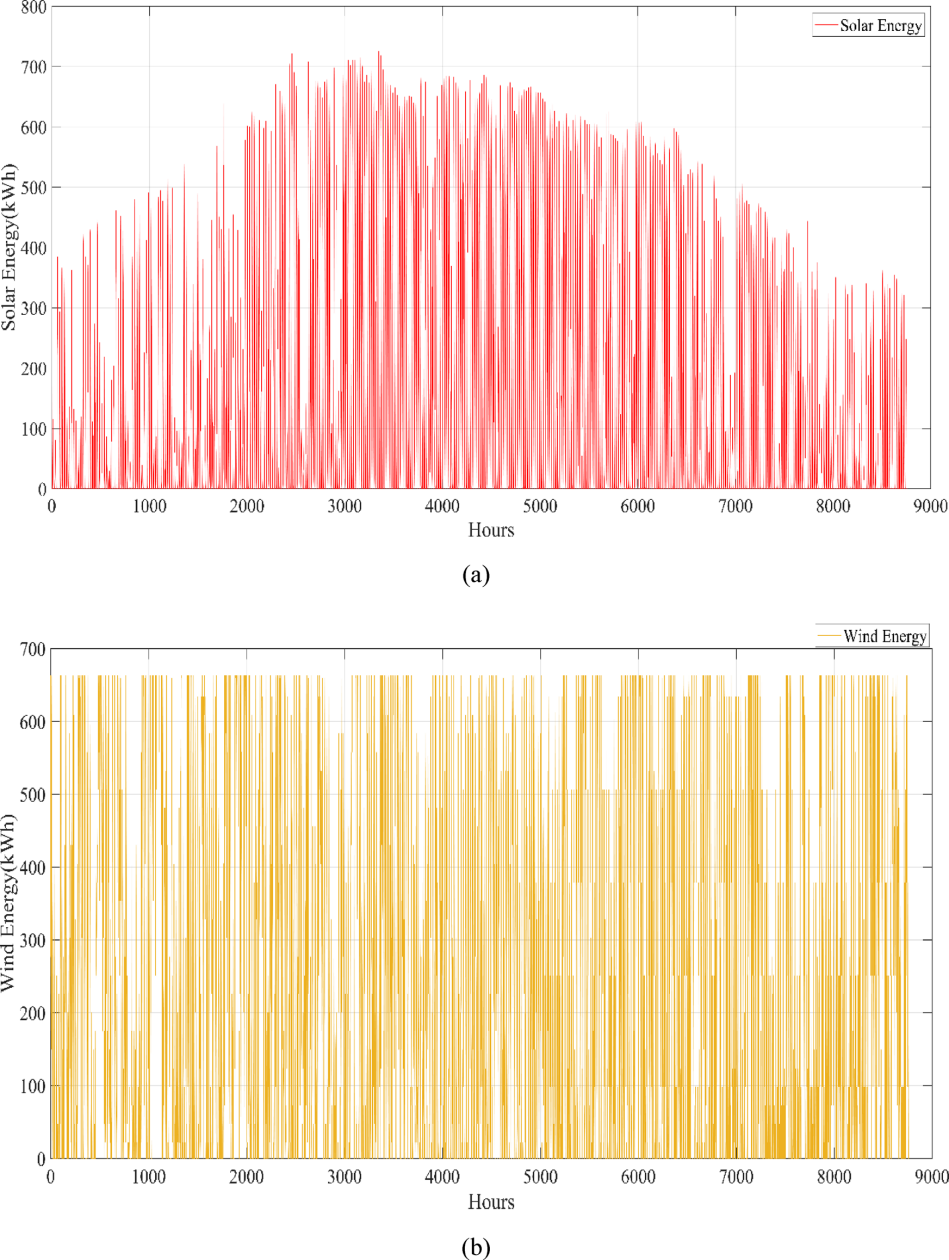
Average hourly energy (**a**) solar (**b**) wind (HGS algorithm).

Figure 12a illustrates the average hourly energy yield of PV systems derived from sunlight, while Fig. 12b displays the hourly energy production of WT systems. These graphs, determined for the optimal system configuration, reflect the energy production values at each hour t during a monitoring period of 8760 h in detail. Compared with the high energy production peaks of PV systems during daylight hours, the more balanced and consistent energy production profile of WT systems plays a supportive role in maintaining system stability, especially during hours of low sunlight or at night. These data are critically important for the integration of RESs and the development of energy management strategies. When considered in conjunction with energy storage solutions, this study demonstrates how the overall efficiency of the system can be optimized against daily and seasonal demand fluctuations.

**Fig. 13:**
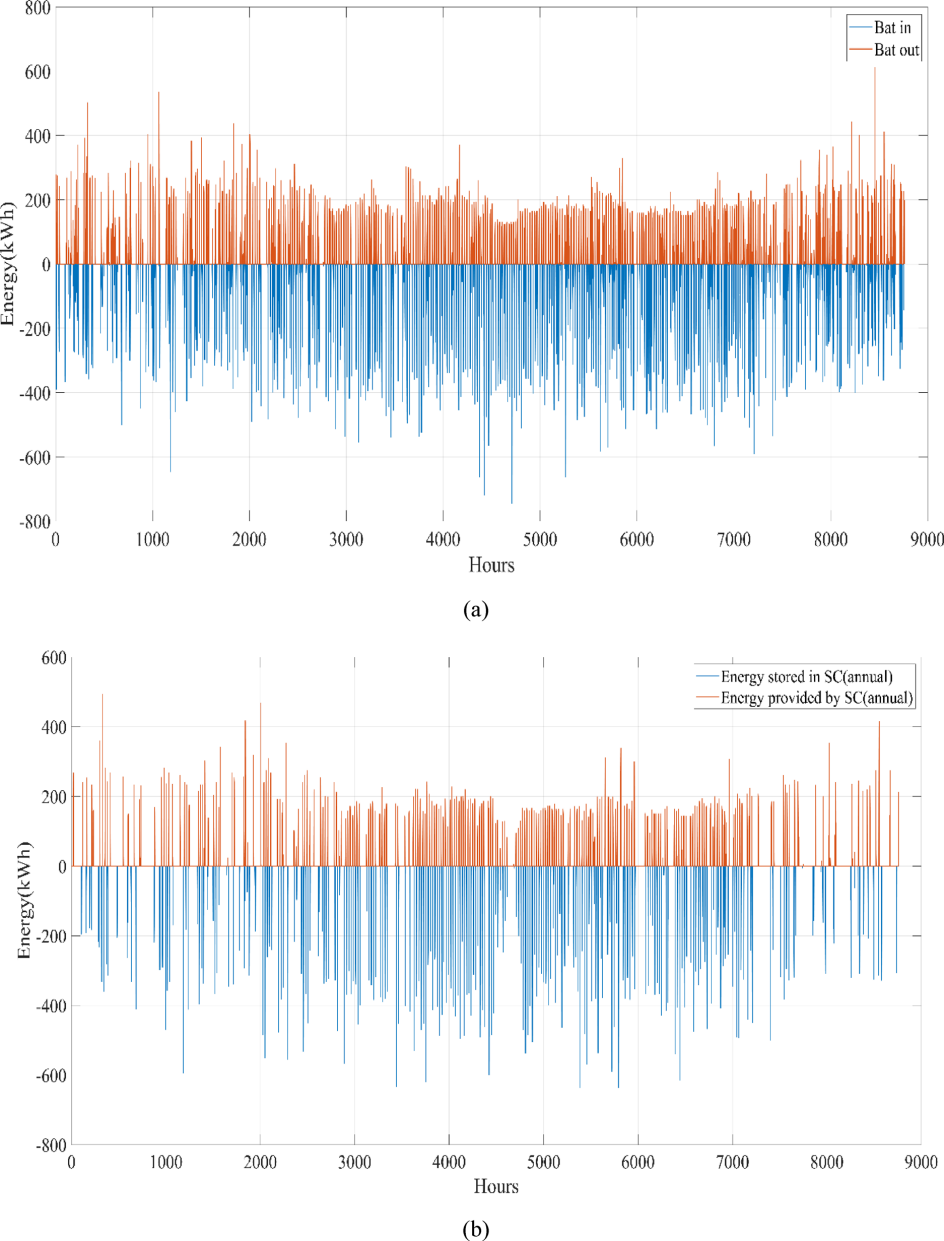

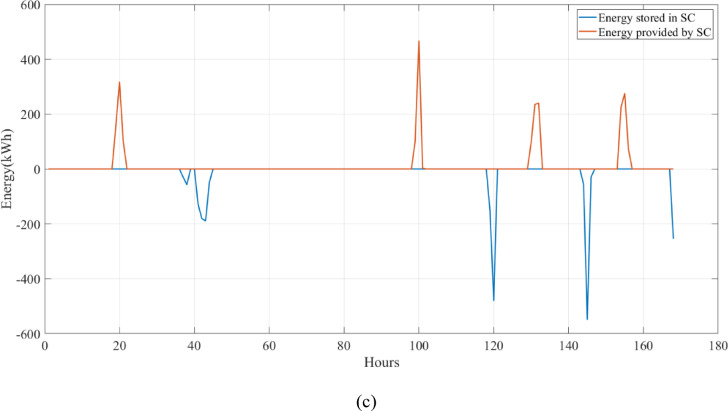
Input and output energy values ​​on an hourly basis a) BT b) SC c)weekly SC (1907-2074 h) (HGS algorithm).

Figure 13a shows the energy storage dynamics of the HRES. Here, the ‘Bat in’ (blue line) and ‘Bat out’ (orange line) curves display the annual energy charging and discharging profile of the BT storage unit in our system. These curves illustrate how our system manages the constant fluctuations between energy production and consumption, and how the BT storage unit supports energy supply security by storing generated energy and providing it on demand.

Figure 13b details the role of SC in energy management. The 'Energy stored in SC (annual)' (blue line) represents the amount of energy stored in SC, while the 'Energy provided by SC (annual)' (orange line) reflects the amount of energy supplied by the SCs. In Fig. 13c, the capabilities of SC energy storage and supply are demonstrated through short-term fluctuations during the period with the highest load demand (1907–2074 h). 'Energy stored in SC’ (blue line) shows how much energy is stored in SC, and 'Energy provided by SC’ (orange line) indicates when and how much of this stored energy is made available for use. The high charge/discharge rates and energy densities of SCs enable a rapid response to sudden fluctuations in energy production and consumption, which is critically important for maintaining the stability of the energy system, especially during peak hours or sudden demand surges. Both graphs demonstrate how our hybrid energy system can effectively manage the natural variability of RESs using energy storage and recovery technologies. These data play a significant role in enhancing the overall efficiency and reliability of the system and optimizing energy production.

**Fig. 14:**
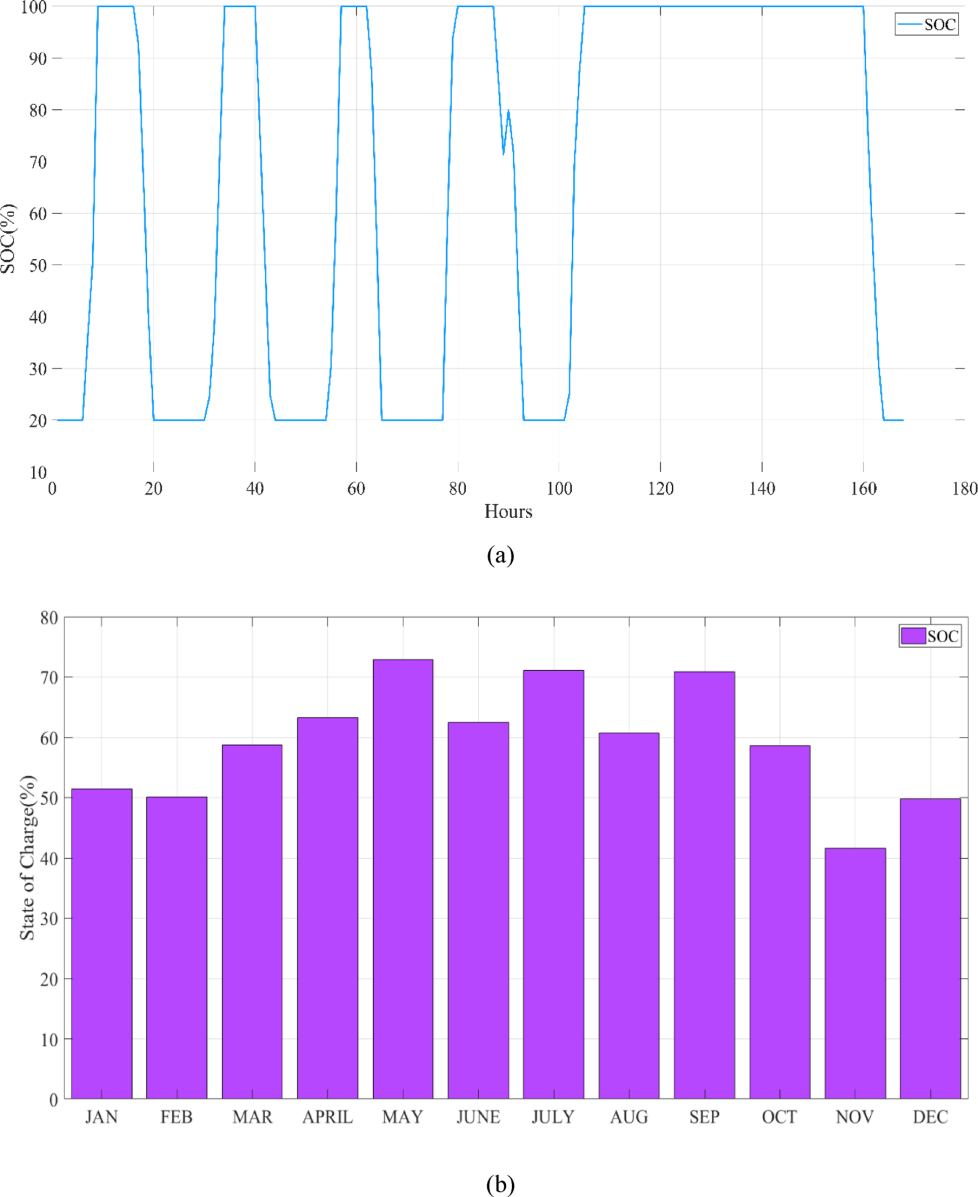
(**a**) Hourly variation of SOC (4543–4710 h), (**b**) Average monthly variation of SOC (HGS algorithm).

SOC is a critical parameter for HRES. Its importance stems from the fact that the operational capacity and efficiency of the BT components used for energy storage in HRES are directly correlated with this value. SOC represents the current energy capacity of a BT as a percentage and provides essential insights into how much energy HRES can generate or store. Figure 14a displays the SOC values for the week of the year with the lowest load demand (between 4543 and 4710 h) for BT, while Fig. 14b depicts the monthly average SOC values throughout the year. These figures are fundamental in determining the periods when the system operates optimally in accordance with its energy requirements and storage capacity or when energy demand escalates.

Figure 14b presents the yearly variation in the average SOC for BT within the HRES. Charging levels for the BT were recorded at 51.38% in January, decreasing slightly to 50.05% in February, rising to 58.63% in March, reaching 63.30% in April, peaking at 72.88% in May, then moderating to 62.52% in June, increasing again to 71.13% in July, declining to 60.06% in August, elevating to 70.87% in September, reverting to 58.63% in October, dropping to 41.52% in November, and settling at 49.68% in December. Continuous monitoring of the charging and discharging activities of BT is essential in an HRES. Keeping the SOC within optimal ranges is fundamental to guarantee a consistent energy supply and to enhance the service life and performance of BT.

**Fig. 15:**
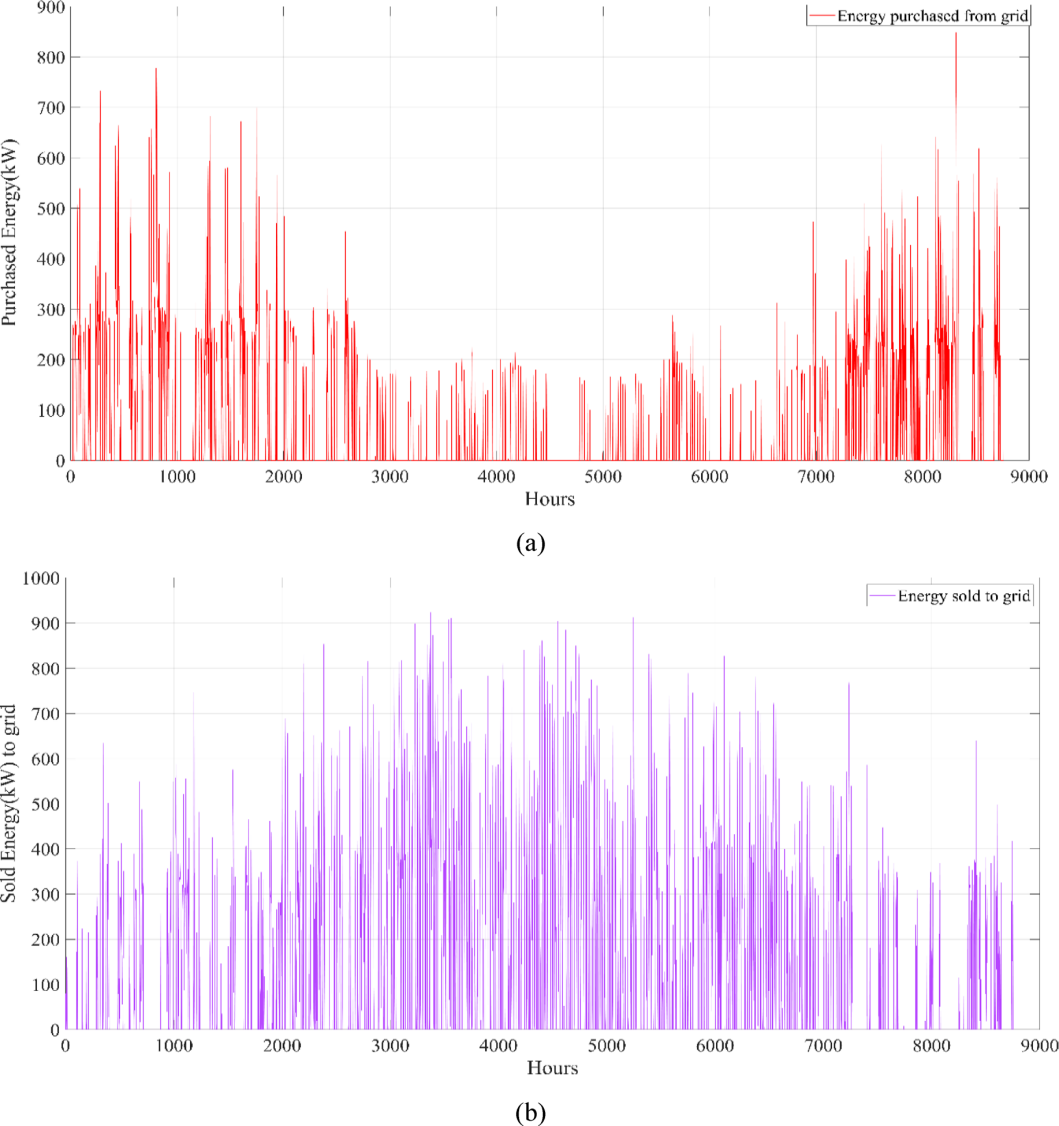
Comparison of (**a**) energy purchased from the grid, and (**b**) energy sold to the grid (HGS algorithm).

Figure 15(a) displays the energy purchased from the grid throughout the year, and Fig. 15(b) shows a graph of the energy sold to the grid. These figures are presented to provide information on how much energy is bought and sold hourly throughout the 8760-h period. As a result, it was determined that a total of 965,982.3916 kWh of energy was sold to the grid, and 505,999.2794 kWh was purchased from the grid over the course of the year.

**Fig. 16:**
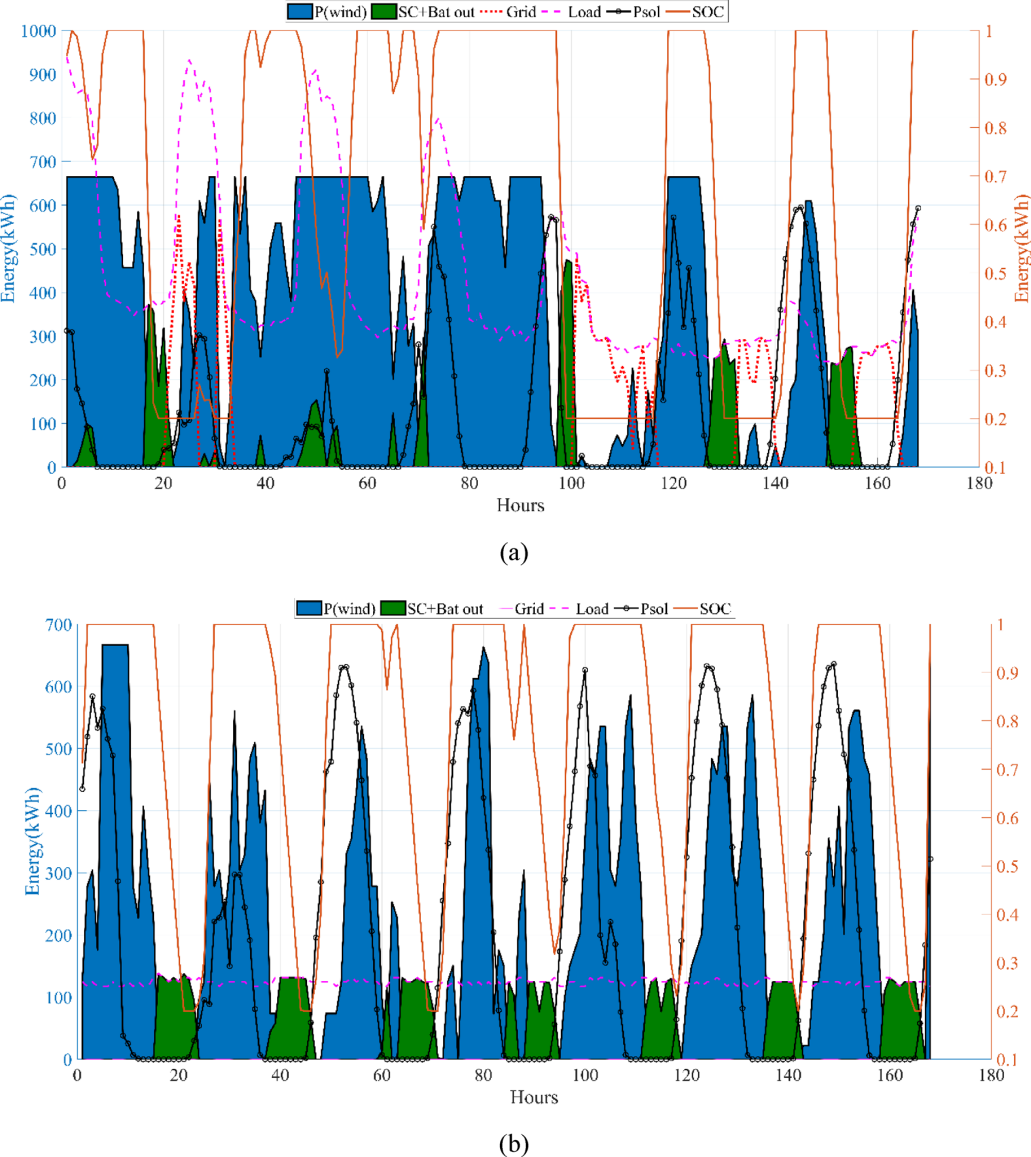
Comparative weekly energy profiles for HRES: (**a**) period of maximum load demand (1907–2074 h) and (**b**) period of minimum load demand (4543–4710 h) (HGS algorithm).

Figure 16 presents two distinct energy profiles of an HRES under varying load demands, with part (a) corresponding to the week with the highest energy demand and part (b) corresponding to the week with the lowest. In Fig. 16a, the energy contribution from wind power, indicated by the blue bars, shows high variability, with peaks suggesting strong wind conditions during this high-demand period. This variability underscores the importance of wind power in the energy mix, particularly its potential to provide substantial energy during peak times. However, the intermittent nature of wind energy necessitates effective storage solutions and grid support, as shown by the green segments, which signify the usage of energy from storage ('SC + Bat out’). The red dashed line, representing the energy interaction with the grid, highlights the grid’s supplementary role during times when renewable generation and storage do not fully meet the demand. The orange line, which represents the State of Charge (SOC), shows the battery’s charge levels throughout the period.

Figure 16b reflects a period of lower demand, during which the wind energy profile (blue bars) is noticeably reduced. The 'SC + Bat out’ energy discharges (green segments) are less frequent and lower in magnitude, indicating a reduced reliance on stored energy. Although Fig. 16b includes a pale dashed line intended to represent grid interaction, there is no visible energy exchange with the grid during this period, reflecting the system’s self-sufficiency. The orange line continues to track the SOC, showing how much energy is stored in the system.

The juxtaposition of these profiles illustrates the dynamic flexibility of the HRES to adapt to different load demands. During the high-demand period, the system effectively utilizes both renewable generation and storage to meet the load, whereas during low demand, the system’s ability to feed energy back into the grid demonstrates its potential for grid support and economic benefit through energy sales. These insights are critical for optimizing the operation and economic planning of HRES, indicating the system’s capability to not only meet but also contribute to the energy ecosystem.

**Fig. 17:**
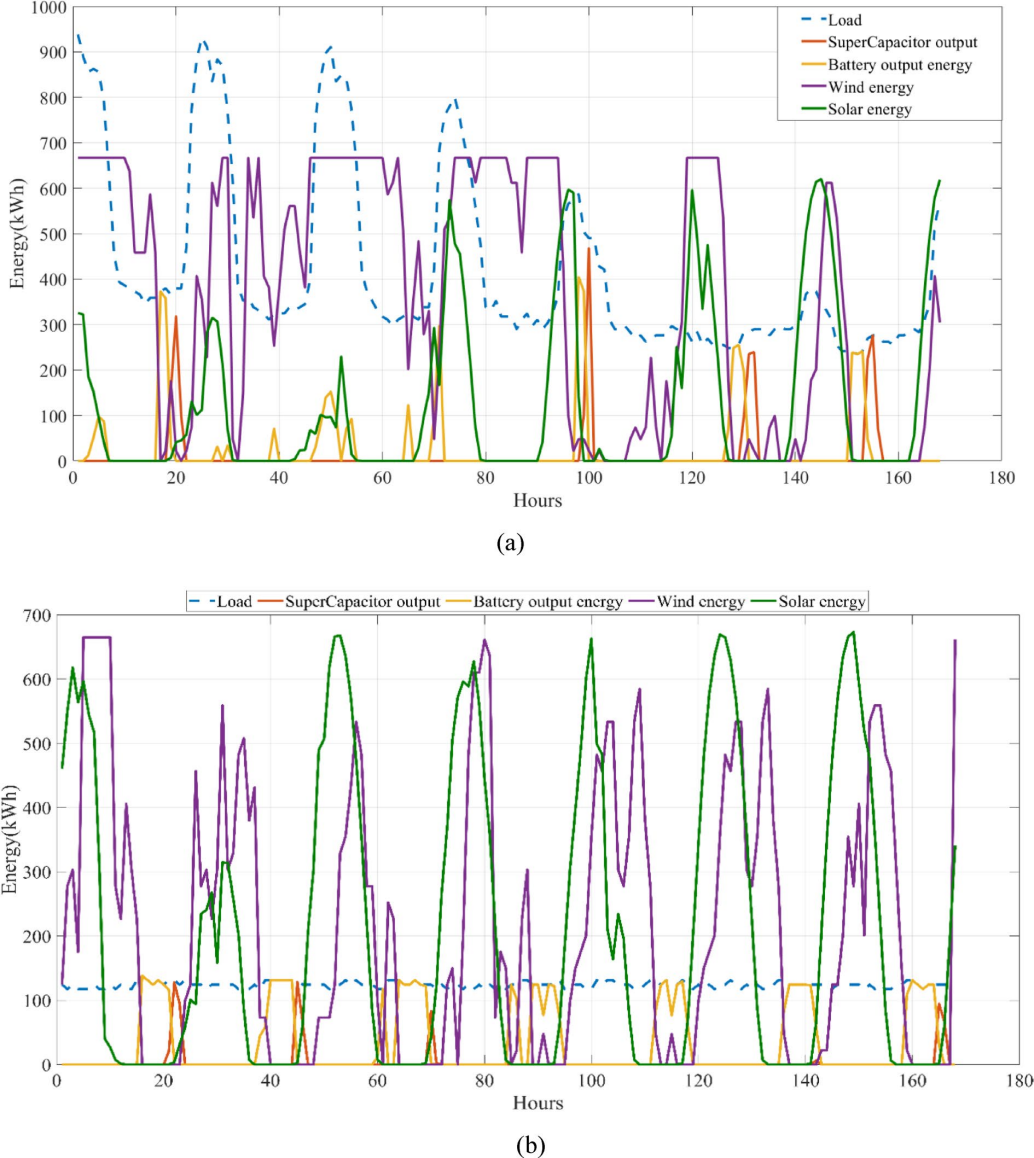
Differential analysis of HRES energy use across weekly cycles: (**a**) interval experiencing the highest demand (1907–2074 h), (**b**) interval experiencing the lowest demand (4543–4710 h) (HGS algorithm).

In Fig. 17a, the storage outputs from both SC (orange line) and BT (yellow line), closely follow the energy load profile (blue dashed line). This suggests that the storage systems are effectively discharged during times of high demand. The contributions of wind (purple line) and solar (green line) fluctuate, reflecting the inherent variability of renewable sources. The activity of the storage components during peak load times indicates their role in bridging the intermittency of renewables. On the other hand, Fig. 17b displays a starkly different scenario with the reduced load, where the energy contributions from storage systems are minimized, showing less frequent discharge occurrences. The solar energy output, which is particularly high during daylight hours, indicates a potential surplus when compared with the lower load requirements, implying the possibility of energy being redirected to the grid or reserved for future use. These figures illustrate HRES’s capability to modulate energy generation and storage to effectively meet varying load requirements. Such modulation is critical for maintaining a balance between energy availability and consumption, highlighting the practical aspects of managing a renewable energy system.

**Fig. 18:**
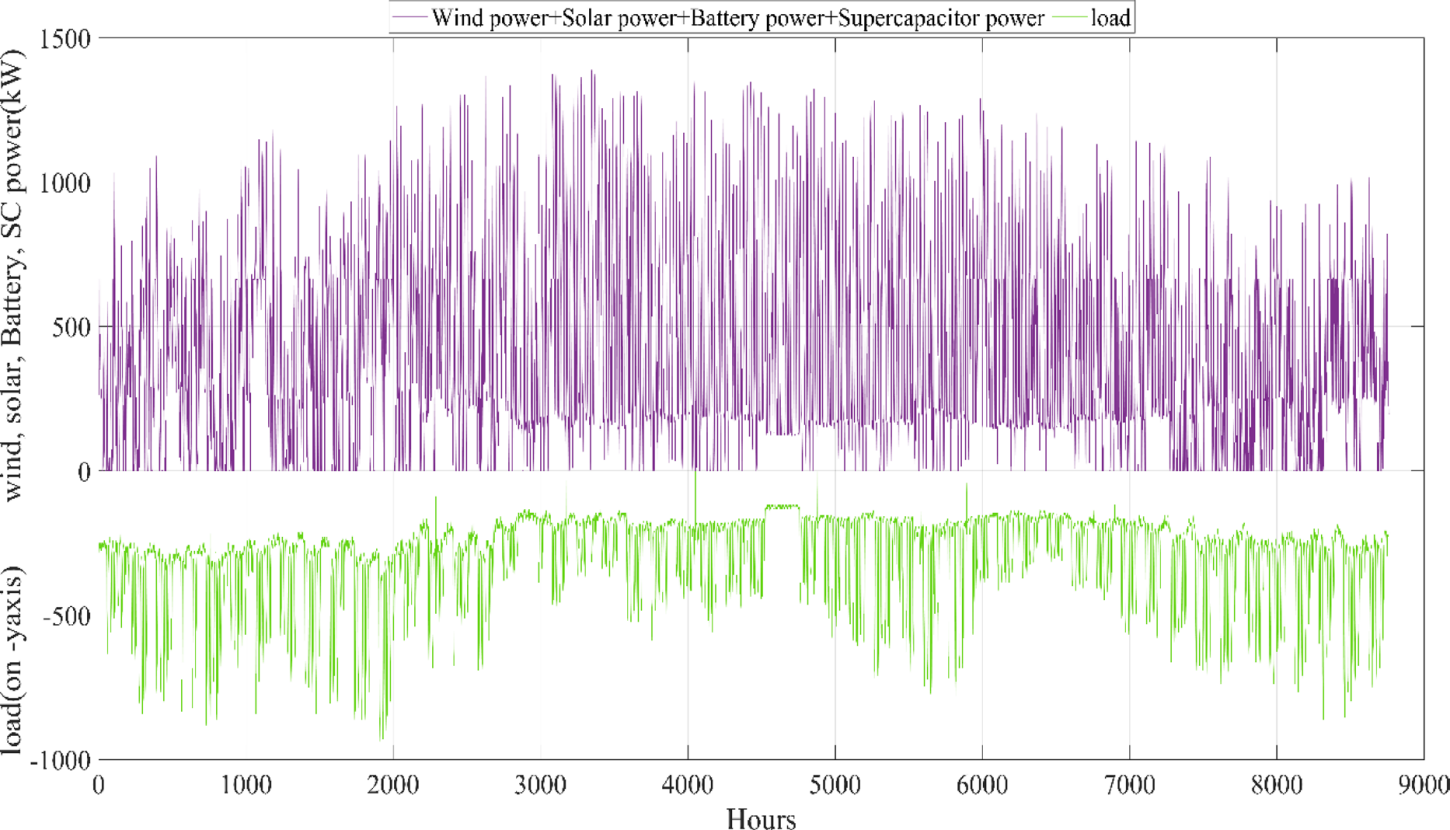
Annual energy production and consumption in the proposed HRES (HGS algorithm).

Figure 18 illustrates the complex interaction between energy production and consumption in a HRES over an annual cycle. The purple line represents the total energy output, incorporating contributions from wind and solar sources, BT storage, and SC. This line frequently exceeds the green line, representing the system load and indicating periods of energy surplus. These instances demonstrate the system’s robust capacity to meet energy demands. Moments when supply temporarily surpasses demand highlight the critical role of storage components. These components effectively bridge the energy gap, confirming their ability to balance fluctuations between production and consumption within the system. The graph captures the dynamic harmony between variable renewable energy flows and the stabilizing effect of storage technologies, emphasizing their collective significance in enhancing the operational resilience and sustainability of HRES.

**Fig. 19:**
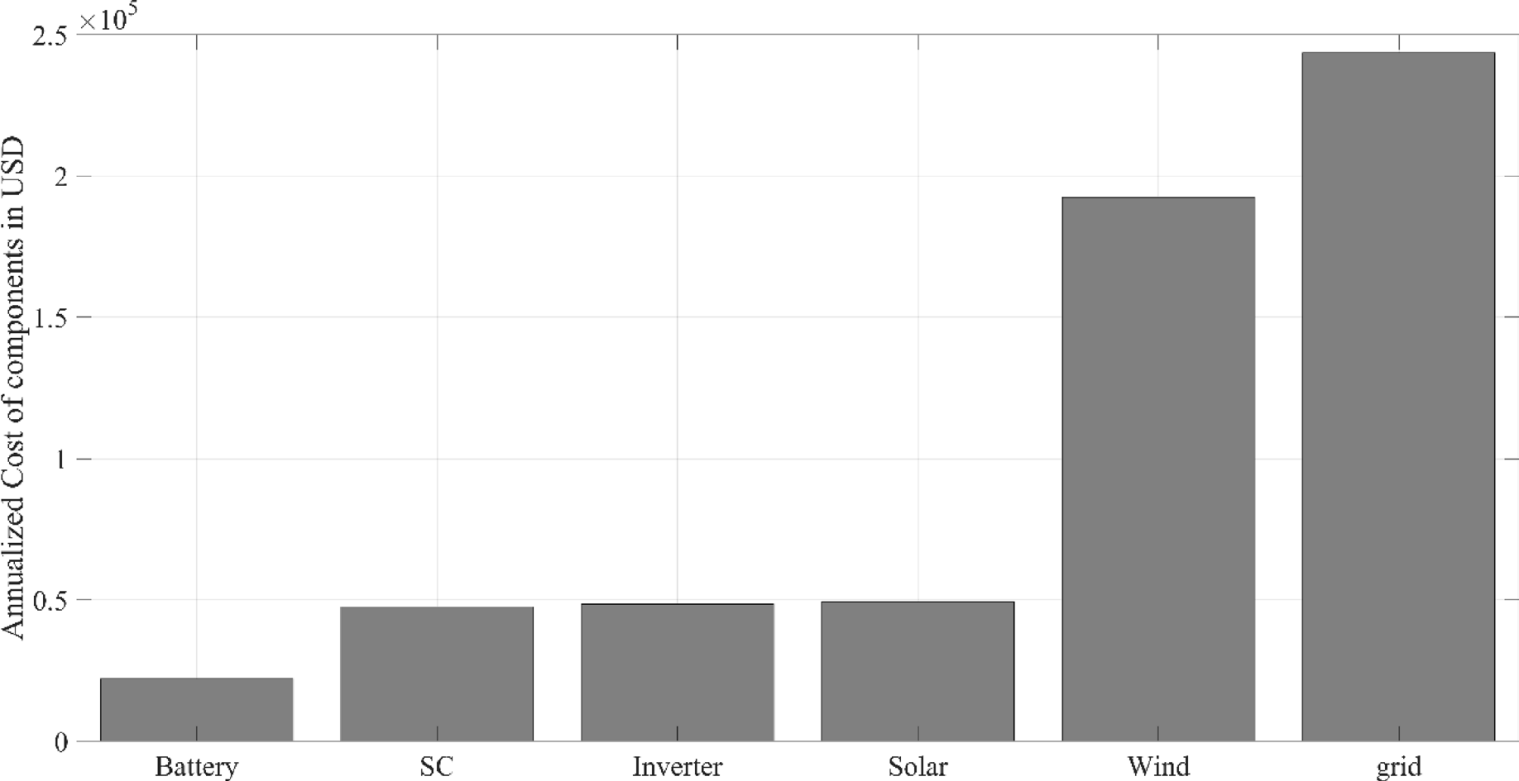
Cost analysis of a grid-connected HRES incorporating PV, WT, BT, and SC.

A cost comparison of the components of the optimized HRES was conducted, and the results are shown in Fig. 19. The proposed HGS method produced the following optimal values: $192,548.6735 for WT, $49,388.7580 for PV, $22,070.5663 for BT, $48,646.0361 for SC, $47,544.5858 for inverter, $243,339.8157 for grid cost, and $603,538.4357 for total annual cost.

**Fig. 20:**
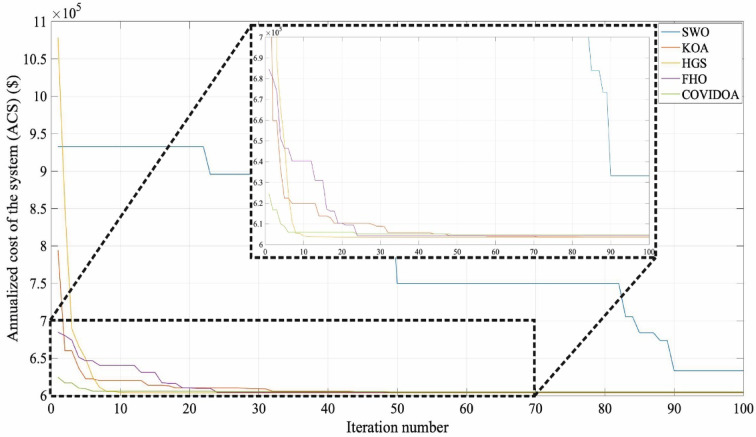
Comparative analysis of convergence patterns for the HGS, KOA, COVIDOA, FHO, and SWO optimization algorithms.

**Fig. 21:**
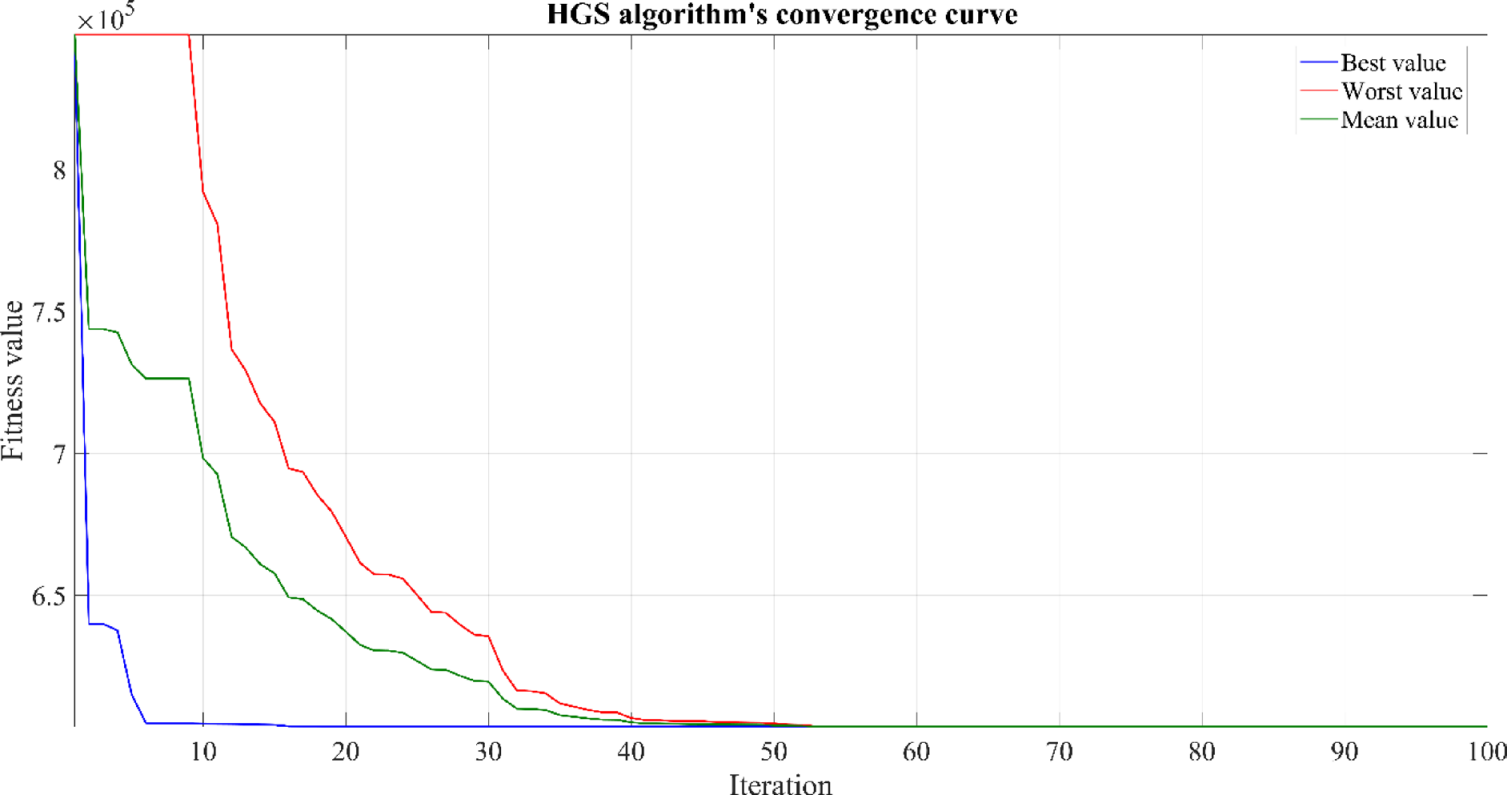
Convergence patterns for HGS optimization algorithms.

The results comparing the convergence performances of the algorithms are illustrated in Fig. 20. When compared with other algorithms, HGS, COVIDOA, and KOA demonstrate faster convergence toward the objective function, as shown in Fig. 20. The COVIDOA algorithm converges by the 6th iteration, KOA by the 11th iteration, and the HGS algorithm reaches convergence by the 9th iteration. By the end of 100 iterations, the HGS algorithm arrived at the optimum result more quickly than the others. Despite achieving similar and close results, HGS outperforms all other algorithms in terms of the lowest ACS values, as shown in Fig. 21, indicating superior performance and completion of the optimization process.

The proposed system configuration demonstrated superior performance in terms of cost-effectiveness, renewable energy penetration, and overall energy management reliability. The HGS-based design achieved an annualized cost of $603,538.44, a cost of energy of $0.23801/kWh, and a renewable energy fraction of 80.04%, surpassing the outcomes of the other benchmarked algorithms. These metrics are highly competitive when compared with recent studies in the literature, where similar HRES configurations often report higher COE values and lower REF due to either simplified modeling or limited optimization scope. Furthermore, the utilization of real meteorological and load data specific to the selected Turkish location enhances the validity and applicability of the results. While the method is geographically constrained, the general optimization framework and multi-algorithm assessment approach offer broad adaptability. The integration of battery and supercapacitor units provided a balanced hybrid energy storage solution, effectively mitigating power fluctuations and reducing stress on individual storage elements. Overall, the findings validate the effectiveness of the proposed methodology and confirm its practical potential for energy systems with similar climatic and operational conditions.

### Sensitivity analysis of HRES

Sensitivity analysis is an invaluable tool that can be employed to assess a system’s performance by analyzing the impact of variations in key parameters on system optimization. In this study, the effects of two primary parameters were evaluated: load variation and SC charge efficiency. By altering these parameters and analyzing the resultant changes in system optimization, it is possible to gain insights into the performance of the hybrid energy system under different conditions.

#### Impact of load variations

A sensitivity analysis was conducted to evaluate the responsiveness of the HRES to load variations. The effects of load fluctuations on system cost and sizing were examined with increments and decrements of 10%, 20%, and 30% compared with the nominal load. The analysis indicated that reductions in load significantly decreased system costs. As shown in Table 3, a 30% reduction from the nominal load decreased the annual cost from $603,541.05 to $410,074.27, marking a noticeable improvement in cost efficiency. Conversely, increases in load were observed to significantly raise system costs; a 30% increase boosted the annual cost by nearly 32%, taking it up to $798,437.92.

**Table 3:** The results obtained from evaluating the effects of load fluctuations on the sizing and cost of the HRES.

Variations in the load	10% Decrease	20% Decrease	30% Decrease	Nominal load	10% Increase	20% Increase	30% Increase
Best value ($)	539,021.81	474,512.65	409,998.26	603,541.05	668,113.02	733,047.52	798,437.92
Worst value ($)	757,776.16	806,505.92	905,948.06	849,774.19	784,571.18	821,343.05	1,313,420.75
Mean value ($)	544,906.44	479,092.16	418,316.25	610,370.34	670,961.02	734,293.82	804,061.67
WT power (kW)	599.6165	532.6256	474.273823	663.5052	732.1018	795.3123	852.4271
PV power (kW)	521.5256	460.9738	410.1214	587.2726	639.3433	680.3499	742.4693
SC size (kW)	512.1408	362.8521	192.19775	668.2729	800	800	800
BT size (kWh)	1000	1000	1000	1000	1000	1000	1000
SC output energy(kWh)	86,377.81	58,652.01	30,131.83	116,044.73	142,565.99	149,517.19	156,391.77
Total WT energy(kWh)	1,875,175.41	1,665,675.48	1,483,192.32	2,074,974.03	2,289,495.63	2,487,173.26	2,665,787.84
Total PV energy(kWh)	1,022,980.06	904,206.91	804,459.18	1,151,943.69	1,254,081.27	1,334,516.27	1,456,364.31
Total grid energy generation(kWh)	457,098.47	406,147.00	350,754.05	507,202.94	562,566.53	643,916.31	723,008.30
REF (%)	79.97	79.98	80.24	80.0	79.83	78.84	78.07
Annual cost $$(\mathrm{\$}$$)	539,021.81	474,512.65	410,074.27	603,541.05	668,113.02	733,047.52	798,437.92
Total annual load (kW)	2,282,108.3658	2,028,540.7696	1,774,973.173	2,535,462.1019	2,789,243.56	3,042,811.1543	3,296,379

**Fig. 22:**
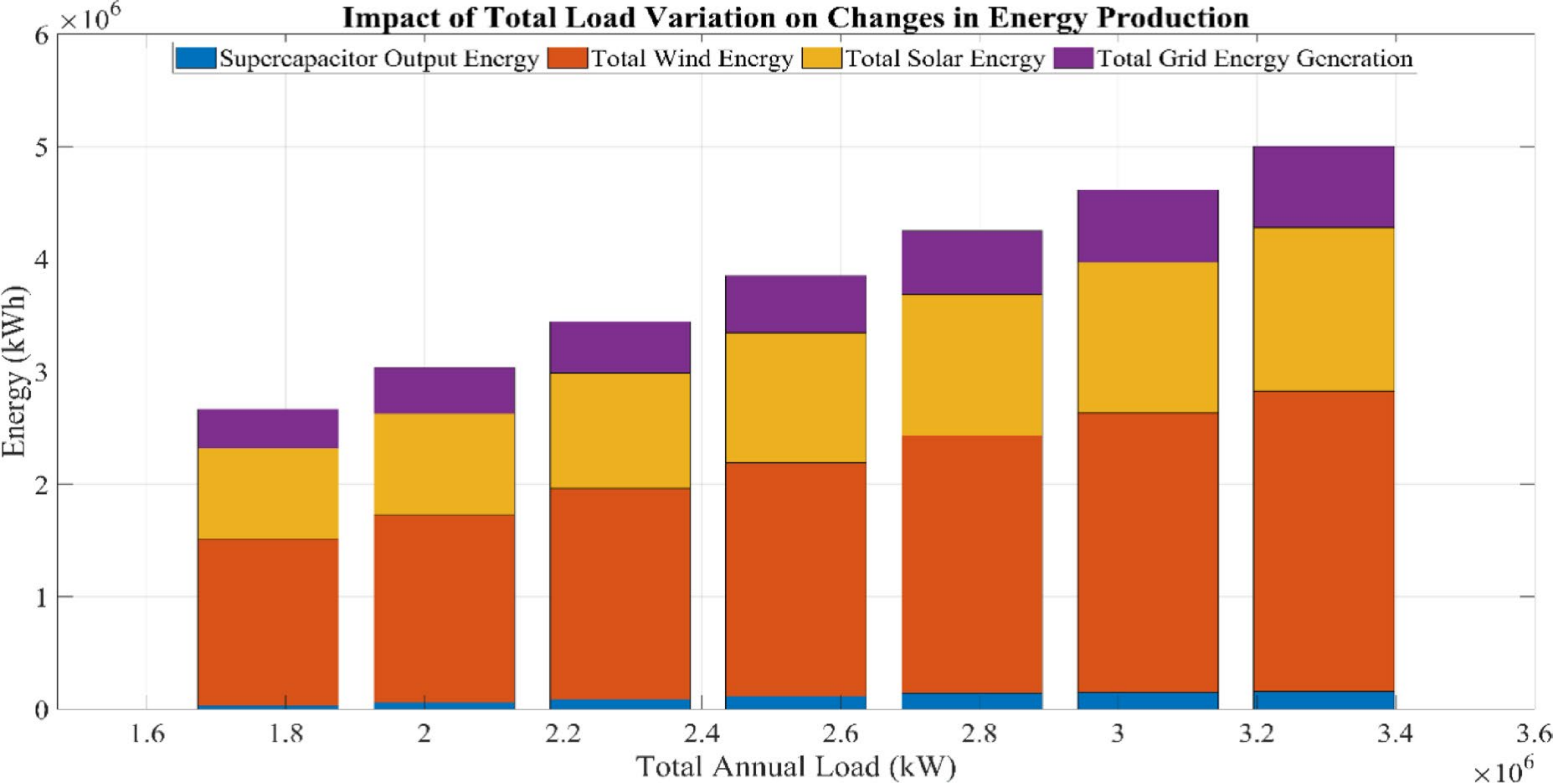
Effect of load change on HRES energy production.

In terms of energy production capacity, both WT and PV panels showed increases and decreases in power generation relative to the nominal load. The WT power decreased from nominal 663.5052 kW to 474.273823 kW with a 30% reduction and increased to 852.4271 kW with a 30% rise. PV power followed a similar trend, although the rate of increase remained lower than that in the WT. The SC power was capped at 800 kW, which was the upper limit set in the system design and optimization. Observing the system’s energy production capacity in Fig. 22, the total WT and PV energy varied in accordance with load changes. The SC output energy, which was 116,044.73 kWh under nominal load, increased to 156,391.77 kWh with load increments. Wind turbine energy production showed a proportional increase with load variations, reaching 2,665,787.84 kWh with a 30% increase from the nominal load. Similarly, the energy generated by PV panels also increased with load, rising to 1,456,364.31 kWh, which is a 30% increase from the nominal load.

**Fig. 23:**
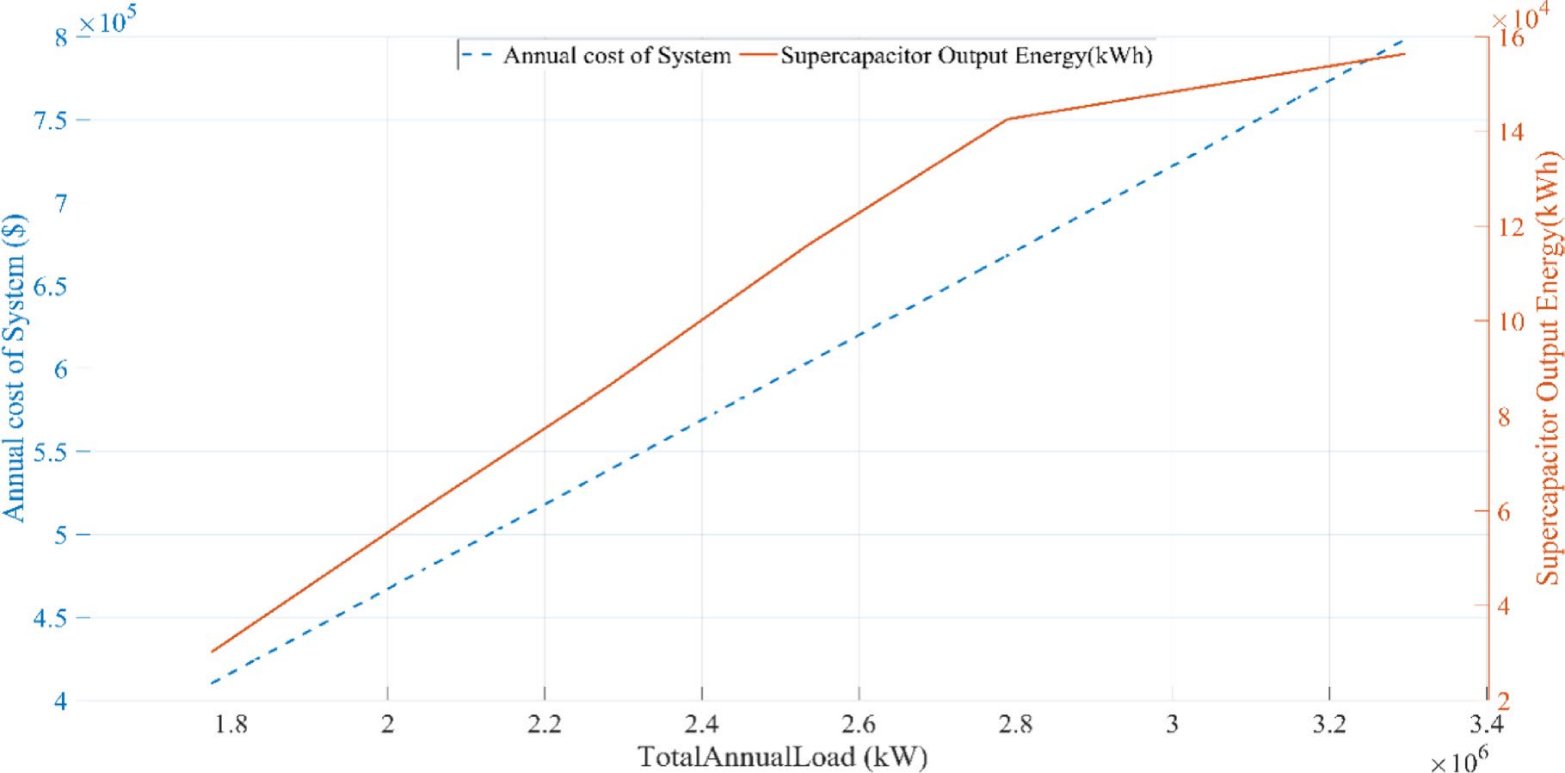
Relationship between total annual load, supercapacitor output energy, and ACS.

The data demonstrate that the HRES can dynamically adjust its energy production capacity in response to load variations. This highlights the flexibility of energy production sources and storage solutions to accommodate load changes and the implications of this flexibility on costs. It has also been found that grid energy production correlates directly with load changes. Under nominal load, the grid energy was 507,202.94 kWh, which increased to 723,008.30 kWh with a 30% increase in load, indicating an increased reliance on the grid when the system load escalates. According to the analysis, while the REF percentage is at 80% under nominal load, it slightly increases with load reductions but decreases with load increments. This suggests that increases in load could diminish the efficiency of HRES’s use of renewable energy. As shown in Fig. 23, the total annual load increases alongside the SC output energy, and the system cost also rises linearly.

In conclusion, load variations have a significant impact on the sizing and costs of the HRES, and these impacts could have substantial consequences for the system’s financial viability and environmental efficiency. Therefore, accurate load management and energy demand forecasting during system design and operation are critical for cost and efficiency optimization.

### Impact of supercapacitor charging efficiency variations

**Fig. 24:**
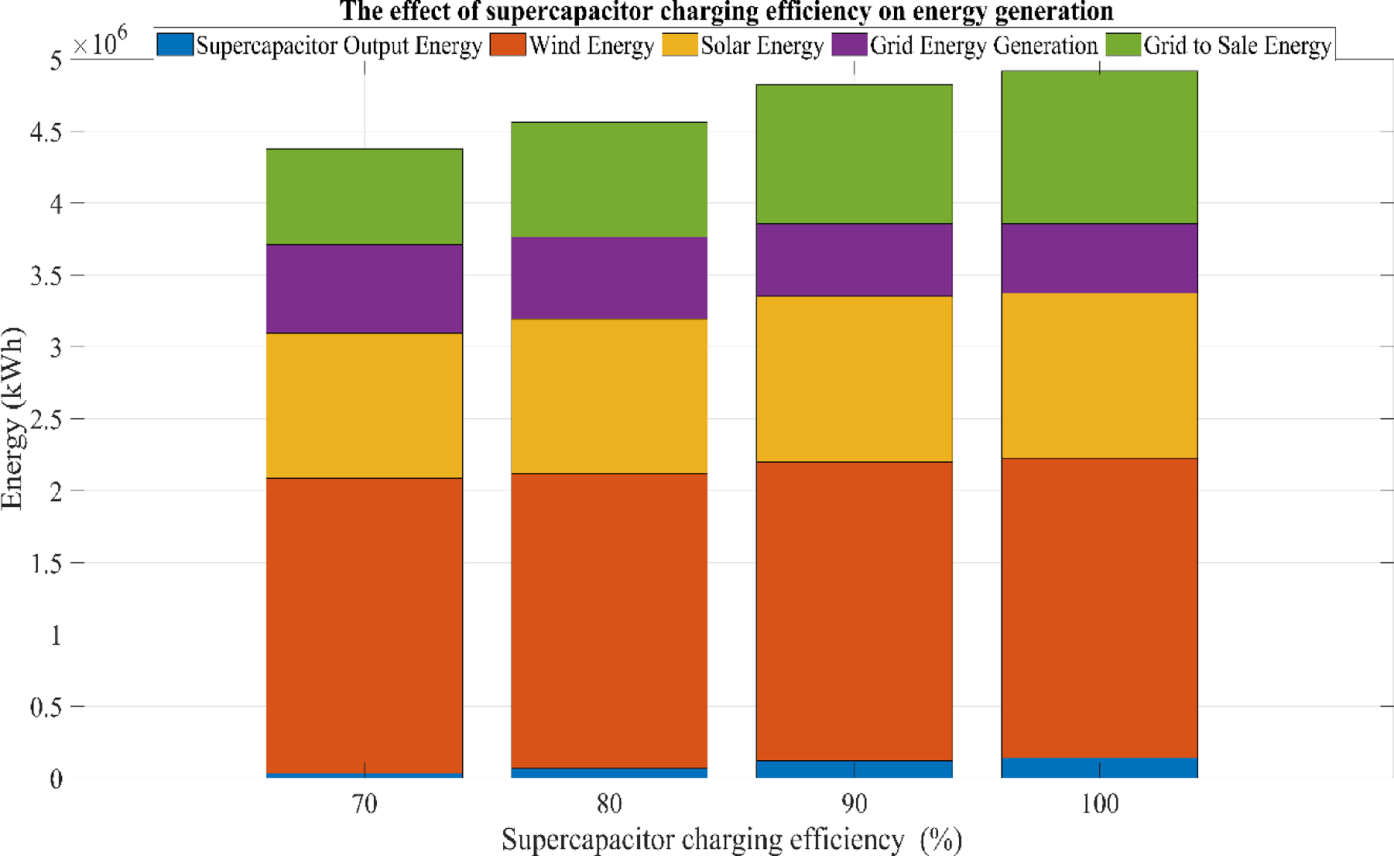
Effect of the SC charging efficiency on energy generation.

**Fig. 25:**
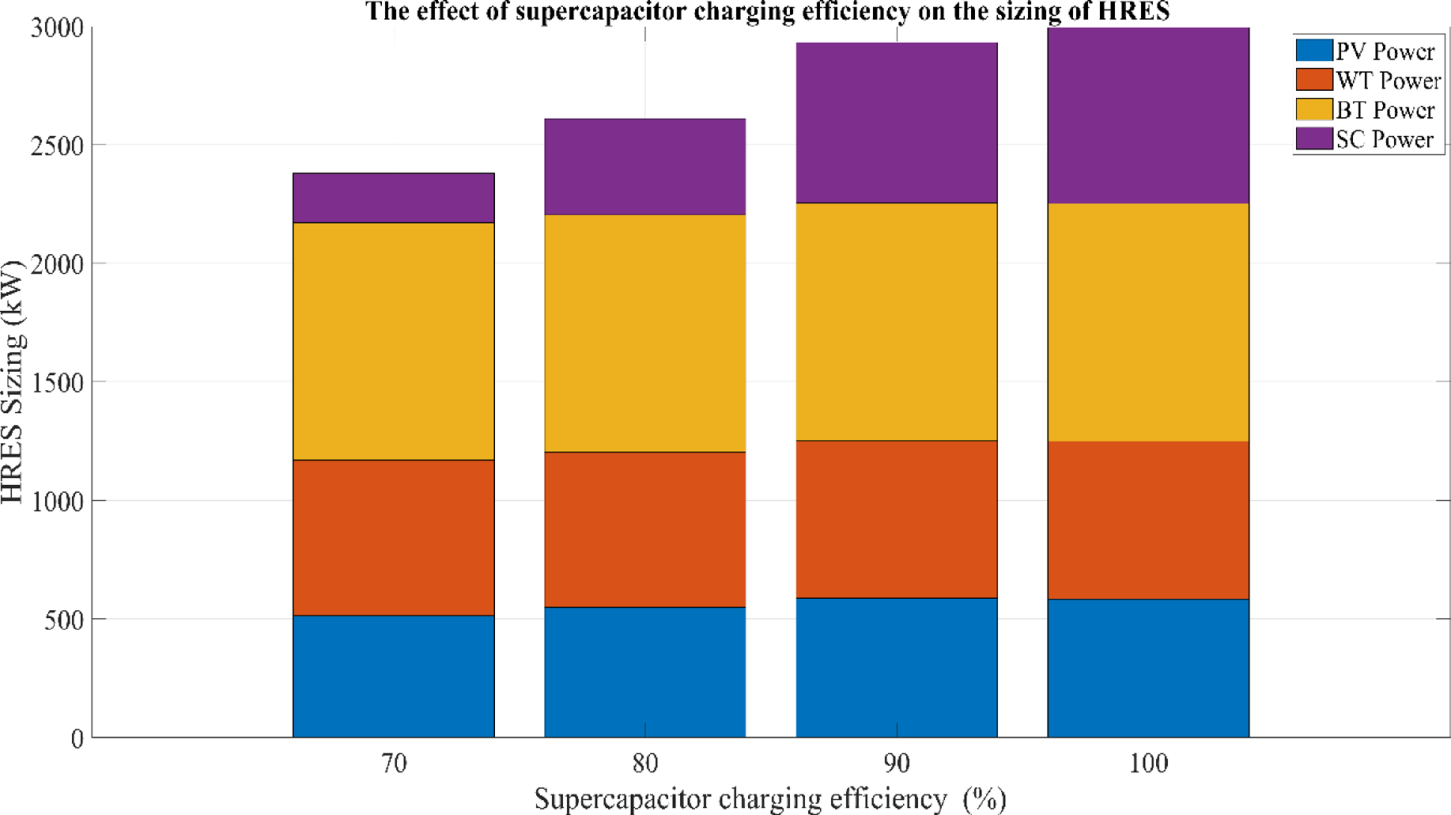
Effect of SC charging efficiency on HRES sizing.

**Fig. 26:**
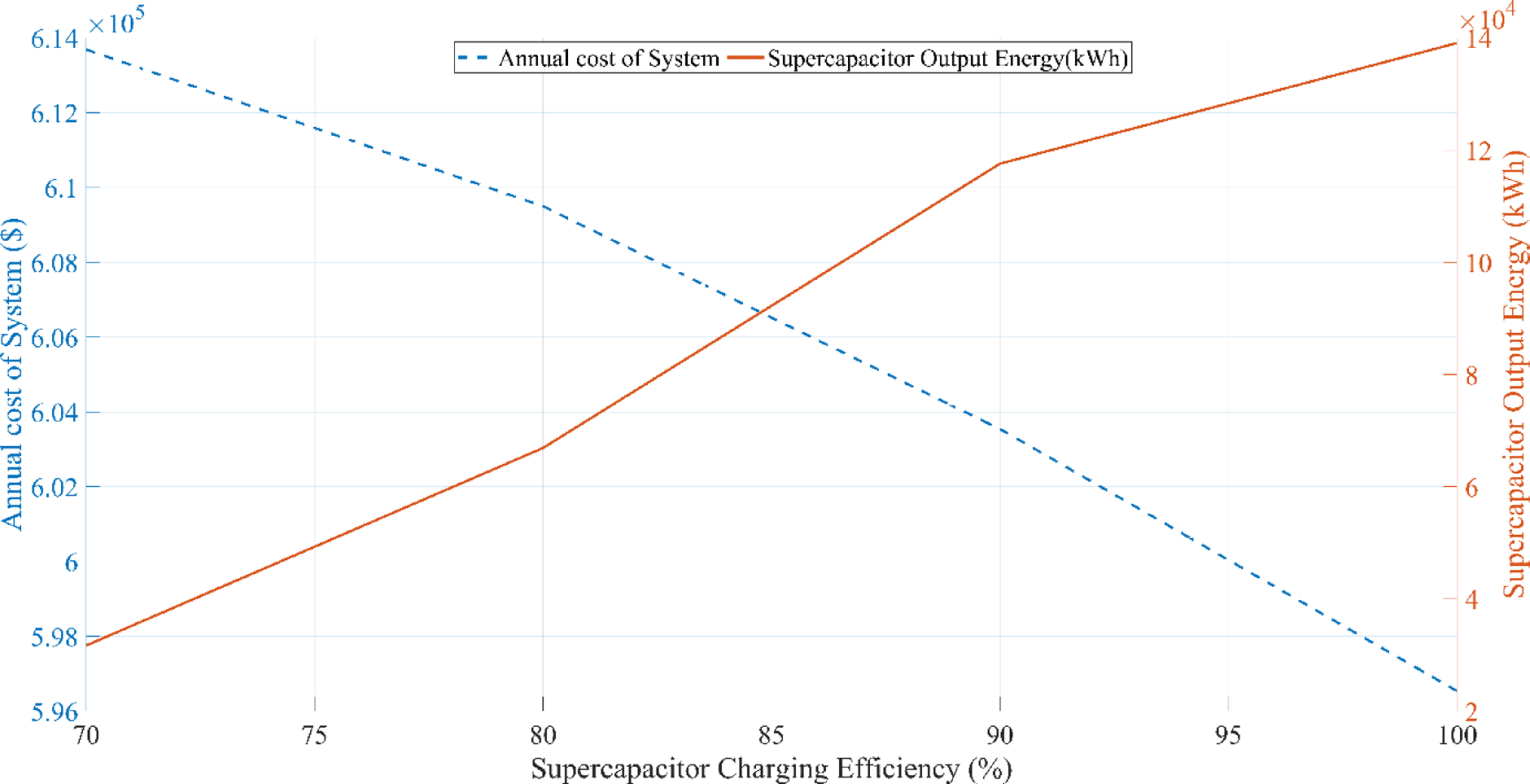
Trade-off Analysis between Supercapacitor Charging Efficiency and System Economics.

Table 4 provides a detailed analysis of the impact of changes in SC charge efficiency on HRES sizing and annual costs, based on sensitivity analysis. As seen in Fig. 24, when the charge efficiency drops from 100 to 70%, the wind turbine’s power output remains relatively stable, while there is a notable decline in photovoltaic power, particularly from the SC. This decrease is further illustrated in Fig. 25, where SC output energy is reduced by 52% at 80% efficiency and 77% at 70% efficiency. Additionally, as SC charge efficiency decreases, the system becomes more reliant on external energy sources, with an increase in grid energy purchases and a corresponding decrease in energy sold back to the grid. This reduced efficiency leads to higher annual costs, as shown in Fig. 26. The sensitivity analysis highlights the critical role of charge efficiency in the overall cost-effectiveness and sustainability of the HRES, making it a key factor in optimizing system design and operational strategies. These findings are essential for the effective optimization and cost management of renewable energy systems.

**Table 4:** Sensitivity analysis results of the supercapacitors charging efficiency on the dimensioning and cost of the HRES .

Supercapacitor charging efficiency	%100	%90	%80	%70
WT power (kW)	666.9506	664.9649	654.9084	655.9796
PV power (kW)	584.5172	587.7787	548.9931	514.6733
SC size (kW)	743.7763	678.5157	406.0096	208.7487
BT size(kWh)	1000	1000	1000	1000
SC output energy(kWh)	139,087.75	117,592.36	66,911.95	31,686.59
Total WT energy(kWh)	2,085,748.73	2,079,539.11	2,048,089.29	2,051,439.43
Total PV energy(kWh)	1,146,539.02	1,152,936.42	1,076,857.89	1,009,539.27
Total grid energy generation(kWh)	483,177.51	504,790.90	571,369.48	616,294.19
Grid to sale (kwh)	1,063,201.64	967,330.03	799,924.19	666,734.49
Annual cost $(\mathrm{\$}$)	596,534.14	603,541.21	609,497.62	613,689.74

### Statistical analysis of algorithms

Statistical analysis is a vital tool for extracting meaningful insights from complex datasets. These methods enable the identification of patterns, trends, and relationships that might otherwise be overlooked. In the context of this study, an array of statistical tests was employed to meticulously compare the performance of the five distinct algorithms. The primary objective behind these tests was not only to determine potential variances in the performance metrics of the algorithms but also to determine whether any observed differences were statistically significant. This ensures that the conclusions drawn are robust and not a result of random fluctuations in the data^81^.

In this study, an assortment of statistical tests was used to rigorously compare the performance of five distinct optimization algorithms: KOA, HGS, COVIDOA, FHO, and SWO. The results were statistically evaluated to determine the overall efficacy and robustness of the algorithms. To assess the performance of these algorithms, the mean of the results from each iteration was calculated to establish a general criterion of success. The means and standard deviations were computed using Eqs. (57) and (58), respectively. A stability analysis was also conducted to evaluate the performance of the algorithms under different initial conditions and iterations. This analysis measured the variability among the results of the algorithms using standard deviation values:

57 $${\mu }_{algorithm}=\frac{1}{n}\sum_{i=1}^{n}{x}_{i}^{algorithm}$$

58 $${\sigma }_{algortihm}=\sqrt{\frac{1}{n}\sum_{i=1}^{n}{({x}_{i}^{algorithm}-{\mu }_{algorithm})}^{2}}$$

where ${\mu }_{algorithm}$ represents the mean success of the algorithm, $n$ is the total number of iterations, and ${x}_{i}^{algorithm}$​ represents the value in the ith iteration of the respective algorithm. Table 5 provides a comprehensive summary of the mean, standard deviation, minimum, and maximum results obtained using the different algorithms used in the study. During the statistical analysis, the accuracy and stability of the model were assessed over 30 iterations.

**Table 5:** Mean, standard deviation, minimum, and maximum results of the algorithms.

Algorithm	ACS				
	Mean ( μ)	Median	Standard Deviation(σ)	Min	Max
KOA	636,500.138373	623,222.396050	36,886.775311	606,625.171500	768,917.519200
HGS	621,718.196667	603,595.350000	68,029.952177	603,538.100000	973,662.110000
COVIDOA	606,193.459550	605,041.275000	2940.394936	603,738.354800	615,356.596300
FHO	634,824.489170	622,265.995150	32,824.492552	605,533.400900	740,352.265600
SWO	805,500.439620	772,640.430850	150,146.795473	632,905.052300	1,250,298.783000

In this study, a thorough statistical examination was performed using five distinct optimization algorithms: KOA, HGS, COVIDOA, FHO, and SWO. For each algorithm, the mean efficacy (denoted as ${\mu }_{algorithm}$) was determined over numerous iterations ($n$), where ${x}_{i}^{algorithm}$ signifies the outcome of each specific iteration. Comprehensive data, including the mean, standard deviation, and the range of minimum to maximum results for these algorithms, are systematically presented in Table 5. The methodology involved conducting statistical analysis over a span of 30 iterations to critically evaluate the model’s precision and consistency.

Statistical analyses revealed significant differences among the five algorithms tested. KOA algorithm exhibited considerable variability in performance, indicated by a high standard deviation (σ) of 36,886.77, suggesting sensitivity to experimental conditions or parameter settings. Despite a broad range (606,625.17–768,917.51), its median performance (623,222.40) remained close to that of the FHO algorithm, which showed less variance in results (σ = 32,824.49). The HGS algorithm, with the lowest mean of 621,718.20, demonstrated substantial fluctuations, expanding between 603,538.10 and 973,662.11, indicating the potential for extreme outcomes under certain conditions. The COVIDOA algorithm demonstrated remarkable stability, as evidenced by its minimal standard deviation of 2,940.39, but it also recorded the lowest mean performance of all the algorithms, at 605,041.27. In contrast, the SWO algorithm, despite having the highest mean performance (805,500.44), showed the greatest inconsistency with the largest standard deviation (σ = 150,146.79), possibly due to overfitting or sensitivity to specific datasets. The peak performance of SWO reaching up to 1,250,298.78 suggests excellent potential under favorable conditions but also highlights the risk of performance declines, necessitating careful optimization. Collectively, these findings indicate that while KOA and FHO balance reliability and performance, COVIDOA is the most stable, HGS is volatile, and SWO, though potentially superior, requires meticulous optimization to mitigate performance fluctuations. It is crucial to note that these performances were evaluated over 30 iterations.

**Fig. 27:**
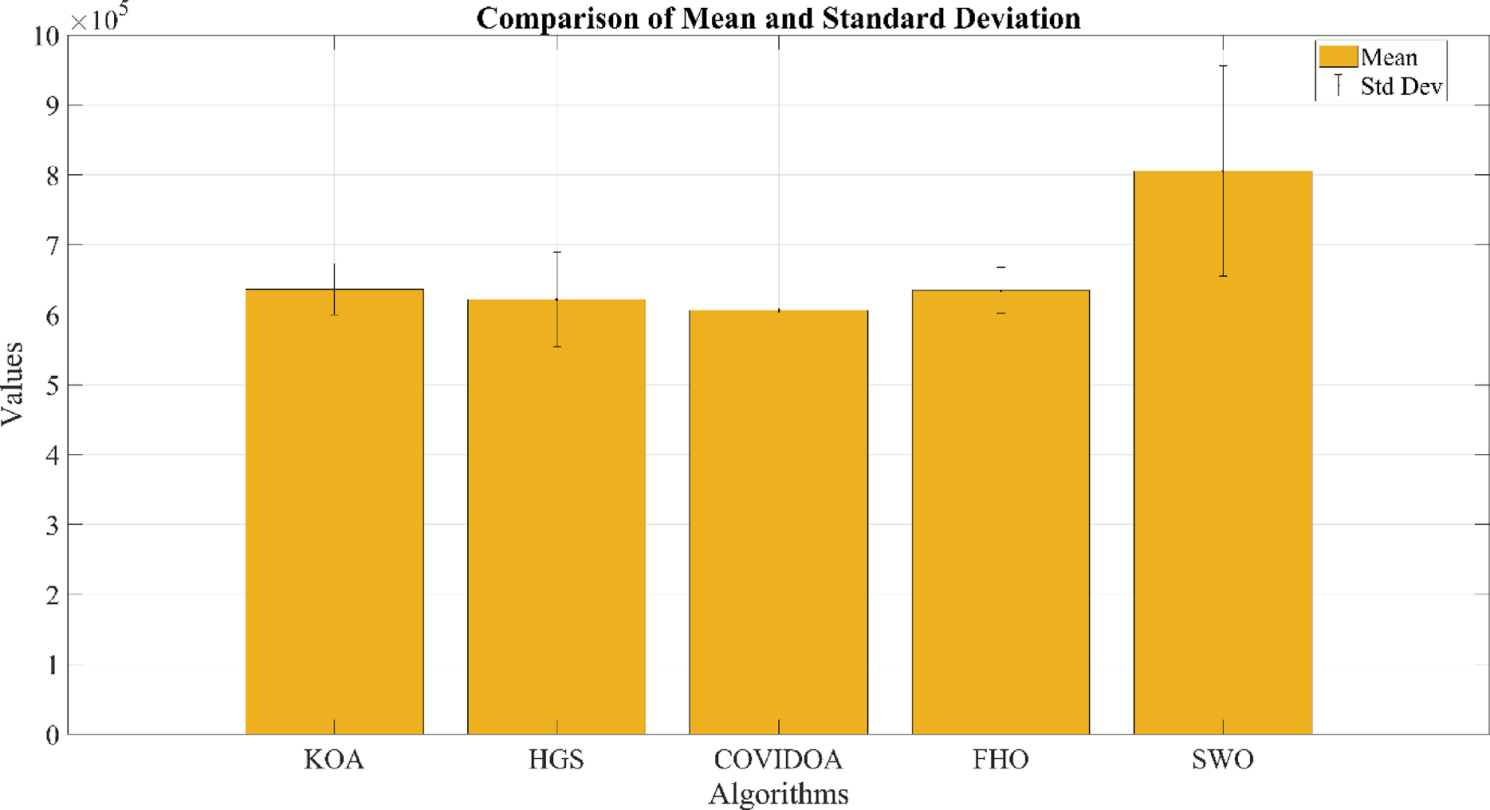
Comparative performance distribution of the KOA, HGS, COVIDOA, FHO, and SWO algorithms.

In the graphical analysis presented in Fig. 27, the KOA algorithm is characterized by a high mean value; However, its substantial standard deviation suggests a potential for variability in the results. Conversely, the COVIDOA algorithm, while exhibiting the lowest mean values, delivers the most consistent outcomes with the smallest standard deviation. This consistency indicates stable performance even under varying datasets and testing conditions. The FHO and HGS algorithms, although similar in mean values, differ significantly in terms of volatility; the high standard deviation of HGS points to potentially greater fluctuations compared with FHO. Meanwhile, the SWO algorithm, with the widest observed range of standard deviation, displays the highest variance. This implies that under certain conditions, SWO can achieve superior performance, but its outcomes can vary substantially under different scenarios. The comparative analysis of the algorithms provides valuable insights into algorithmic advantages and limitations, enhancing the understanding of practical applicability and performance across diverse contexts.

**Fig. 28:**
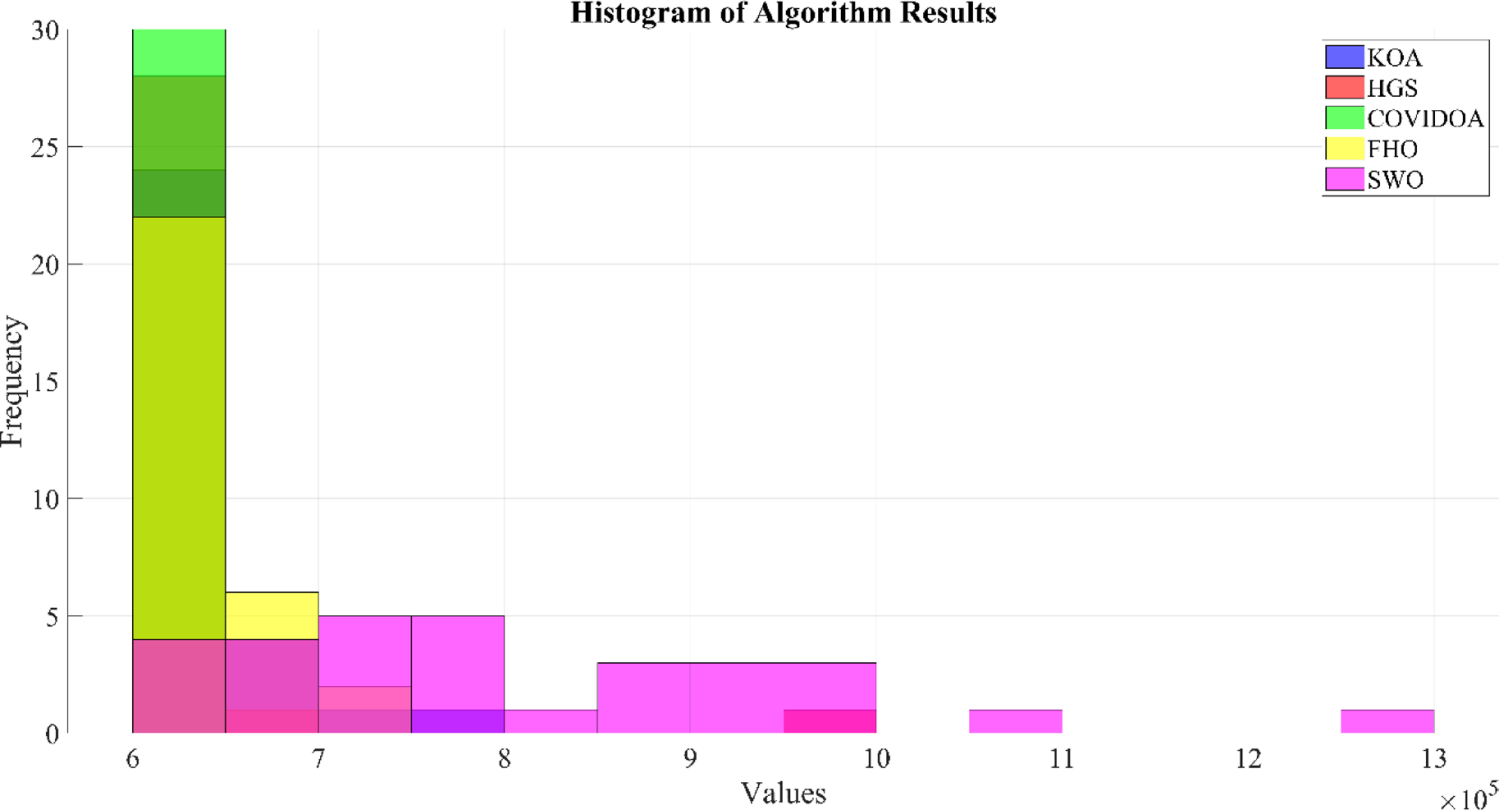
Probability distribution of the results for the KOA, HGS, COVIDOA, FHO, and SWO algorithms.

Figure 28 illustrates the distribution of algorithmic outcomes across various categories. The FHO category (Yellow) dominates with the highest bar frequency in the histogram, predominantly clustering around approximately 700,000. This suggests that most values generated by the FHO algorithm are centered around this figure. In contrast, the COVIDOA category (Green) shows the next highest frequency, centered around 600,000. Although slightly less frequent than the FHO, it still marks a significant concentration of results. The KOA (Blue) and HGS (Red) categories are represented with similar frequencies near the 700,000 mark, but their combined total is less than that of FHO. These categories overlap in the histogram, indicating that the results from both are common in this range. The SWO category (Pink) is characterized by lower frequency bars at the 800,000, 1,200,000, and 1,300,000 marks, suggesting that results for this category are more sparse and clustered at specific values compared with the others. Overall, this histogram reveals the concentration of algorithmic results in different categories and value ranges. FHO and COVIDOA are predominant in algorithmic outputs, whereas KOA and HGS show moderate representation, and SWO appears less frequently.

#### Cohen testi

In this study, Cohen’s d effect size was used to quantify the magnitude of the mean differences between the two groups. Mathematically, Cohen’s d is defined by Eq. (59)^82^:

59 $$d=\frac{{M}_{1}-{M}_{2}}{{SD}_{pooled}}$$

In this equation, ${M}_{1}$ and ${M}_{2}$​ represent the means of the two compared groups, while ${SD}_{pooled}$​ stands for the pooled standard deviation. The pooled standard deviation is computed as follows^83^:

60 $${SD}_{pooled}=\sqrt{\frac{\left({n}_{1}-1\right)\times {{SD}_{1}}^{2}+\left({n}_{2}-1\right)\times {{SD}_{2}}^{2}}{{n}_{1}+{n}_{2}-2}}$$

Here, ${n}_{1}$ and ${n}_{2}$​ denote the sample sizes of the two groups, and ${SD}_{1}$​​ and ${SD}_{2}$​ are their respective standard deviations. The categorization of effect size, as suggested by Cohen (1988), is as follows: a d value of 0.20 is considered small, 0.50 medium, and 0.80 large^84^. The statistical analyses conducted in this study assessed the performance differences between algorithms using Cohen’s effect size. A modest difference was observed between KOA and HGS (Cohen’s d = 0.270), whereas a notable performance disparity was found when comparing KOA to COVIDOA (Cohen’s d = 1.158), suggesting significant superiority of the COVIDOA algorithm over KOA. The nearly negligible effect size between KOA and FHO (Cohen’s d = 0.048) indicates no significant performance difference between these two algorithms. Conversely, SWO exhibited significantly lower performance than both KOA (Cohen’d = -1.546) and COVIDOA (Cohen’s d = -1.877), highlighting its considerable deficiencies relative to the other algorithms. The effect size between HGS and COVIDOA (Cohen’s d = 0.322) indicates a small but statistically significant difference, whereas HGS demonstrated better performance than FHO, as indicated by a negative effect size (Cohen’s d = -0.245). Additionally, a significantly large negative effect size between HGS and SWO (Cohen’s d = -1.577) suggests a pronounced superiority of HGS over SWO. COVIDOA and FHO displayed differences with a negative Cohen’s d value (-1.229), but SWO showed significantly lower mean values compared with both (Cohen’s d = -1.877 and -1.570). These analyses clearly delineate the performance relationships between the algorithms and the magnitude of these performance differences.

#### Wilcoxon rank sum test

The Wilcoxon rank sum test, also known as the Mann–Whitney U test, is used to compare the distributions of two independent sample groups^85^. It serves as an alternative to the t-test, particularly for datasets that do not conform to a normal distribution. This test provides statistical evidence that the medians of the two groups are distinct from each other. The p-value of the test aids in determining whether the medians are significantly different. A p-value less than 0.05 indicates a statistically significant difference between the two groups^86^.

The basic equation used for the Mann–Whitney U test (or Wilcoxon Rank Sum test) is as shown in Eq. (61).

61 $$U={n}_{1}\times {n}_{2}+\frac{{n}_{1}\times \left({n}_{1}+1\right)}{2}-{R}_{1}$$

In this analysis, ${n}_{1}$ and ${n}_{2}$ respectively denote the number of observations within the two compared groups. ${R}_{1}$ represents the sum of the ranks for the first group. Used for comparative purposes, this equation aids in assessing the differences between two groups. The $U$ value, a key statistical indicator, reflects the degree of dissimilarity in the distributions of these groups. A lower $U$ value indicates a more pronounced disparity between the groups^87^. It is important to note, however, that the use of this equation and the interpretation of its results are nuanced and require a degree of statistical expertise. The implications of statistical tests are subject to variations based on data structure, sample sizes, and other influencing factors. For the five algorithms applied in this research, the test results indicate the following;

The Wilcoxon rank sum test results, as presented in Table 6, statistically evaluate the differences between algorithms and highlight the significance of these differences using p-values. The p-value obtained from the comparison between KOA and HGS (2.84 $\times {10}^{-7}$) indicates a statistically significant difference between these two groups. The extremely low p-value (5.27 $\times {10}^{-12}$) in the comparison of KOA with COVIDOA signifies a much more pronounced performance disparity between KOA and COVIDOA. Conversely, the p-value between KOA and FHO (0.924) indicates no statistically significant difference. In the comparison of KOA and SWO (p = 2.31 $\times {10}^{-10}$), SWO was observed to have significantly lower performance than KOA. The test between HGS and COVIDOA (p = 0.0014) confirmed a significant performance difference between these two algorithms. The extremely low p-value (2.10 $\times {10}^{-7}$) between HGS and FHO indicates that HGS has a statistically significant superiority over FHO. The results of the test between HGS and SWO (p = 3.63 $\times {10}^{-12}$) show that HGS has a clear performance advantage over SWO.

**Table 6:** Wilcoxon rank sum test results.

Algorithm	Result (p-value)
KOA and HGS	2.841652 $\times {10}^{-7}$
KOA and COVIDOA	5.273413 $\times {10}^{-12}$
KOA and FHO	9.240175 $\times {10}^{-1}$
KOA and SWO	2.307665 $\times {10}^{-10}$
HGS and COVIDOA	1.443221 $\times {10}^{-3}$
HGS and FHO	2.101718 $\times {10}^{-7}$
HGS and SWO	3.631518 $\times {10}^{-12}$
COVIDOA and FHO	5.273413 $\times {10}^{-12}$
COVIDOA and SWO	1.691123 $\times {10}^{-17}$
FHO and SWO	7.957297 $\times {10}^{-11}$

The p-value in the comparison of COVIDOA and FHO (5.27 $\times {10}^{-12}$) demonstrates COVIDOA’s statistically significant superiority over FHO, and the lowest p-value obtained between COVIDOA and SWO (1.69 $\times {10}^{-17}$) emphasizes COVIDOA’s most pronounced performance advantage over SWO. The p-value in the comparison between FHO and SWO (7.95 $\times {10}^{-11}$) indicates that FHO is statistically significantly better in performance than SWO. When these test results are collectively considered, significant and statistically meaningful differences in performance among the algorithms are observed. The obtained p-values provide strong evidence regarding the superiority or similar performance of the algorithms. These results clearly indicate that COVIDOA generally has a distinct advantage over the other algorithms, whereas SWO exhibits relatively lower performance.

#### Correlation matrix analysis of algorithm performances

**Fig. 29:**
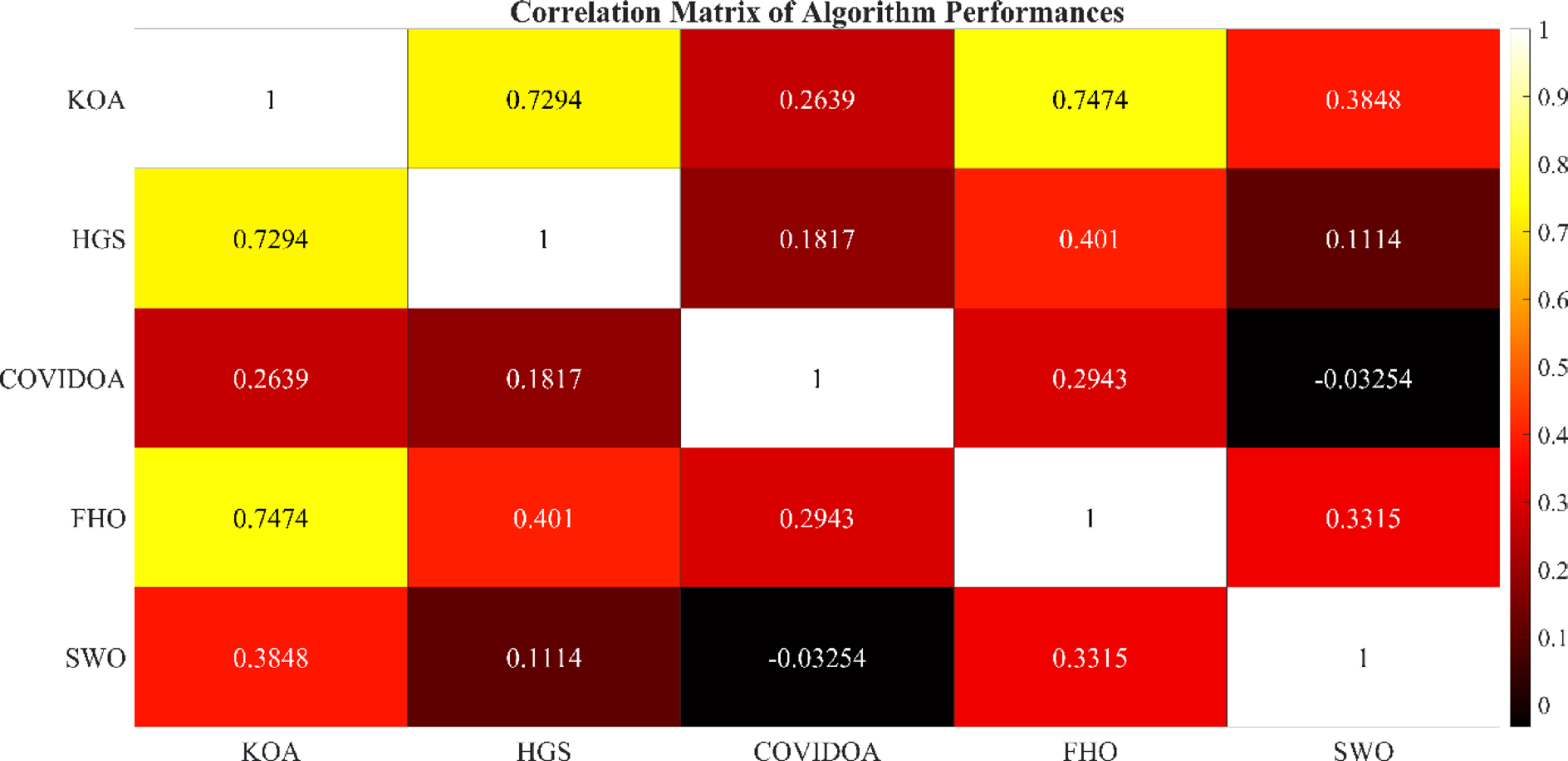
Correlation matrix of the performances for the KOA, HGS, COVIDOA, FHO, and SWO algorithms.

In this section, a comprehensive analysis of the relationships between different algorithm performances is conducted. To understand how the algorithms interact with each other and to grasp these relationships in detail, the correlation matrix method was employed. The primary goal of correlation matrix analysis is to determine the degree of compatibility or opposition between the performances of the selected algorithms and to use this information to make more informed algorithmic selections^88^. This analysis has clearly laid out the similarities and differences between the algorithms, guiding future algorithmic choices in subsequent research. Figure 29 presents the correlation matrix results of the algorithms. Here, the diagonal line of the matrix shows the correlation of each algorithm with itself, and these values are always 1. Therefore, these values are not considered in the evaluation. The intensity and shade of the colors represent the strength of the relationship between the algorithms^89^:

- Warm colors transitioning from yellow to red indicate a positive correlation; the brighter the color, the stronger the correlation.
- Dark colors (dark red and near blacks) indicate very low or negative correlation.

Examining the matrix in detail:

- The high correlation (0.7294) between KOA and HGS indicates a strong positive relationship between the results of these two algorithms.
- The similarly high correlation (0.7474) between KOA and FHO suggests that these two algorithms exhibit similar performance trends.
- The very low negative correlation (-0.03254) between COVIDOA and SWO shows that these two algorithms have weak and slightly negative performance relationships; that is, as one algorithm’s performance increases, a slight decrease in the performance of the other may be observed.
- The moderate positive correlation (0.3315) between FHO and SWO indicates a positive relationship between the performances of these two algorithms, although this relationship is weaker than that of the others.

These relationships between each algorithm’s performance and those of others provide important indicators for understanding the conditions under which the algorithms may exhibit similar or different performances. Such insights should be considered during algorithm selection or optimization.

#### Friedman test based on algorithms

In this study, the Friedman rank test, a powerful non-parametric statistical approach, was utilized to assess the significant performance differences between various algorithms through a series of comparative analyses^90^. This method evaluates the variance of rankings within the dataset, making it particularly well-suited for experiments that include k different groups (or treatments) and $N$ blocks (or matched samples). The Friedman test statistic, denoted as $\chi 2$, used in this analysis, is formulated in Eq. (62) as described in^91^.

62 $${\chi }^{2}=\frac{12N}{k(k+1)}\sum_{j=1}^{k}{R}_{j}^{2}-3N\left(k+1\right)$$

In the analysis performed, ${R}_{j}$​ denotes the sum of rankings for the jth group, while $N$ indicates the number of blocks, and $k$ represents the number of groups. Applying the Friedman rank sum test to data obtained from $k$ = 5 different algorithms over $N$ = 30 experimental iterations produced the following outcomes:

The obtained Friedman ${\chi }^{2}$ value was calculated to be 98.747, and for 4 degrees of freedom, the p-value was found to be less than 2.2e-16. This outcome indicates that at least one algorithm exhibits a performance that differs significantly from the others.

The Chi-square statistic functions as an indicator to assess the deviation between observed and expected frequencies. A greater ${\chi }^{2}$ value signals more pronounced differences among the groups, pointing to significant variations in the performance of the tested algorithms.

Following the Friedman test, a comprehensive post-hoc analysis was conducted using the Nemenyi test to examine the pairwise performance discrepancies among algorithm pairs. According to the results showcased in Table 7, significant performance differentials were observed between several pairs: FHO and COVIDOA, FHO and HGS, KOA and COVIDOA, KOA and HGS, as well as between SWO and all other algorithms (with respective p-values of 0.0016, 2.2 $\times {10}^{-5}$, 0.0003, 2.7 $\times {10}^{-6}$, and 1.2 $\times {10}^{-13}$). These findings highlight the statistical disparity in performance exhibited by the SWO algorithm when compared with its counterparts. Conversely, negligible performance differentials were noted between FHO and KOA (p = 0.9942) and HGS and COVIDOA (p = 0.8645), suggesting comparative parity in their performances.

**Table 7:** Nemenyi Wilcoxon Post-Hoc test results for algorithm comparisons (Friedman chi-squared = 98.747, df = 4, p-value < 2.2e-16).

	COVIDOA	FHO	HGS	KOA
FHO	0.0016	-	-	-
HGS	0.8645	2.2 $\times {10}^{-5}$	-	-
KOA	0.0003	0.9942	2.7 $\times {10}^{-6}$	-
SWO	1.2 $\times {10}^{-13}$	0.0006	4.0 $\times {10}^{-14}$	0.0030

#### Kruskal–Wallis and Tukey multiple comparisons test

The performance of the algorithms employed for system optimization was assessed using the Kruskal–Wallis test, a non-parametric statistical method, followed by Tukey’s multiple comparison test. The Kruskal–Wallis test is utilized to evaluate whether the medians of three or more independent groups are statistically similar^92^. This method examines the variance within and between groups, identifying significant differences in the performance of the algorithms^93^.

63 $$H=\frac{12N}{N(N+1)}\sum_{i=1}^{g}\frac{{\overline{r} }_{i}^{2}}{{n}_{i}}-3\left(N+1\right)$$

The Kruskal–Wallis $H$ statistic is computed using Eq. (63)^94^, which incorporates several key parameters: $N$, representing the total number of observations; $g$, the number of groups being compared; ${n}_{i}$​, the number of observations within the ith group; and ${\overline{r} }_{i}$​, which denotes the sum of ranks for the ith group. The equation includes ${\overline{r} }_{i}^{2}$​​, the squared sum of ranks for each group, and $N(N+1)$, a term that adjusts for the total number of observations. Higher H statistic values indicate significant median differences across the groups. The Kruskal–Wallis test evaluates the variance ratio by comparing the squared rank sums of each group against the total rank sum for all observations. The resulting $H$ value is then compared to a χ^2^ (chi-square) distribution, considering a specific degree of freedom (df). If the $H$ value surpasses the χ^2^ critical value at a given significance level, it suggests that there are statistically significant median differences among the groups, indicating that the performance differences are not random but meaningful^95^.

In this analysis, the Kruskal–Wallis test revealed significant cost differences among the algorithm groups (χ^2^ = 99.9962, df = 4, *p* < 0.001). With a p-value approaching zero, this result demonstrates that the observed cost performance variations among the algorithms are substantial and not attributable to random fluctuations.

Tukey’s test was employed to compare the mean differences between pairs of groups, based on the results of the analysis of variance (ANOVA). After performing ANOVA, Tukey’s post-hoc test was applied to group pairs where statistically significant differences were observed. The formula for Tukey’s test is provided in Eq. (64)^96^.

64 $$q=\frac{\left|{\overline{x} }_{i}-{\overline{x} }_{j}\right|}{\sqrt{\frac{MSE}{n}} }$$

In this formula, the $q$-value represents the absolute difference between the means of the two groups, normalized by a measure of error variance known as the mean square error (MSE), expressed as a standardized error. Here, ${\overline{x} }_{i}$ and ${\overline{x} }_{j}$ are the group means for algorithms i and j, respectively, $MSE$ is the mean squares for error, and n is the number of observations in each group. The Tukey test is used to compare with critical values to determine when the difference between two group means is considered statistically significant at a given confidence level^97,98^.

In this study, the Kruskal–Wallis test and Bonferroni correction were applied to evaluate the performance of five algorithms used (Table 8), and the results were further substantiated by the Tukey Multiple Comparison Test (Table 9). A comparison between COVIDOA and SWO revealed the lowest p-value and the highest Z-value, indicating that the SWO algorithm resulted in the highest expected costs for the COVIDOA scenario, suggesting a negative impact on the cost-effective optimization of energy systems. Conversely, a significant cost difference was identified in the comparison between FHO and HGS. The magnitude and statistical significance of this difference indicate that FHO is a more cost-effective solution than HGS. The results of the Tukey test, particularly in the SWO—COVIDOA and SWO—FHO comparisons, clearly demonstrate how these algorithms differentiate in various scenarios when considering confidence intervals and adjusted p-values. Such statistical analyses not only emphasize the importance of algorithm selection in the optimization of hybrid energy systems but are also crucial for making strategic decisions that could enhance the cost reduction potential during the design and operation of energy systems.

**Table 8:** Comparison of results with the Kruskal–Wallis test and Bonferroni correction.

Group Comparison	Z-Value	Adjusted P-Value (Bonferroni
COVIDOA—FHO	-4.177978	0.0001
COVIDOA—HGS	0.662652	1.0000
COVIDOA—KOA	-4.148263	0.0002
COVIDOA—SWO	-7.996401	0.0000
FHO—HGS	4.840631	0.0000
FHO—KOA	0.029715	1.0000
FHO—SWO	-3.818422	0.0007
HGS—KOA	-4.810915	0.0000
HGS—SWO	-8.659053	0.0000
KOA—SWO	-3.848138	0.0006

**Table 9:** Tukey multiple comparison test results:

Comparison	Mean difference (diff)	Lower confidence interval (lwr)	Upper confidence interval (upr)	Adjusted p-value (p adj)
FHO—COVIDOA	28,631.030	-26,265.01	83,527.07	0.6024750
HGS—COVIDOA	15,524.737	-39,371.31	70,420.78	0.9356862
KOA—COVIDOA	30,306.679	-24,589.36	85,202.72	0.5479655
SWO—COVIDOA	199,306.980	144,410.94	254,203.02	0.0000000
HGS—FHO	-13,106.293	-68,002.34	41,789.75	0.9645940
KOA—FHO	1675.649	-53,220.39	56,571.69	0.9999884
SWO—FHO	170,675.950	115,779.91	225,571.99	0.0000000
KOA—HGS	14,781.942	-40,114.10	69,677.98	0.9457451
SWO—HGS	183,782.243	128,886.20	238,678.29	0.0000000
SWO—KOA	169,000.301	114,104.26	223,896.34	0.0000000

**Fig. 30:**
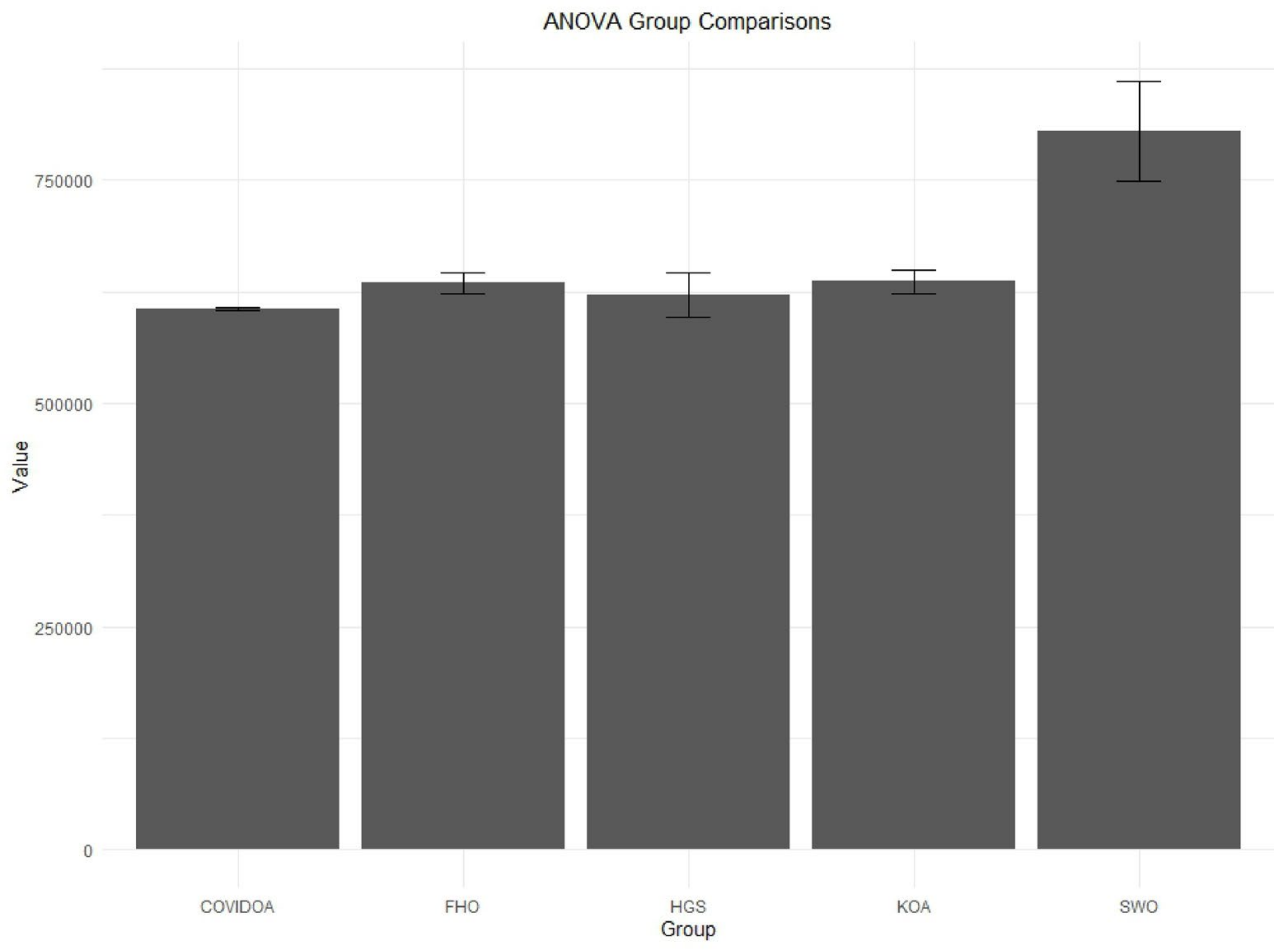
ANOVA group comparison analysis of the algorithms.

Figure 30 presents the data obtained in the R programming environment and analyzed using the ANOVA test. The variance analysis compares the performances achieved by different algorithms due to optimization. The analysis reveals that the COVIDOA algorithm has the lowest mean cost compared with other groups, whereas the SWO algorithm statistically exhibits higher values. This observation suggests that, under the specific conditions of the analyzed dataset, SWO demonstrates lower cost-effectiveness compared to the other evaluated algorithms. These results prompt further discussion and analysis of the use and applicability of the SWO algorithm. In summary, these findings highlight crucial factors that should be considered when comparing the cost-effectiveness of algorithms and developing optimization strategies.

## Conclusions

This study presents an optimization method using the HGS algorithm to construct a hybrid grid-connected RE system comprising PV, WT, BT, and SC components. By considering the REF parameter as a constraint, the method aims to minimize the target function ACS, thereby determining the ideal sizing of the RES. The optimum design created with HGS has demonstrated superior results over other optimization techniques such as HGS, SWO, KOA, FHO, and COVIDOA in terms of unit energy cost, annual system cost, and convergence times for the sizes of renewable resources chosen.

Sensitivity analyses conducted in this study and the effects of changes in SC charge efficiency significantly impact the performance of HRES. The sensitivity analyses on the system optimization revealed the effects of 10%, 20%, and 30% increases and decreases in load on system costs and energy production. A 30% increase in load led to a 32% rise in system cost compared with the nominal load condition, reaching $798,437.92, while a 30% decrease in load reduced the cost to $409,998.26. This highlights the significant impact of load variations on system design and costs. When the SC charge efficiency dropped from 100 to 70%, the WT power decreased from 666.9506 kW to 655.9796 kW, and the PV power decreased from 584.5172 kW to 514.6733 kW. This reduction led to a decrease in total WT energy from 2,085,748.73 kWh to 2,051,439.43 kWh and total PV energy from 1,146,539.02 kWh to 1,009,539.27 kWh. Moreover, the decrease in SC charge efficiency reduced the amount of energy sold to the grid from 1,063,201.64 kWh to 666,734.49 kWh. These results underscore the critical role of SC charge efficiency in system performance. The reliability of these findings is supported by statistical tests, such as the Cohen Test, Wilcoxon Rank Sum test, Correlation Matrix Analysis, Friedman Test, Kruskal–Wallis, and Tukey Multiple Comparisons Test, applied to the algorithms. These tests objectively assessed the performance of the algorithms and the results obtained over 30 repeated iterations, clearly revealing the performance differences among them.

In conclusion, this study has shown that factors such as load variations and SC charge efficiency have significant impacts on the cost and performance of HRES. The HGS algorithm, compared with other algorithms used, provides valuable insights for the design and optimization of HRES and lays a solid foundation for future research. Each algorithm used in this study successfully assessed the tendencies toward optimal results. As the algorithm approaches an optimal solution, the density of the search area can be automatically adjusted. Conversely, when deviating from the optimal solution, it can be expanded to explore new search points. The proposed HGS method has generated highly competitive results in solving the HRES sizing problem and is expected to be applied to more challenging problems in the future. In addition, this study highlights the usefulness of the optimization methods investigated, particularly in the context of hybrid grid-connected renewable energy systems. The presented results offer a solid foundation for understanding the dynamics of the system. However, the scalability and adaptability of these findings to different environments require further research. Policymakers and industry stakeholders can use the insights gained from this study to improve the integration of renewable energy systems. The effectiveness of the HGS optimization method shown here demonstrates its potential for enhancing the design and efficiency of renewable energy systems, paving the way for subsequent research in this field. Future work may involve reformulating the problem in a multi-objective framework to explore trade-offs between cost, renewable energy fraction, and system reliability.

## Supplementary Information

Below is the link to the electronic supplementary material.

Supplementary Material 1

## Data Availability

The datasets used and/or analyzed during the current study available from the corresponding author on reasonable request.

## Acronyms

ACS: Annual cost of the system
BT: Battery
CRF: Capital recovery factor
HGS: Hunger games search
SWO: Spider wasp optimizer
KOA: Kepler optimization algorithm
FHO: Fire hawk optimizer
COVIDOA: Coronavirus disease optimization algorithm
DOD: Depth of discharge
HESS: Hybrid energy storage systems
ESS: Energy storage systems
HRES: Hybrid renewable energy system
COE: Cost of energy
LPSP: Loss of power supply probability
PV: Photovoltaic panel
REF: Renewable energy fraction
RE: Renewable energy
RESs: Renewable energy resources
TGE: Total gas emission
NPC: Net present cost
SC: Supercapacitor
WT: Wind turbine
FC: Fuelcell
Elz: Electrolyzer
BG: Biomass gasifier
SESS: Supercapacitor energy storage systems
BESS: Battery energy storage system

## Symbols

${P}_{WT}(t)$: Power output of the wind turbine generator
${P}_{PV}\left(t\right)$: Power generation by photovoltaic panels
${P}_{SC}\left(t\right)$: Power generation by a supercapacitor
${P}_{BT}\left(t\right)$: Power generation by the battery
${P}_{L}(t)$: Load energy demand at time t
${P}_{ch}(t)$: Power available for battery charging
${P}_{distch}(t)$: Power to be discharged from the battery
${E}_{distch}\left(t\right)$: Energy discharged from the battery
${E}_{dump}\left(t\right)$: Energy dumped at time t
$$\upeta_{Inv}$$: Inverter efficiency
$$\upeta_{\_bat}$$: Battery efficiency
${E}_{ch}\left(t\right)$: Energy charged to the battery
${E}_{{b}_{min}}$: Energy stored in the battery (minimum)
${E}_{{b}_{max}}$: Energy stored in the battery (maximum)
${E}_{b}\left(t\right)$: Energy stored in the battery at time t
${E}_{SC}(t)$: Energy stored in the supercapacitor at time t
SOC: State of charge
SOC_min_: Lowest battery charge level
SOC_max_: Highest permissible battery charge level
O&M: Operation and maintenance costs
${E}_{grid\_p}\left(t\right)$: Energy purchased from the grid at time t
${E}_{grid\_s}\left(t\right)$: Energy sold to the grid at time t
t: Time
h: Hours
C_grid_: Cost of purchasing power from the grid
R_grid_: Revenue from selling energy to the grid
$\eta$: Supercapacitor efficiency factor
