# Supplementary material for: Optimal dimensioning of grid-connected PV/wind hybrid renewable energy systems with battery and supercapacitor storage a statistical validation of meta-heuristic algorithm performance
Source: Sci Rep. 2025 Dec 29;15:45658. doi: 10.1038/s41598-025-28234-9 (PMC12753802; doi:10.1038/s41598-025-28234-9)
Supplement: Supplementary file 1 — Supplementary Material 1 [file 41598_2025_28234_MOESM1_ESM.docx]

**Appendix A.**

Technical and economic characteristics of the HRES components

| **Components** | **Parameters** | **Value** | **Unit** |
| --- | --- | --- | --- |
| A. Solar Panel | Capacity of the solar panel | 0.345 | kW |
|  | Temperature coefficient of the solar panel | -0.390 |  |
|  | Productivity | 17.8 | % |
|  | Operating temperature | 44 | $℃$ |
|  | Derating factor | 82 | % |
|  | Capital cost | 550 | $/kW |
|  | Replacement cost of the solar panel | 550 | $/kW |
|  | O&M cost of the solar panel | 50 | $/Year |
|  | lifespan of the solar panel | 20 | Years |
| B. Wind Turbine | Rated power of the turbine | 1 | kW |
|  | Hub height of the turbine | 17 | m |
|  | Cost of capital | 880 | $/kW |
|  | Replacement cost of the wind turbine | 880 | $/kW |
|  | O&M cost of the wind turbine | 200 | $/kW |
|  | Lifespan of the wind turbine generator | 20 | Year |
|  | Cut-in speed | 3 | m/s |
|  | Cut-out speed | 35 | m/s |
|  | Rated wind speed | 10 | m/s |
| C. Battery | Capacity | 1 | kW |
|  | Capital cost of the battery | 167 | $/kW |
|  | Replacement cost of the battery | 167 | $/kW |
|  | O&M cost | 1.67 | $/kWh |
|  | Round-trip efficiency of the battery | 90 | % |
|  | Minimum battery charge status | 20 | % |
|  | Maximum depth of discharge life | 80 | % |
|  | Lifespan of the battery bank | 5 | Year |
| D. Supercapacitor | Capacity | 1 | kW |
|  | Capital cost | 748 | $/kW |
|  | O&M cost | 0.02 | $/kWh |
|  | Replacement cost | 523 | $/kW |
|  | Efficiency | 90 | % |
|  | Lifespan | 10 | Year |
| E. Inverter | Capacity of the inverter | 1 | kW |
|  | Replacement cost of the inverter | 300 | $/kW |
|  | Capital cost of the inverter | 300 | $/kW |
|  | O&M cost of the inverter | 50 | $/Year |
|  | Productivity of the inverter | 95 | % |
|  | Lifespan of the inverter | 20 | Year |
| F. Economic Parameters | Real interest | 2.07 | % |
|  | Project lifetime | 20 | Year |
